# The link between hyperuricemia and diabetes: insights from a quantitative analysis of scientific literature

**DOI:** 10.3389/fendo.2024.1441503

**Published:** 2025-02-07

**Authors:** Lili Ma, Jing Wang, Li Ma, Xian Min Wang

**Affiliations:** ^1^ Department of Internal Medicine, Shengzhou Hospital of Traditional Chinese Medicine, Shaoxing, China; ^2^ Xinjiang Laboratory of Respiratory Disease Research, Hospital of Traditional Chinese Medicine Affiliated to Xinjiang Medical University, Urumqi, China; ^3^ Department of Endocrinology, Affiliated Hospital of Traditional Chinese Medicine, Xinjiang Medical University, Urumqi, China; ^4^ Department of Scientific Research Management, Affiliated Hospital of Traditional Chinese Medicine, Xinjiang Medical University, Urumqi, China

**Keywords:** hyperuricemia, diabetes, scientometrics, VOSviewers, CiteSpace

## Abstract

**Background:**

Hyperuricemia (HUA) is a significant public health issue, ranking second only to diabetes in prevalence. While existing research demonstrates a robust correlation between these two conditions, the precise etiological mechanisms remain inadequately elucidated. This study utilized scientometric analysis to investigate the global association between HUA and diabetes.

**Methods:**

Data on HUA and diabetes were retrieved from the Web of Science Core Collection database, encompassing the period from its inception until September 30, 2024. Collaboration networks were examined using VOSviewer, cluster analysis was executed with CiteSpace, and systematic mapping was conducted using Bibliometrix.

**Results:**

By September 30, 2024, 1,464 studies indicated a consistent yearly increase in publications connecting HUA and diabetes despite some fluctuations. The lead authors were Richard J. Johnson, Miguel A. Lanaspa, and Masanari Kuwabara, with most contributors from China, the United States, and Japan. Key institutions include China Medical University, Shanghai Jiao Tong University, and Capital Medical University. The most published journal was Nutrition, Metabolism and Cardiovascular Diseases (CVDs), whereas the most cited journal was Diabetes Care. The reference network from 1987 to September 30, 2024, identified 19 clusters highlighting key research areas in HUA and diabetes, such as metabolic syndrome, uropathology, chronic kidney disease (CKD), and CVD. Exploring pathological mechanisms and pharmacological interventions linked to diabetes concomitant with HUA has emerged as a focal point of research and a burgeoning trend within the field.

**Conclusion:**

This study is the first scientometric analysis to synthesize research trends on HUA and diabetes, revealing molecular mechanisms and treatment strategies and providing theoretical insights for future clinical use.

## Graphical Abstract

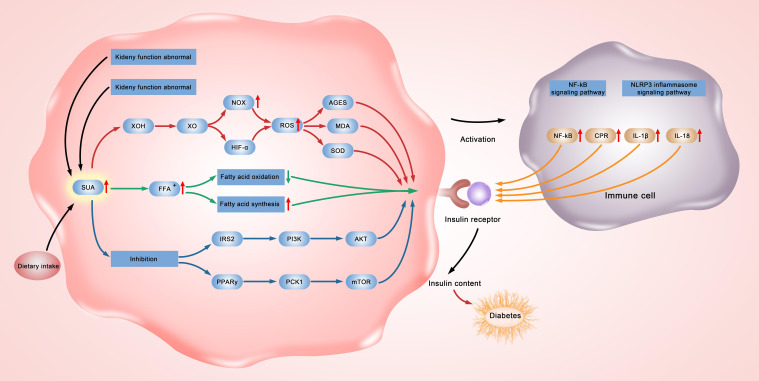

## 1 Introduction

Hyperuricemia (HUA) is a metabolic disorder marked by high uric acid levels due to increased production or decreased excretion during purine metabolism. In adult populations, HUA is typically diagnosed when serum uric acid concentrations exceed 6.0 mg/dL in females and 7.0 mg/dL in males (1). This condition is a significant public health issue, ranking second only to diabetes in prevalence. Research has indicated that HUA prevalence in Italy increased from 8.5% to 11.9% between 2005 and 2009 (2). Additionally, HUA incidence in China was reported to be 35.4% (3), while the international prevalence was noted to be 3.3% (4). A recent cohort study observed a strong link between high serum uric acid levels and type 2 diabetes (5). HUA prevalence among diabetic patients has been reported to be 21.24% in China (6) and 20.70% in North America (7). HUA might increase the risk of developing type 2 diabetes (8), leading to a higher incidence of diabetic nephropathy (9). Serum uric acid is the only diagnostic marker for HUA, acting as a key antioxidant and playing a role in obesity-related insulin resistance (10).

Recently, there has been a substantial increase in significant research concerning HUA and diabetes, leading some scholars to conduct thorough reviews and analyses of the relevant findings. Research on male urate oxidase knockout mice indicates that glucose metabolism issues lead to reduced insulin secretion and a higher risk of streptozotocin-induced diabetes (11). Zhao et al. (12) found that HUA in mice with metabolic disorders and insulin resistance increased inflammation, impaired glucose uptake, disrupted insulin signaling, and sped up diabetes progression. Yanai et al. (13) found that elevated urate transporter 1 and glucose transporter 9 (GLUT9) levels, combined with impaired glycolysis from insulin resistance, can lead to HUA. Lu et al. (14) further revealed that uric acid contributes to diabetes progression by hindering islet beta cell survival rather than directly triggering the disease. Yu W et al. (15) found that thioredoxin-interacting protein reduces oxidative stress and counters HUA-induced insulin resistance, suggesting it as a potential treatment target. Besides, Huang et al. (16) developed a HUA risk model for diabetic nephropathy patients, enhancing early detection and prevention in those with chronic nephropathy.

While past studies have explored the pathogenesis and treatment targets of HUA and diabetes, the exact link between them remains unknown, and quantitative analysis of literature in this field is lacking. Recently, bibliometrics has been used to bridge informatics and science (17). Progress in data visualization, text mining, and web analytics has created a new framework for synthesizing research evidence and impact (18). This framework used visual analysis and scientometrics to explore a field’s growth, knowledge structure, and academic exchange, helping identify key topics and development contexts essential for theoretical and practical advancements (19). Therefore, we conducted a scientometric study on HUA and literature on diabetes to analyze trends, networks, and authors, aiming to offer fresh insights and directions for research on their relationship.

## 2 Materials and methods

### 2.1 Data collection and retrieval methods

**Figure 1:**
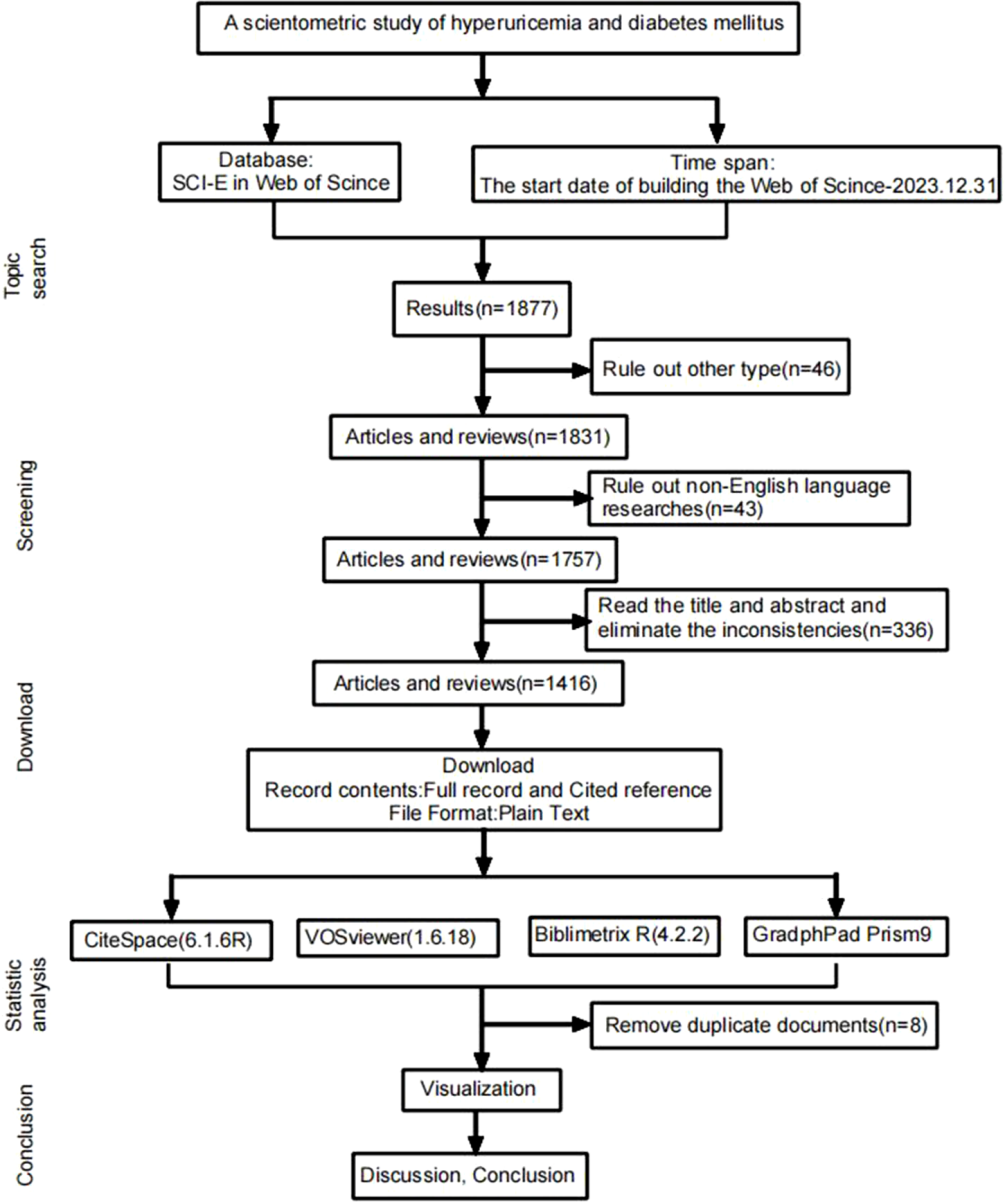
Document retrieval diagram.

Computer searches were performed in the Web of Science Core Collection using a Topic Search for terms related to “High uric acid” and “diabetes mellitus (DM),” including variations such as “type 2 DM” and “type 1 diabetes.” The search targeted English articles and reviews published until September 30, 2024. **Figure 1** depicts the retrieval process.

### 2.2 Data analysis

This study utilized the Biblimetrix R package (version 4.2.2) to gather essential data on national publishing networks and trending subjects. VOSviewer (version 1.6.18) facilitated the analysis of authors, research institutions, and national cooperative networks. CiteSpace (version 6.3.R1) analyzed word frequency, co-occurrence, and clusters for keywords, as well as co-citation for highly cited studies. Cluster analysis was weighted by the Log-likelihood rate algorithm. The time slice was set to one year, cosine measures correlation strength, and Top N = 50 was the selection threshold. Key nodes have centrality (C) greater than 0.1. Module value Q and network profile value S are essential parameters for measuring clustering structure rationality, with Q > 0.3 indicating significance. Each cluster was reviewed, and the automated labels were modified by the author if needed.

## 3 Results

### 3.1 Annual scientific production

**Figure 2:**
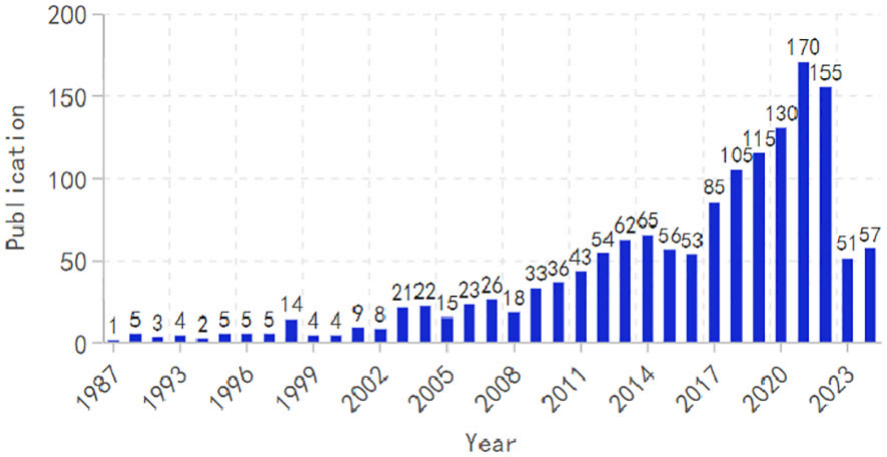
Annual publication trend chart.

**Figure 2** illustrates the annual publication trend, indicating a steady increase despite some fluctuations. The peak was in 2021, with 170 publications, underscoring the increasing global interest in research related to HUA and diabetes.

### 3.2 Authors analysis

**Figure 3:**
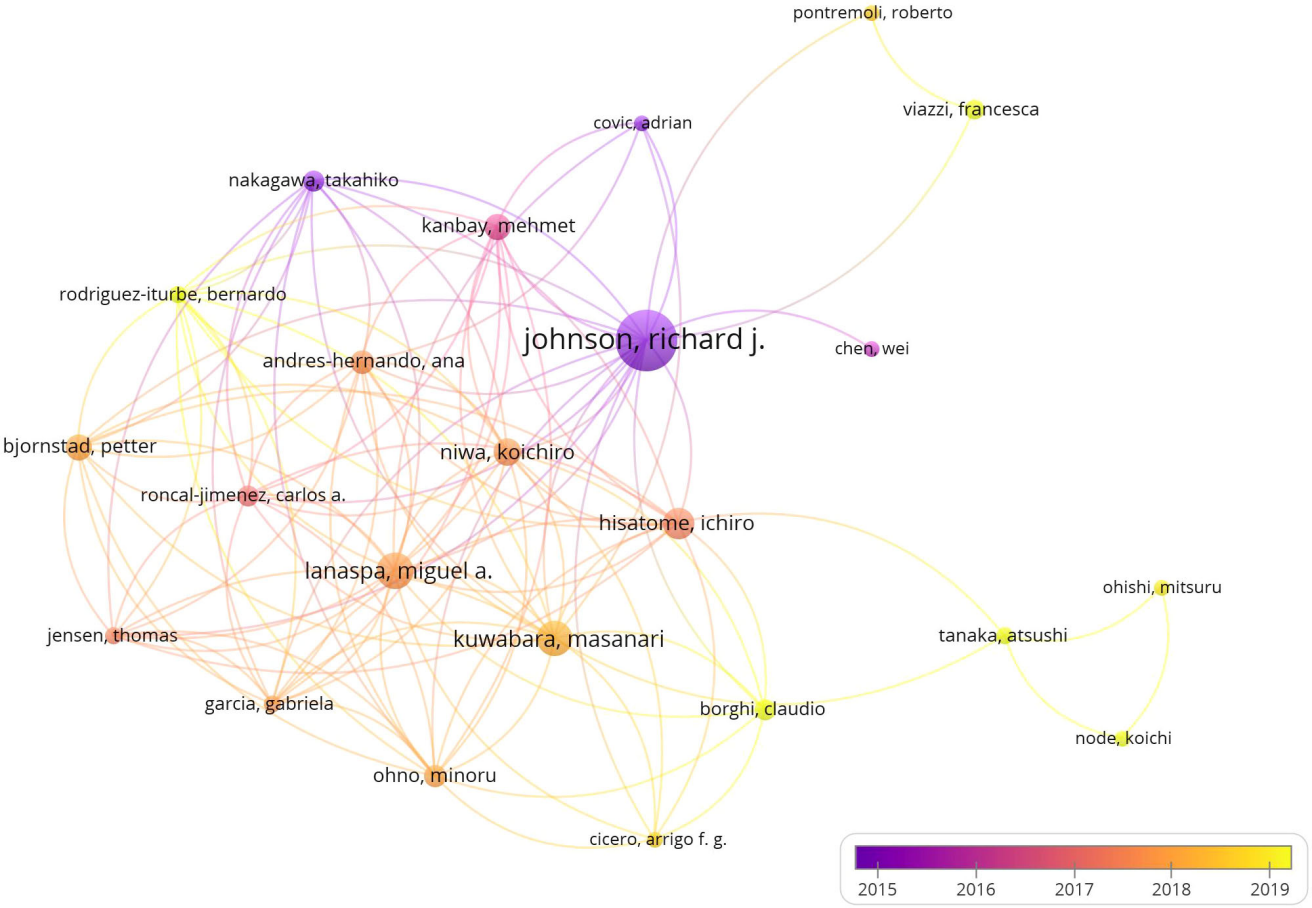
Author cooperation network diagram.

The study’s lead authors were Johnson, Richare J (41 publications), Lanaspa, Miguel A (19), and Kuwabara, Masanari (18). Bjornstad, Krishnan, and Kanbay, with 11 publications, have similar citation levels, highlighting their significant impact (**Table 1**). Visual analysis reveals extensive collaboration among authors (**Figure 3**).

### 3.3 Countries analysis

**Figure 4:**
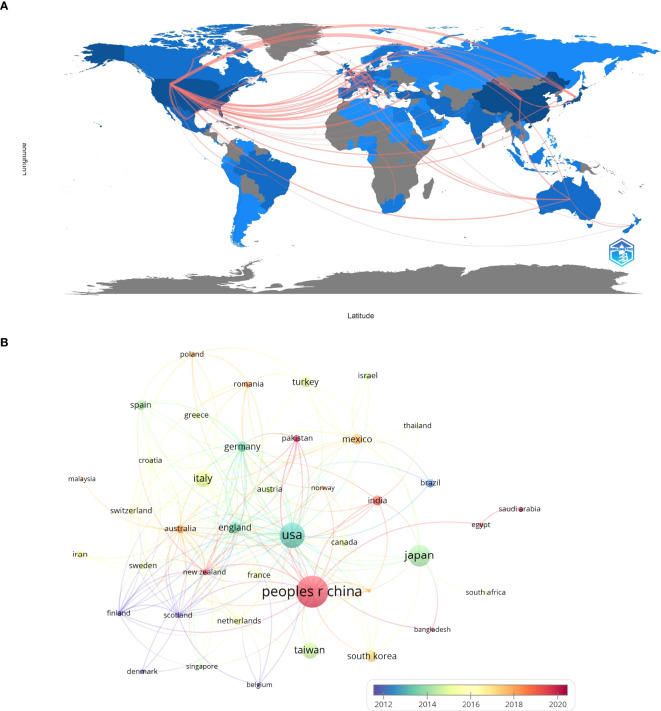
**(A)** Country collaboration map. **(B)** National cooperation network diagram.

The study found that China (n = 433), the United States (US) (n = 259), and Japan (n = 202) led in article publications. Although the US published fewer articles than China, it exhibits higher citation counts, suggesting superior research quality (**Table 2**). Visual analysis indicated active international cooperation, except for Egypt, Saudi Arabia, South Africa, and Malaysia (**Figures 4A, B**).

### 3.4 Organization analysis

**Figure 5:**
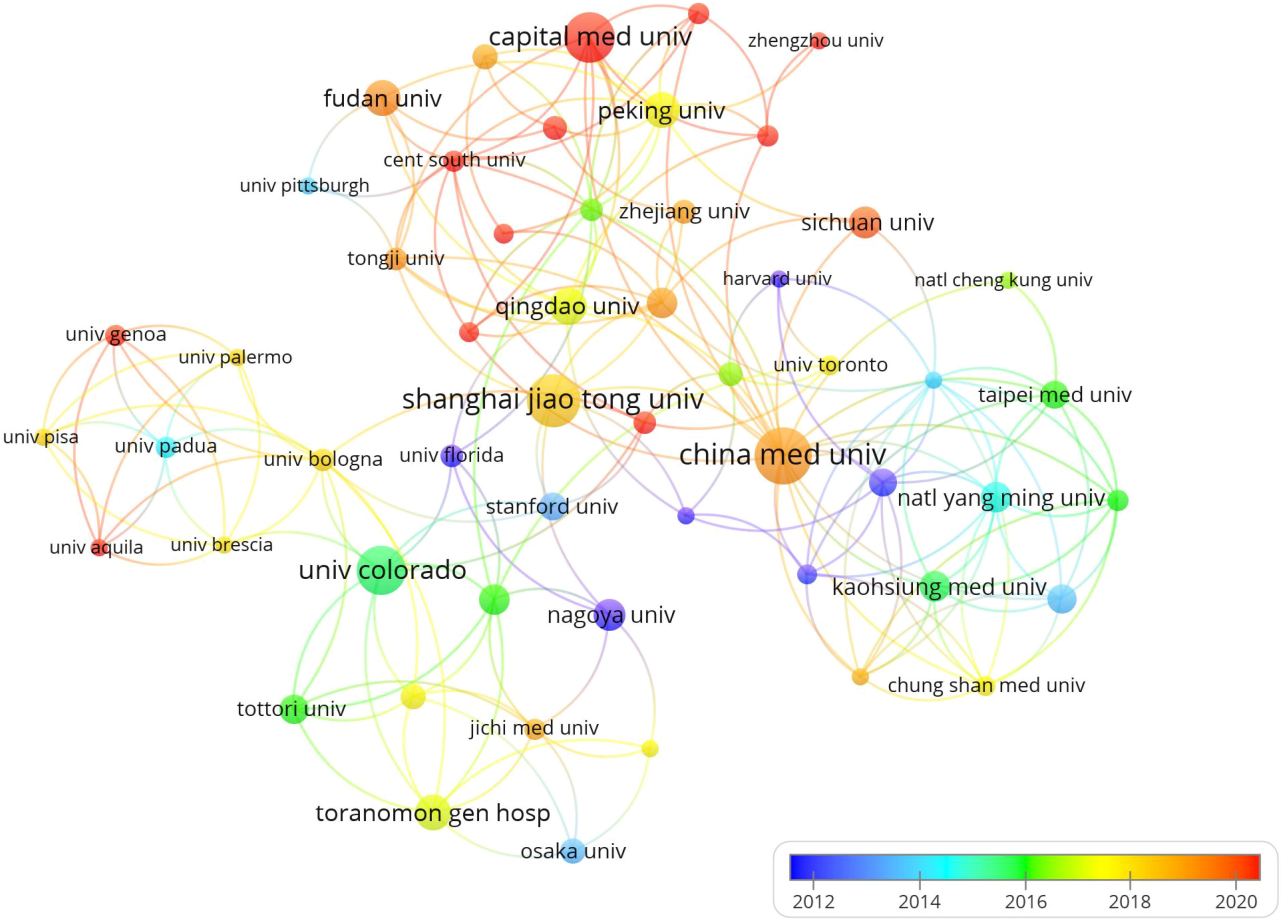
Organizational cooperation network diagram.

The study revealed that China Medical University (n = 36), Shanghai Jiao Tong University (n = 33), and Capital Medical University (n = 31) were top in article publications. The University of Colorado excelled with 2,225 citations indicating robust research activity (**Table 3**), while visual analysis indicated significant organizational collaboration (**Figure 5**).

### 3.5 Journal analysis

**Figure 6:**
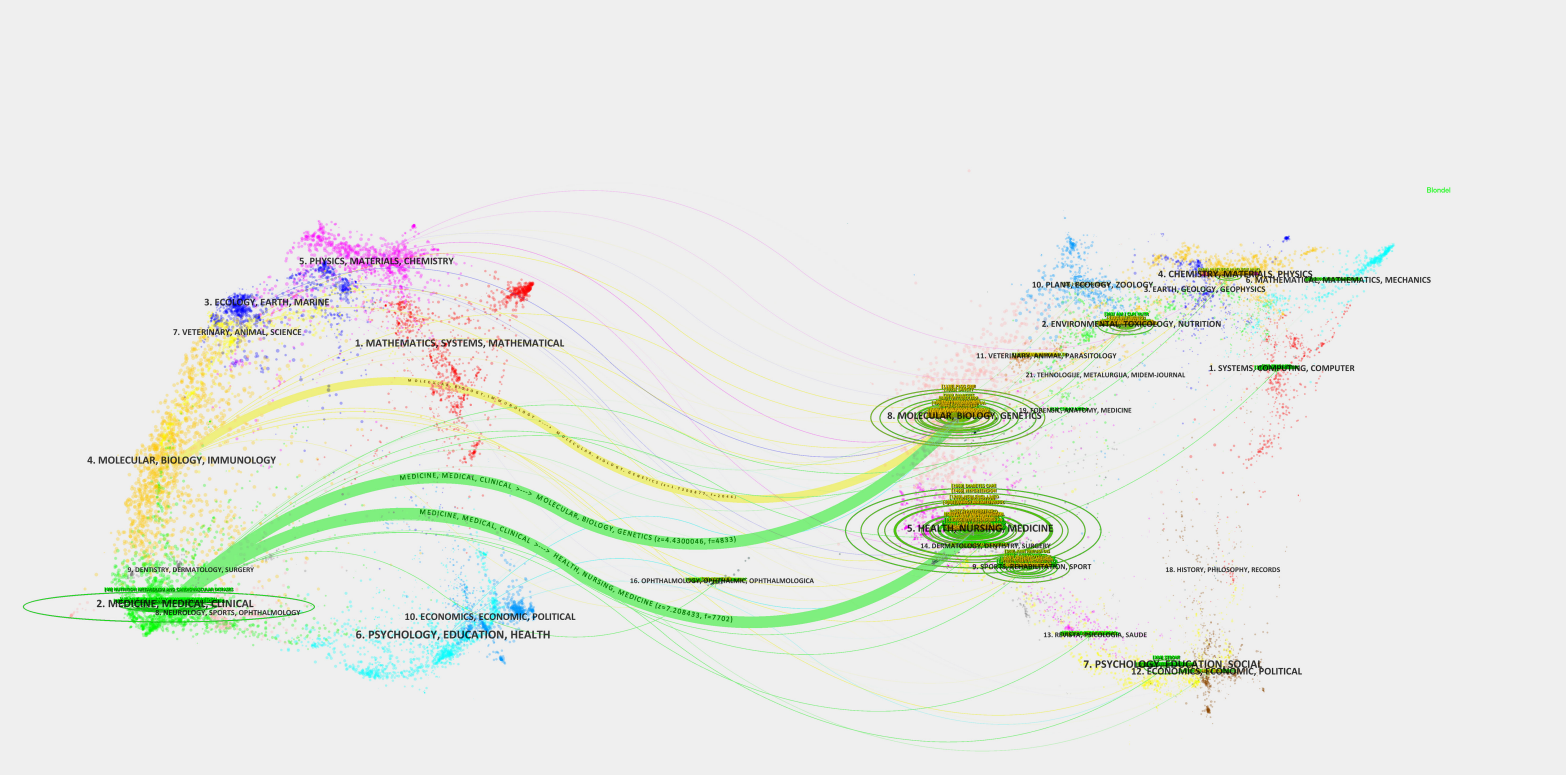
Journal’s dual map.

**Table 1** reveals that the highest publication volumes were from Nutrition Metabolism and Cardiovascular Diseases (CVDs), PLOS One, and Scientific Reports. **Supplementary Figure 1** highlights collaborative solid relationships among these journals. The most cited journals were Diabetes Care, Hypertension, and the New England Journal of Medicine (**Table 2**), with visual analysis indicating a strong co-citation correlation (**Supplementary Figure 2**). The journal’s dual map demonstrated citation links with co-citation journals, forming clusters on both sides. The green path highlighted the main reference route, indicating that Medlane, Medical, and Clinical journals mainly cite Molecular Biology, Genetics, Health, and Nursing (**Figure 6**).

**Table 1:** Top 10 journals by publication volume.

Number	Source	Documents	Citations	Total link strength	Impact factor	Zone
1	Nutrition Metabolism and Cardiovascular Diseases	49	611	7136	4.662	Q2
2	Plos One	41	1446	6206	3.752	Q2
3	Scientific Reports	33	500	4677	4.6	Q2
4	Diabetes Metabolic Syndrome and Obesity-targets and Therapy	17	56	2342	3.249	Q3
5	Hypertension Research	17	648	3065	5.528	Q2
6	Metabolism-clinical and Experimental	17	902	3414	13.934	Q1
7	Medicine	16	112	1759	1.817	Q3
8	Atherosclerosis	14	802	667	6.842	Q1
9	Frontiers in Endocrinology	14	38	1724	3.9	Q2
10	Nutrients	14	339	1651	4.8	Q1

**Table 2:** The 10 most cited journals.

Number	Source	Citations	Total link strength	Impact factor	Zone
1	diabetes care	1668	44577	17.152	Q1
2	hypertension	1466	42445	9.897	Q1
3	new engl j med	1243	36293	176.079	Q1
4	circulation	1160	36002	39.918	Q1
5	plos one	1138	32105	3.752	Q2
6	am j kidney dis	1037	31227	11.072	Q1
7	j am soc nephrol	1029	31397	14.978	Q1
8	lancet	1023	28216	202.731	Q1
9	jama-j am med assoc	1018	28463	157.335	Q1
10	kidney int	1010	30688	18.998	Q1

### 3.6 References analysis

**Figure 7:**
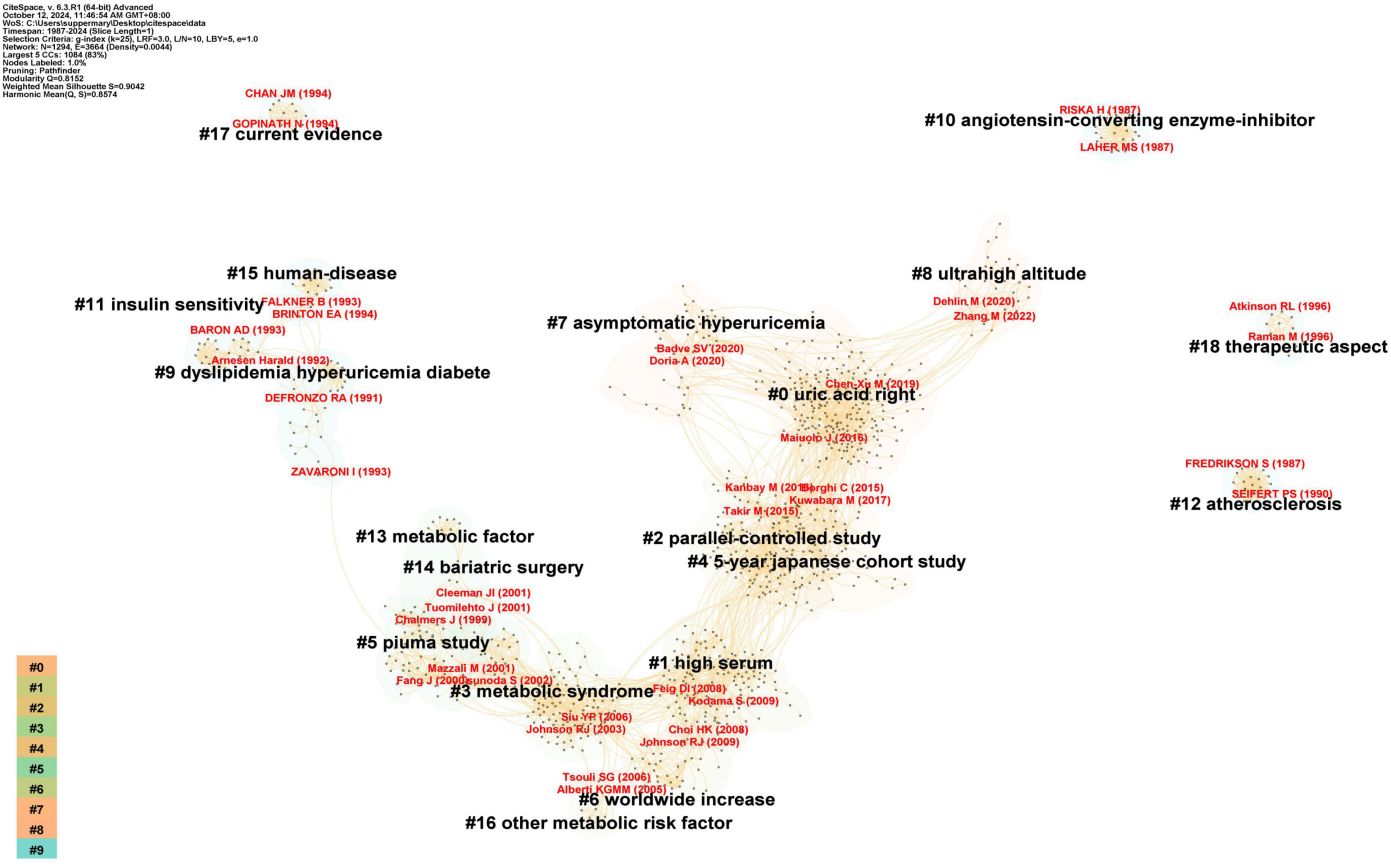
Co-citation references cluster analysis diagram.

**Figure 8:**
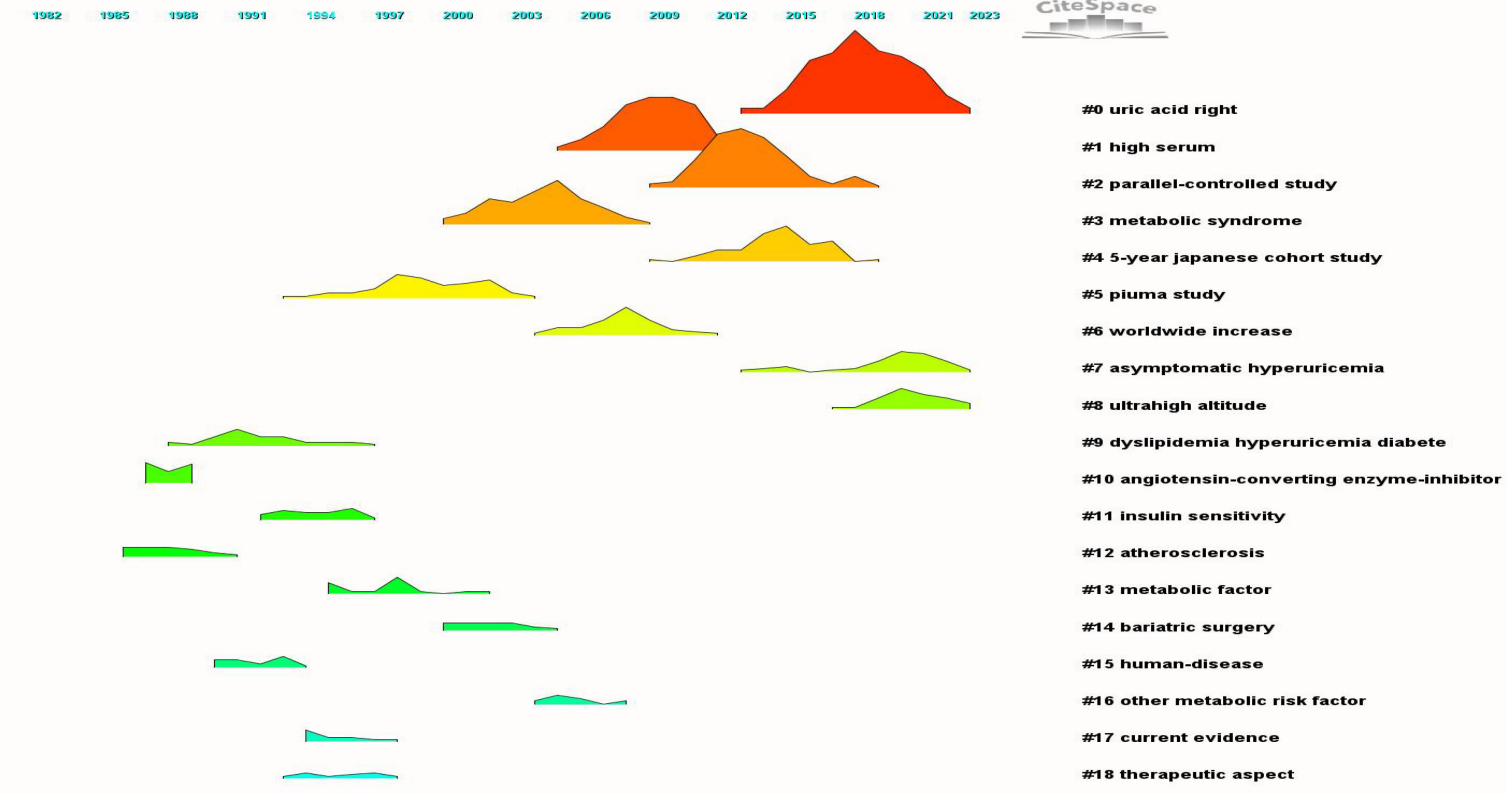
Co-citation references clustering peaks map.

**Figures 7**, **8** display 19 clusters emphasizing key research domains in HUA and diabetes, including metabolic syndrome, hypertension, CVD, and chronic kidney disease (CKD) (Q = 0.8152, S = 0.9042). Each cluster included a profile score, size, average publication year, and key references.

Between 1991 and 1994, pioneering research on “metabolic syndrome” delineated three distinct clusters: cluster #9, designated as “Dyslipidemia, HUA, and DM” (S = 0.918; 34; 1991) (20), cluster #11, labeled “Insulin sensitivity” (S = 0.995; 23; 1994) (21), and cluster #15, termed “Human Diseases” (S = 0.976; 17; 1991) (22). Over time, this research trajectory has progressed, culminating in the identification of additional clusters, including “metabolic factors” in cluster #13 (S = 0.991; 20; 1997) (23), “Other metabolic risk factors” in cluster #16 (S = 0.984; 12; 2005) (24), “Metabolic syndrome” in cluster #3 (S = 0.894; 105; 2004) (25), and ongoing developments such as cluster #6 “worldwide increase” (S = 0.944; 46; 2007) (26).

In 2001, research pertaining to the pathology of uric acid gained significant prominence, as evidenced by the evolution of cluster #5, designated as the “PIUMA Study” (S = 0.948; 66; 1999) (27), into cluster #1, referred to as “High Serum” (S = 0.893; 143; 2009) (28). This progression ultimately culminated in establishing cluster #0, which emerged as the most significant cluster within the network dedicated to the study of uric acid (S = 0.802; 228; 2018) (29).

A third research area emerged from cluster #12, “Atherosclerosis” (S = 1; 22; 1997) (30), and evolved into cluster #4, “5-YEAR JAPANESE COHORT STUDY” (S = 0.897; 140; 2014) (31), which includes chronic kidney and CVDs.

**Figure 9:**
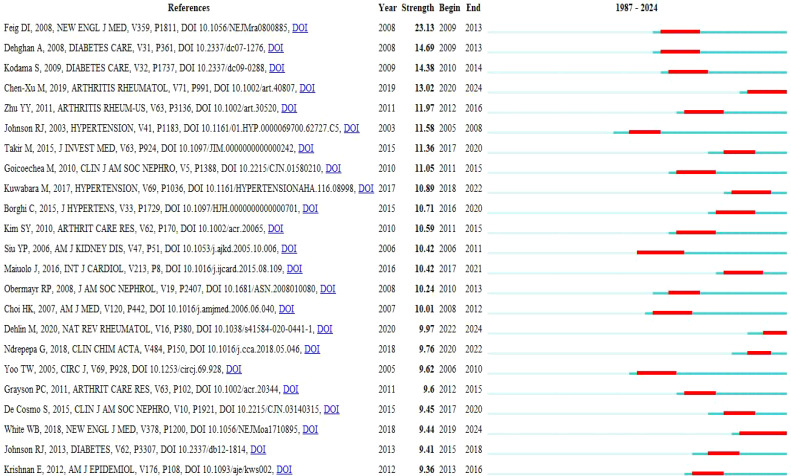
Top 25 references with the strongest citation bursts diagram.

Additionally, we conducted an emergent analysis of co-cited references from 1987 to September 30, 2024 (**Figure 9**). The analysis revealed that the three most recent and significant references on flare-ups were Chen-Xu M et al.’s cohort study on gout and HUA prevalence in the US (2007–2016) (32), White, W. B et al.’s study on cardiovascular safety of febuxostat or allopurinol in patients with gout (33), and Maiuolo J et al.’s review on uric acid metabolism regulation (1).

Inter-cluster link detection using network burst dynamics from 1987 to September 30, 2024 (Annex 1).

### 3.7 Most cited references

**Table 3** indicates that the 10 most cited references included Feig DI et al.’s comprehensive analysis of the biological function of uric acid and the specific mechanism of metabolic syndrome induced by HUA (34). Chen-Xu M et al.’s cohort study on gout and HUA prevalence in the US (2007–2016) (32), and Kuwabara M et al.’s cohort study examined the association between asymptomatic HUA and cardiometabolic diseases (35).

**Table 3:** The 10 most cited references.

Citation Counts	Cited References	Source	Vol	Page	Title	Type of paper	DOI	Cluster ID
47	Feig DI (2008) (34)	NEW ENGL J MED	V359	P1811	Uric Acid and Cardiovascular Risk	Review	10.1056/NEJMra0800885	1
35	Chen-Xu M (2019) (32)	ARTHRITIS RHEUMATOL	V71	P991	Contemporary Prevalence of Gout and HUA in the United States and Decadal Trends: The National Health and Nutrition Examination Survey, 2007-2016	Cohort Study	10.1002/art.40807	0
31	Kuwabara M (2017) (35)	HYPERTENSION	V69	P1036	Asymptomatic HUA Without Comorbidities Predicts Cardiometabolic Diseases: Five-Year Japanese Cohort Study	Cohort Study	10.1161/HYPERTENSIONAHA.116.08998	4
31	Kodama S (2009) (36)	DIABETES CARE	V32	P1737	Association between serum uric acid and development of type 2 diabetes	Cohort Study	10.2337/dc09-0288	1
30	Dehghan A (2008) (37)	DIABETES CARE	V31	P361	High serum uric acid as a novel risk factor for type 2 diabetes	Cohort Study	10.2337/dc07-1276	1
27	Maiuolo J (2016) (1)	INT J CARDIOL	V213	P8	Regulation of uric acid metabolism and excretion	Review	10.1016/j.ijcard.2015.08.109	0
26	White WB(2018) (33)	NEW ENGL J MED	V378	P1200	Cardiovascular Safety of Febuxostat or Allopurinol in Patients with Gout	RCT	10.1056/NEJMoa1710895	0
25	Zhu YY (2011) (38)	ARTHRITIS RHEUM-US	V63	P3136	Prevalence of gout and HUA in the US general population: the National Health and Nutrition Examination Survey 2007-2008	Cohort Study	10.1002/art.30520	1
24	Takir M (2015) (39)	J INVEST MED	V63	P924	Lowering Uric Acid With Allopurinol Improves Insulin Resistance and Systemic Inflammation in Asymptomatic HUA	RCT	10.1097/JIM.0000000000000242	2
24	Zhao YM (2018) (40)	DIABETES OBES METAB	V20	P458	Effects of sodium-glucose co-transporter 2 (SGLT2) inhibitors on serum uric acid level: A meta-analysis of randomized controlled trials	RCT	10.1111/dom.13101	0

### 3.8 Keyword analysis

**Figure 10:**
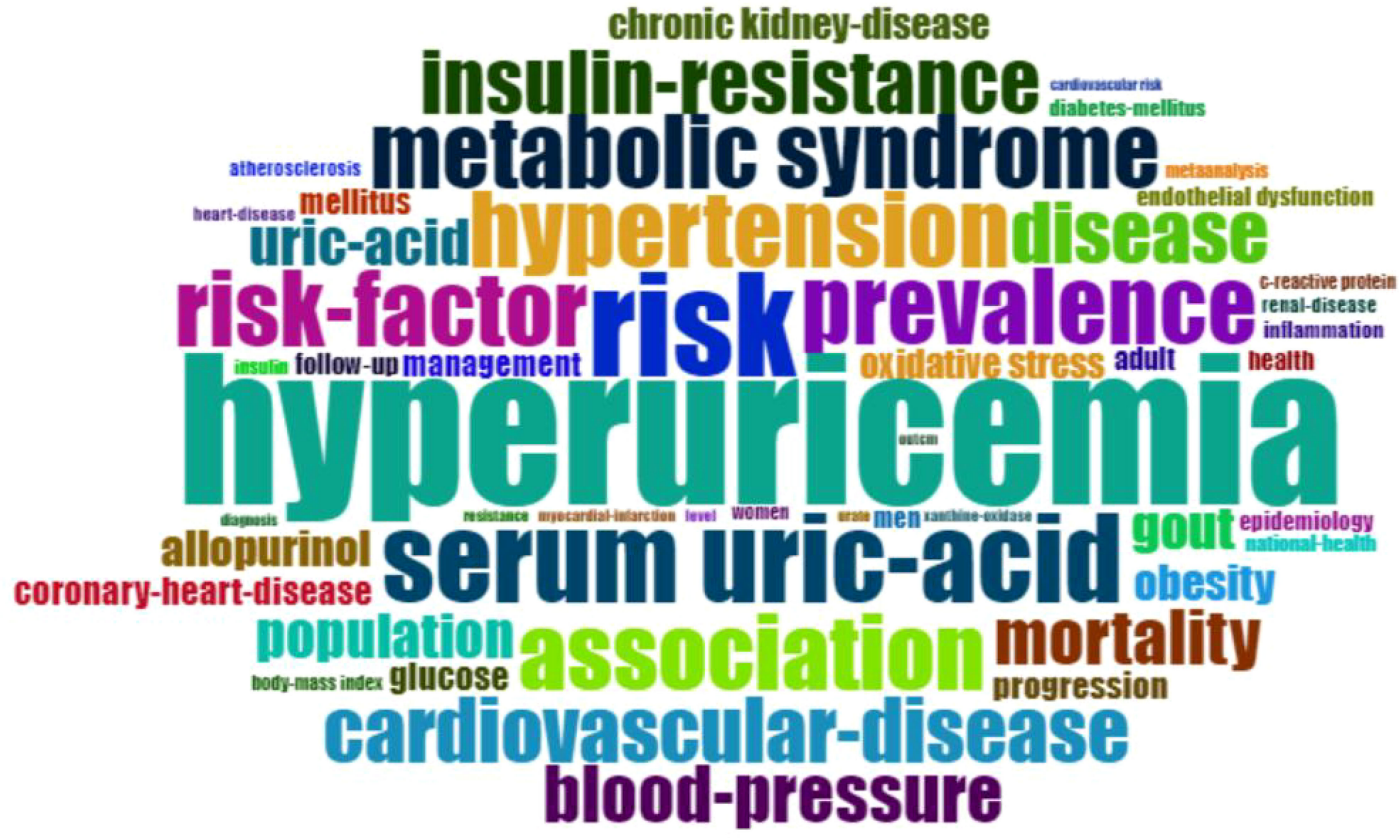
Keywords word cloud diagram.

Keyword frequency was crucial for identifying research trends and choosing search terms for literature reviews. The network diagram indicated that apart from “HUA,” “serum uric acid,” and “diabetes,” the most common terms were metabolic syndrome, hypertension, insulin resistance, CVD, and CKD (**Figure 10**).

**Figure 11:**
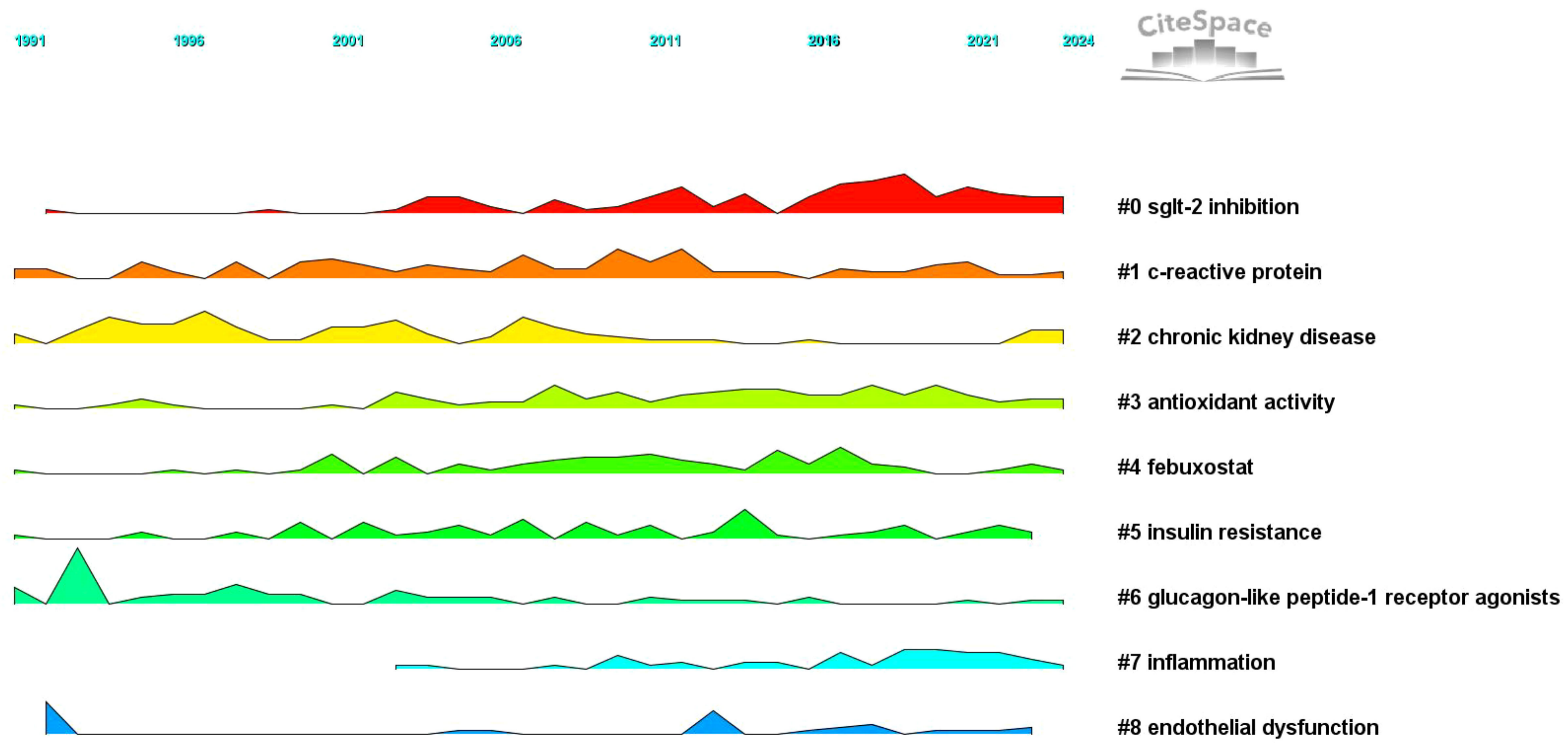
Keyword clustering peaks map.

**Figure 12:**
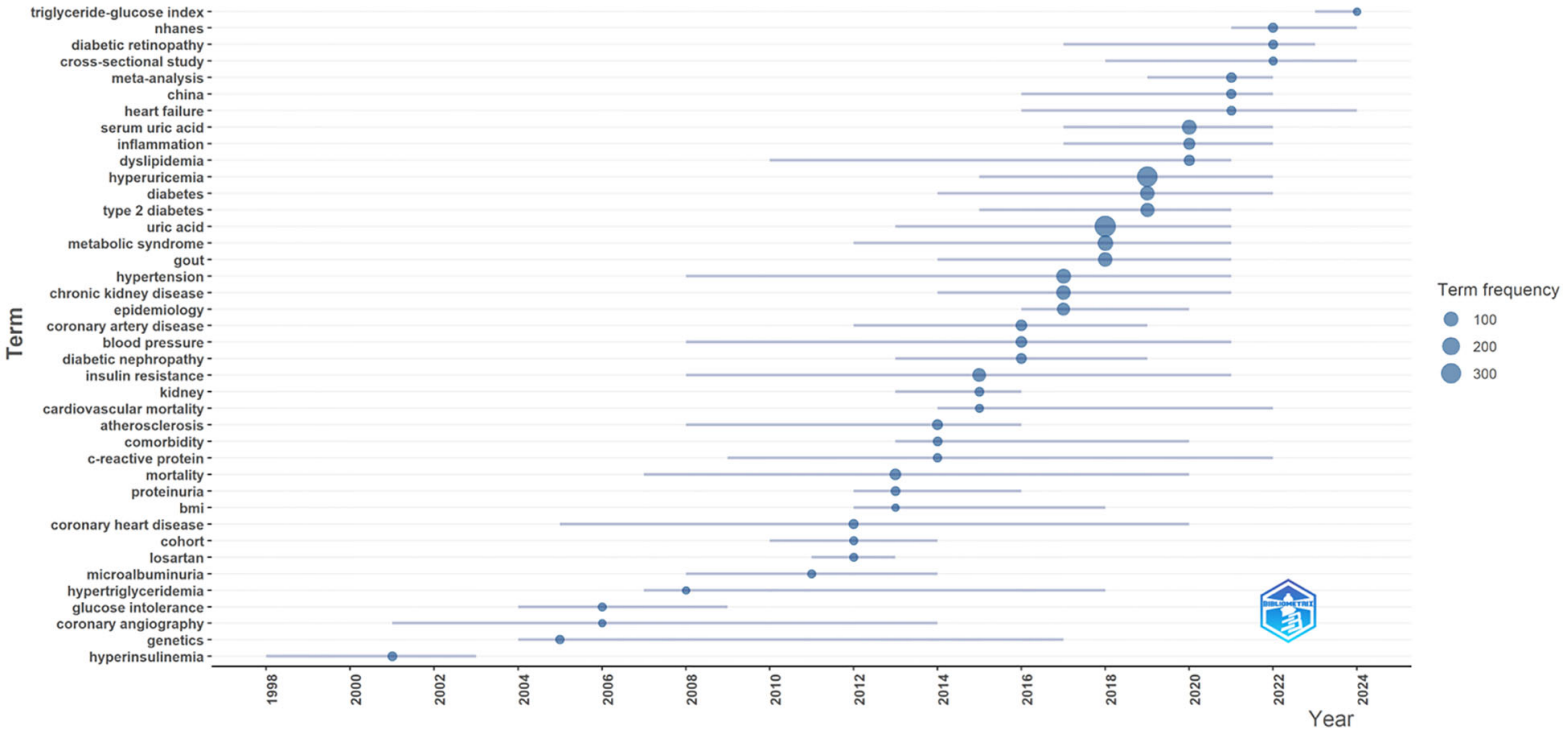
Trend topics diagram.

**Figure 13:**
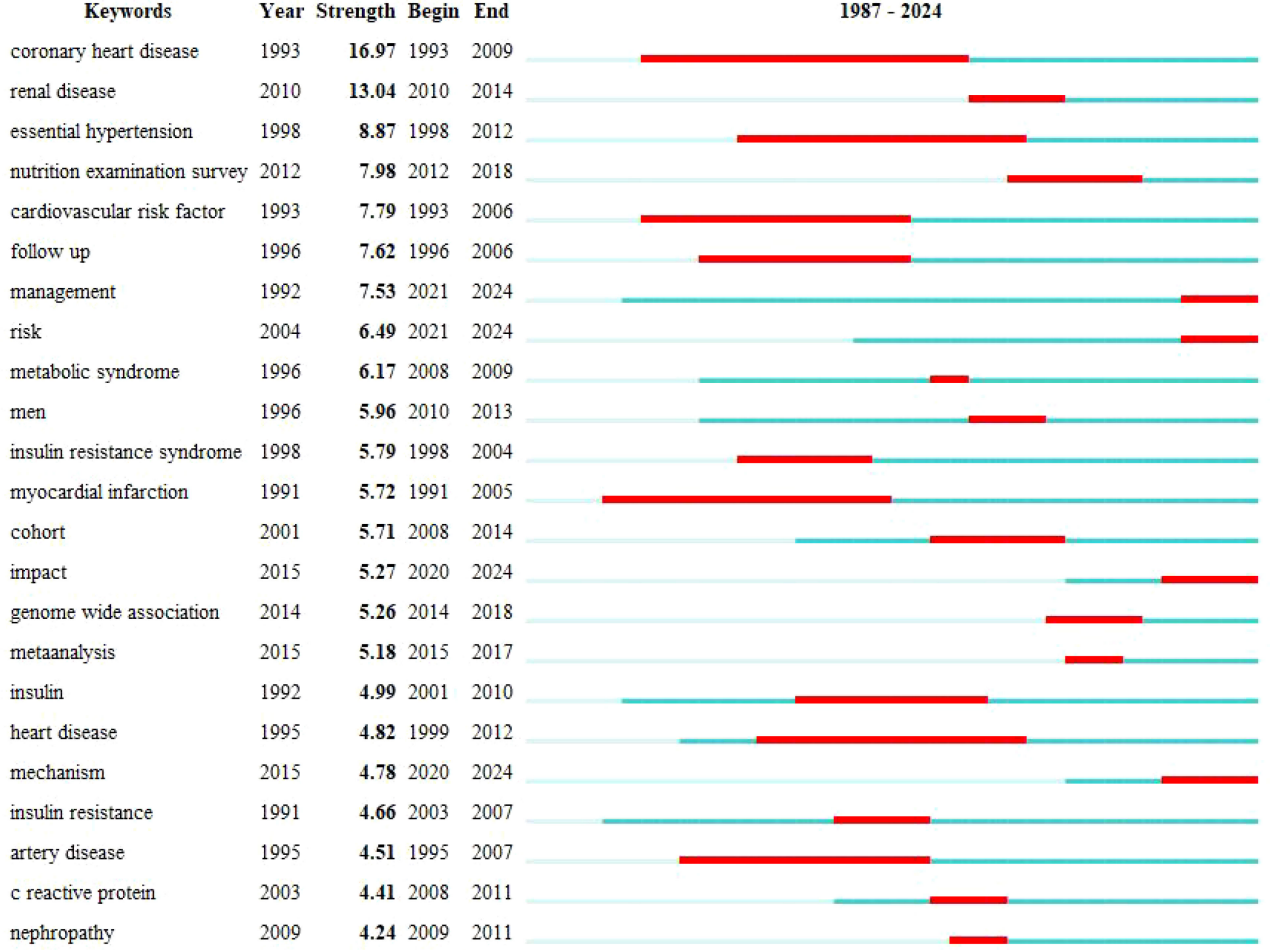
Top 25 keywords with the strongest citation bursts diagram.

The peak map identifies nine distinct clusters after re-labeling the automatic labels from the keyword cluster analysis: “ Sodium-dependent glucose transporter 2 (SGLT-2) inhibition,” “C-reactive protein (CRP),” “CKD,” “inhibition,” “antioxidant activity,” “febuxostat,” “insulin resistance,” “glucagon-like peptide-1 receptor agonists,” “inflammation,” and “endothelial dysfunction.” The network exhibits strong clustering and modularity (Q = 0.4258, S = 0.711; **Figure 11**). An analysis of trending topics over the past five years revealed research focus on the pathogenesis of HUA, DM, and associated conditions such as triglyceride-glucose index, diabetic nephropathy, inflammation, and heart failure (**Figure 12**). The top 25 co-cited keywords were identified through emergent analysis, with recent citation bursts highlighting terms such as “nutrition examination survey,” “impact,” “genome-wide association,” and “mechanism” (**Figure 13**).

## 4 Discussion

This study represents the inaugural comprehensive scientometric analysis of HUA and diabetes. As illustrated in **Figure 1**, scholarly research on this subject has demonstrated a consistent annual increase since the establishment of the Web of Science Core Collection, with a marked surge in activity from 2017 to 2022, thereby attracting substantial academic interest. The collaborative network analysis revealed robust partnerships among authors, institutions, and countries, with Johnson emerging as a significant contributor. The US was at the forefront in terms of research quality and international impact. Furthermore, it is noteworthy that 70% of the research originated from Chinese universities. The journal network analysis revealed that among the top 10 journals, **“**Metabolism-Clinical and Experimental” possesses the highest impact factor at 13.934 (Q1), followed by “Atherosclerosis” with an impact factor of 6.842 (Q1). Although these two journals publish fewer articles than “Nutrition, Metabolism and CVDs, PLOS One, and Scientific Reports,” they are recognized for their exceptional quality and substantial impact in the field. Excluding “PLOS One,” the top 10 journals ranked by total citations were classified as Q1 and acknowledged as high-quality international publications. These journals established robust co-citation networks and significantly contributed to the recognition and advancement of research on HUA and diabetes. The journal’s dual map overlay analysis revealed that articles in Medlane, Medical, and Clinical journals are mainly cited in Molecular, Biology, Genetics, Health, Medlane, and Nursing journals. This indicates that most research on the link between HUA and diabetes is found in clinical journals, with little presence in immunology, molecular, and biological publications. This highlights the need for more fundamental research to understand the precise molecular mechanisms involved.

### 4.1 Research basis

The findings presented in **Table 3** indicate that the 10 most frequently cited articles had laid the foundational framework for research on HUA and diabetes. Chen-Xu et al. (32) reported HUA prevalence rates of 20.2% in adult males and 20.0% in females from 2007 to 2016, while Zhu et al. (38) found an average prevalence of 21.4% before 2008, linked to rising obesity. Both studies highlight an increasing trend of HUA in the US. Feig et al. (34) and Maiuolo et al. (1) studied the biological roles and metabolic syndrome caused by HUA, along with the enzymatic pathways that regulate uric acid. Their work has facilitated further research. Dehghan et al. (37) found that 10.2% of people with high serum uric acid levels were diagnosed with diabetes in a retrospective study. A meta-analysis (35) found that each 1 mg/dL increase in serum uric acid levels is linked to a 17% higher risk of type 2 diabetes, suggesting that uric acid could be a useful predictive marker for the disease and warrants further study. Takir et al. (39) discovered that allopurinol reduces uric acid, improving inflammation and insulin resistance in patients with asymptomatic HUA, but its role in preventing type 2 diabetes remains unclear. Borghi et al. (41) found that lowering serum uric acid through xanthine oxidase inhibitors, such as allopurinol and febuxostat, is the best treatment for cardiovascular and renal diseases. Goicoechea et al. (42) highlighted allopurinol’s benefits in slowing CKD and reducing cardiovascular hospitalization risk. Kuwabara et al. (35) proposed that HUA could be a useful biomarker for predicting cardiometabolic disorders, opening new research opportunities.

### 4.2 Research trends

**Figure 10** demonstrates the frequent occurrence of metabolic syndrome, hypertension, CVD, and CKD in the keyword network analysis, suggesting that diabetes with HUA may increase the risk of these conditions. We then explored the mechanisms behind this, as detailed below.

#### 4.2.1 Metabolic syndrome

HUA is recognized as a significant element of metabolic syndrome, which is a critical risk factor for CVD and type 2 diabetes (43). Empirical evidence indicates that elevated uric acid levels in individuals with diabetes may contribute to the onset of metabolic syndrome through multiple mechanisms. Primarily, insulin resistance serves as a crucial connection between diabetes and HUA. Insulin resistance can result in dysregulated glucose metabolism, leading to hyperglycemia, which elevated uric acid levels may further aggravate through their impact on insulin signaling pathways (44). A study found (45) that high uric acid levels are significantly linked to increased body mass index, fasting blood glucose, and insulin resistance (HOMA-IR), suggesting that elevated uric acid may worsen insulin resistance in diabetic patients. Second, inflammatory responses are crucial in linking diabetes, HUA, and metabolic syndrome. Elevated uric acid levels cause chronic low-grade inflammation, closely tied to obesity, diabetes, and CVD. Uric acid acts as a pro-inflammatory agent, activating pathways that impact fat metabolism and insulin signaling (46). For example, studies indicate that high uric acid levels correlate with increased highly sensitive CRP, highlighting a link between inflammation and these diseases (47). This inflammation can negatively impact vascular health and elevate cardiovascular risk in individuals with diabetes.

Moreover, abnormal lipid metabolism links diabetes and HUA, with people with diabetes often indicating high triglycerides and low high-density lipoprotein (HDL) levels. HUA can worsen these lipid abnormalities (48). This changed lipid metabolism raises the risk of CVD and may lead to metabolic syndrome. A study on a Mediterranean population identified low HDL cholesterol (HDL-C) and high blood sugar as independent predictors of HUA (43), indicating their potential combined role in developing metabolic syndrome. Finally, genetic and environmental factors influence the link between diabetes, HUA, and metabolic syndrome. Genetic variants can increase susceptibility, while unhealthy lifestyles heighten these risks (49). Consequently, understanding the diabetes-HUA connection is crucial for preventing and managing metabolic syndrome.

#### 4.2.2 Hypertension

In individuals with diabetes, elevated uric acid levels may influence blood pressure through various mechanisms. Initially, insulin resistance, commonly observed in this population, can result in diminished renal excretion of uric acid, thereby contributing to the development of HUA (50). HUA is an independent risk factor for hypertension, potentially raising blood pressure by causing endothelial dysfunction (51) and activating the renin-angiotensin-aldosterone system (RAAS) (52), both of which lead to blood vessel constriction and sodium retention. Moreover, diabetes-related chronic inflammation can worsen hypertension by encouraging vascular cell growth and arteriosclerosis (53). HUA, a pro-inflammatory factor, exacerbates this by triggering inflammation in vascular cells, impairing blood vessel dilation (54).

Subsequently, people with diabetes frequently experience obesity and metabolic syndrome, which worsen the link between HUA and hypertension. Obesity promotes the release of pro-inflammatory factors from adipose tissue, worsening insulin resistance and HUA (45). It also raises blood volume, contributing to higher blood pressure (55). For instance, individuals with diabetes often have elevated triglycerides and low HDL-C, both of which are closely associated with increased blood pressure. In diabetic patients, HUA may be linked to kidney ultrafiltration. Early-stage diabetes can cause renal tubule hyperfiltration, reducing the kidney’s ability to excrete uric acid and worsening HUA (56). This kidney function change can also contribute to hypertension.

#### 4.2.3 CVD and CKD

The coexistence of CKD and CVD increases mortality risk and negatively affects quality of life. Research indicates that high uric acid levels are closely linked to metabolic syndrome conditions such as obesity, hypertension, and diabetes, which are independent risk factors for CKD and CVD (57). First, the inflammatory response significantly contributes to disease development. HUA is an independent risk factor for CVD and CKD, potentially worsening renal and cardiovascular damage in people with diabetes by activating the RASS, inhibiting nitric oxide synthesis, and promoting vascular lesions (58). Besides, inflammation may increase cardiovascular risk by causing endothelial dysfunction and speeding up atherosclerosis (59). In diabetic individuals, an increased inflammatory response combined with HUA significantly raises the risk of CKD. A study revealed that diabetic patients with both HUA and hypertension faced a 5.42 times higher risk of CKD compared to those with just one of these conditions (60). Inflammatory mediators such as tumor necrosis factor-alpha (TNF-α) and interleukin 6 (IL-6) are frequently elevated in diabetes and HUA, promoting inflammation and linking to insulin resistance and metabolic syndrome (61). They can also damage the heart and kidneys by triggering pro-inflammatory pathways, raising the risk of heart failure and CKD (62). Inflammation also impairs renal tubule function, causing cell apoptosis and fibrosis, which speeds up CKD progression (63).

Overall, the interplay of inflammation, uric acid, and blood pressure is crucial for cardiovascular and kidney health in people with diabetes. Second, insulin resistance can notably raise the risk of cardiovascular and CKD in diabetic and hyperuricemic patients through various mechanisms. For example, insulin resistance boosts 11β-hydroxy steroid dehydrogenase 1 in the liver, enhancing glucocorticoid activity and worsening insulin resistance. This results in increased glucose production and fat formation, aggravating metabolic disorders (64), including hyperglycemia and hyperlipidemia, and becoming a significant risk factor for CVD (65). Moreover, research indicates that insulin resistance can exacerbate HUA by boosting uric acid production and decreasing its excretion (66). It also causes endothelial dysfunction, oxidative stress, and inflammation, contributing to CVD and kidney damage (66). High uric acid levels in patients with CKD can worsen heart remodeling and fibrosis, increasing cardiovascular risk (67). Therefore, the combination of insulin resistance and HUA may significantly heighten the risk of cardiovascular and CKDs. Finally, oxidative stress plays a crucial role in increasing the risk of cardiovascular and CKDs in patients with diabetes and HUA. It triggers the release of inflammatory mediators, leading to chronic inflammation, which is closely linked to the development and progression of these diseases (68). High uric acid levels can increase oxidative stress and cause cell damage, particularly in diabetic patients (69).

Similarly, people with diabetes often have elevated inflammatory factors such as TNF-α and IL-6, which harm the vascular endothelium and promote atherosclerosis (70). In HUA, uric acid buildup causes oxidative stress in kidney cells, leading to cell death and kidney damage, which can impair kidney function and heighten CVD risk (71). Oxidative stress also encourages vascular smooth muscle cell growth and movement, contributing to vascular remodeling and atherosclerosis, especially in diabetic patients with HUA, increasing cardiovascular events (72). Oxidative stress is closely linked to metabolic syndrome, such as insulin resistance and lipid issues, which elevate the risk of cardiovascular and CKDs (58). In summary, inflammation, insulin resistance, and oxidative stress may increase the risk of cardiovascular and CKDs in diabetic patients with HUA.

## 5 Hot topics

**Figures 11**–**13** illustrate that research concerning the relationship between diabetes and HUA was increasingly directed towards elucidating the underlying mechanisms and investigating pharmacological interventions. Subsequently, this study examines the pathological mechanisms that connect HUA and diabetes and evaluates effective therapeutic agents.

### 5.1 Potential pathological link between HUA and diabetes

#### 5.1.1 Insulin resistance

Numerous studies have clarified the link between uric acid and insulin resistance. Wan X et al. (73) found that uric acid activates the NOD-like receptor protein 3 (NLRP3) inflammasome, leading to liver fat accumulation and insulin resistance, while inhibiting NLRP3 reduced these effects. Yu W et al. (74) reviewed how soluble uric acid induces insulin resistance in different tissues, suggesting a new treatment target for HUA-induced insulin resistance in 2023. A recent study indicates that HOMA2 IR-CP more effectively assesses the link between insulin resistance and uric acid levels (75). Insulin resistance contributes to type 2 diabetes through oxidative stress, mitochondrial dysfunction, lipid accumulation, and inflammation, involving increased insulin receptor substrate (IRS) protein phosphorylation, IRS-1 degradation through the mammalian target of rapamycin (mTOR) pathway, reduced activation of signaling molecules such as phosphatidylinositol 3-kinase and protein kinase B (AKT), and heightened activity of phosphatases such as protein Tyrosine Phosphatases (PTPs), phosphatase and Tensin Homolog (PTEN), and protein Phosphatase 2A (PP2A). A study on mice found that high uric acid levels can speed up diabetes onset by damaging islet cells, indirectly causing the disease (76). This suggests a strong connection between diabetes and HUA due to insulin resistance impacting glucose uptake and uric acid excretion.

#### 5.1.2 Inflammatory reaction

A 2020 study discovered that asymptomatic high uric acid levels can lead to diabetes by causing inflammation through urate crystals (77). Recent research indicates that soluble uric acid can change cell structure and pH, increasing inflammation and neutrophil activity (75). The studies suggest that soluble uric acid may contribute to inflammation in HUA and diabetes, providing insights into their mechanisms. Elevated HUA levels can trigger inflammation and impair glucose metabolism through pathways such as nuclear factor kappa B (NF-κB) and NLRP3. A mouse study indicated that high HUA levels may cause insulin resistance and decreased insulin secretion, potentially leading to type 2 diabetes (78). Uric acid’s inflammatory nature may drive this effect, as indicated by higher TNF-α levels and enzyme activation post-infusion. High uric acid levels can trigger pathways such as NF-κB and NLRP3 inflammasome, leading to inflammation and endothelial cell damage. This inflammation impacts blood vessels and insulin receptors, particularly in obese diabetic patients. Furthermore, inflammatory cells in fat tissue release cytokines, causing oxidative stress, inflammation, and increased HUA.

#### 5.1.3 Oxidative stress

Oxidative stress significantly influences serum uric acid levels by affecting urate production, excretion, and reabsorption (79). High uric acid levels can induce oxidative stress by converting xanthine dehydrogenase (XDH) to xanthine oxidase (XO), activating NADPH oxidases (NOX) and hypoxia-inducible factor-1α (HIF-1α), and inhibiting antioxidant enzymes, resulting in more free radicals and immune cell activation. Additionally, HUA triggers endoplasmic reticulum stress and boosts reactive oxygen species (ROS) production, causing oxidative stress in this protein-synthesizing organelle. This sequence of events boosts free radicals and oxides, causing lipid, protein, and deoxyribonucleic acid oxidation, leading to cell death and inflammation. Indicators of oxidative stress include higher levels of malondialdehyde, peroxynitrosoides, advanced glycation end products, and lower superoxide dismutase (80), which can damage beta cells, induce insulin resistance, and speed up diabetes progression. Studies have indicated that diabetic hyperglycemia can generate oxidative stress, raising lipid peroxidation products (31). This may reduce the number or effectiveness of intracellular insulin receptors, interfere with insulin signaling, and impair the insulin pathway. Consequently, insulin signaling weakens, antioxidant capacity decreases, and ROS production rises during uric acid synthesis, leading to elevated uric acid levels. These studies indicate that people with high uric acid levels and diabetes are more susceptible to oxidative stress, suggesting new treatment approaches for managing uric acid and enhancing health and quality of life.

#### 5.1.4 Kidney injury

The kidney is crucial in HUA and diabetes development, with uric acid influencing factors that lead to kidney damage. The study found that uric acid increases growth factor production and activators while reducing nitric oxide synthesis and endothelial cell growth (81). Elevated HUA levels can induce insulin resistance impacting insulin receptors, signaling pathways, and glucose transporter protein 4 (GLUT4) expression, potentially causing kidney damage and increasing diabetes risk. Diabetic hyperglycemia worsens this by enhancing protein binding to uric acid and reducing its excretion, raising blood uric acid levels. Recent research suggests that uric acid can harm kidneys by causing inflammation, endothelial dysfunction, and overactivating the renin-angiotensin-aldosterone system (RASS), worsening HUA and diabetes (82). Patients should manage blood sugar, reduce uric acid, and address oxidative stress and inflammation to protect kidney health and prevent complications.

#### 5.1.5 Disorder of glucose lipid metabolism

Research indicates a link between HUA and glycolipid metabolism issues (83), highlighting the need for careful monitoring in high-risk groups to prevent HUA. HUA influences genes related to glucose and lipid metabolism, insulin signaling, and fat cell differentiation in mouse adipocytes (84). This can cause insulin resistance, cellular damage, and the onset of HUA and diabetes. Conversely, HUA can impact liver lipid metabolism by causing hepatic steatosis and disrupting fatty acid metabolism, leading to increased fatty acid synthesis, reduced oxidation, abnormal lipid metabolism, and insulin resistance. Consequently, compromised insulin sensitivity worsens diabetes progression. Both hyperglycemia and hyperlipidemia contribute to beta cell dysfunction in newly diagnosed patients with type 2 diabetes, and either condition can independently increase insulin resistance (85). In diabetes, high blood glucose from insulin resistance or inadequate insulin can increase liver gluconeogenesis and disrupt lipid metabolism, causing oxidative stress and inflammation, which may lead to HUA. Small molecule inhibitors targeting fatty acid synthase have been indicated to enhance liver function, reduce inflammation, and lower oxidative stress in obese mice on a high-sugar diet (86). Consequently, high glucose levels can increase fatty acid production, leading to their accumulation and impacting uric acid synthesis and excretion. Diabetic patients should manage blood sugar and lipids to lower HUA risk.

#### 5.1.6 Apoptosis

Research reveals that HUA levels entering rat islet beta cells through the organic anion transporter increase oxidative stress and activate mitogen-activated protein kinase signaling, hindering cell proliferation (87). High uric acid levels can cause insulin resistance in islet beta cells by raising ROS and disrupting the IRS2/AKT pathway (88). Furthermore, the PPAR-γ-Pck1-mTOR pathway might play a role in islet cell apoptosis due to high uric acid (89). HUA can cause insulin resistance in both lab and living organisms by inhibiting insulin receptor substrate activity through retinol-binding protein 4 (90). Consequently, HUA levels may lead to islet cell death, decreasing insulin production and possibly causing diabetes.

### 5.2 Promising drugs for HUA and diabetes

#### 5.2.1 SGLT-2 inhibitors

SGLT-2 inhibitors, kidney proteins that reabsorb glucose and sodium, have been demonstrated to be effective in reducing the need for HUA and gout treatment, according to a JAMA Cardiology study on daglizin (91). The Food and Drug Administration and European Medicines Agency have approved three oral SGLT2 regimens for type 2 diabetes and CKD patients with estimated glomerular filtration rate > 30 mL/min/1.72 m², enhancing health markers (92). SGLT2 inhibitors manage diabetes by preventing glucose reabsorption in the kidneys, thus lowering blood sugar. They may also reduce uric acid levels, particularly in diabetic patients with chronic conditions (93). These inhibitors boost glucose and ketone production, increase red blood cell count, lower uric acid, and reduce heart failure and kidney issues. By inhibiting SGLT2, they raise intracavicular glucose, which competes with uric acid reabsorption u GLUT9b (94). SGLT2 inhibitors may help lower anti-inflammatory, antioxidant, and antifibrotic markers (95). They reduce blood uric acid by promoting urinary D-glucose excretion, altering renal tubule uric acid transport (96). Additionally, they improve endothelial function and decrease inflammation in the liver and fat tissue (97). Their studies indicate that SGLT-2 inhibitors can reduce inflammation, alleviate symptoms of HUA and diabetes, and lower uric acid levels by improving endothelial function and suppressing inflammation. They enhance glucose and uric acid excretion, reducing uric acid buildup in the blood. This mechanism involves an increase in glomerular filtration rate due to SGLT-2 inhibitors and reduced uric acid reabsorption in the collecting ducts through GLUT9, leading to lower blood sugar and uric acid levels by replacing glucose reabsorption (98). A meta-analysis found that SGLT-2 inhibitors promote fat oxidation and weight loss, potentially improving HUA and diabetes symptoms (99). Overall, SGLT-2 inhibitors effectively reduce blood glucose levels, decrease uric acid concentrations, lower blood pressure, and facilitate weight loss, thereby potentially contributing to a reduction in uric acid levels in patients.

#### 5.2.2 Glucagon-like peptide-1 receptor agonists

GLP-1RA effectively manages HUA in diabetic patients by reducing appetite and promoting weight loss. It modulates the vagal nerve pathway to decrease food intake, leading to lower blood sugar and uric acid levels (100). Recent studies have found that AWRK6 is a new GLP-1 receptor agonist that boosts insulin secretion to control blood glucose and energy metabolism using the cAMP-calcium pathway, with minimal toxicity (101). Accordingly, AWRK6 indicates promise as a diabetes treatment. Pechenov et al. (102) revealed that combining sodium deoxycholic acid and propyl gallate in MEDI7219 tablets effectively reduces food intake, body weight, and glucose levels in obese, insulin-resistant dogs, suggesting potential for new oral peptide therapies. The study also found that short-acting GLP-1 receptor agonists such as exenatide and lixisenatide can slow gastric emptying, lower blood glucose, and reduce uric acid production (103).

Additionally, liraglutide and semaglutide cause more gastrointestinal side effects than dulaglutide. Higher doses of GLP-1RA increase this risk, regardless of drug type or formulation, possibly due to appetite center inhibition (104). GLP-1RA drugs enhance insulin sensitivity and reduce blood sugar and uric acid in diabetic patients with HUA (103). Recent studies suggest that reducing oxidative stress can improve blood glucose issues and help repair damaged blood vessels (105). GLP-1RA may lower inflammation and oxidative stress by enhancing glucose control, reducing obesity, and improving insulin resistance and metabolic function in people with diabetes and HUA. It is speculated that GLP-1RA may reduce inflammation and oxidative stress by improving glucose control and reducing obesity, as well as improving insulin resistance and metabolic function in individuals with diabetes and HUA. Overall, GLP-1RA effectively manages diabetes with HUA.

#### 5.2.3 Febuxostat

Febuxostat, a new non-purine xanthine oxidase inhibitor, has gained attention for its effectiveness in lowering serum uric acid and potentially improving diabetes-related complications in patients with hyperuricemic diabetes. Initially, febuxostat’s anti-inflammatory properties could benefit people with diabetes by reducing HUA-related inflammation, potentially improving their overall health (106). Subsequently, febuxostat might enhance cardiovascular health in diabetic patients by improving endothelial function (107). However, its limited impact on endothelial function over long-term treatment, despite effectively lowering uric acid, indicates a need for further investigation. Moreover, febuxostat’s antioxidant properties may aid in treating diabetes with HUA by reducing superoxide anion production and oxidative stress, which is linked to insulin resistance (108). This could indirectly enhance insulin sensitivity. Febuxostat may strengthen kidney function by significantly lowering uric acid levels in kidney transplant patients, offering short-term improvements (109). This is crucial for people with diabetes, as managing uric acid can help delay diabetic kidney disease progression.

In summary, there are safety concerns regarding febuxostat’s use in diabetic patients, as it may increase cardiovascular risk, necessitating careful evaluation (108, 111). Besides, febuxostat could be crucial in treating diabetes with HUA by reducing uric acid, enhancing insulin sensitivity, reducing inflammation, and protecting kidney function. However, more research is needed to verify its long-term effects and safety in diabetic patients.

## 6 Advantages and disadvantages

This study applied scientometrics to analyze the relationship between HUA and diabetes, revealing current interests and future trends while providing a theoretical foundation for further research. However, it is limited to literature from the Web of Science Core Collection, excluding non-English studies and not addressing the relationship between HUA and different diabetes types.

## 7 Conclusion

Current research trends reveal that studying HUA and diabetes is a dynamic field with many opportunities. By examining their correlation, we can identify individuals at high risk for early-stage diabetes, offering a theoretical basis for interventions to prevent its progression. Consequently, comprehensive treatment plans for individuals with HUA and diabetes are crucial to manage their conditions and prevent complications. Patients with persistent HUA should be prioritized in diabetes prevention guidelines.

## Data Availability

The original contributions presented in the study are included in the article/**Supplementary Material**. Further inquiries can be directed to the corresponding author.
