# Supplementary material for: The link between hyperuricemia and diabetes: insights from a quantitative analysis of scientific literature
Source: Front Endocrinol (Lausanne). 2025 Feb 7;15:1441503. doi: 10.3389/fendo.2024.1441503 (PMC11842261; doi:10.3389/fendo.2024.1441503)
Supplement: Supplementary file 1 [file Table1.doc]

| Number | Author | Documents | Citations | Total link strength |
| --- | --- | --- | --- | --- |
| 1 | johnson, richard j. | 41 | 3558 | 125 |
| 2 | lanaspa, miguel a. | 19 | 1179 | 110 |
| 3 | kuwabara, masanari | 18 | 752 | 98 |
| 4 | hisatome, ichiro | 15 | 795 | 91 |
| 5 | niwa, koichiro | 12 | 686 | 89 |
| 6 | bjornstad, petter | 11 | 514 | 65 |
| 7 | kanbay, mehmet | 11 | 322 | 30 |
| 8 | krishnan, eswar | 11 | 1047 | 0 |
| 9 | andres-hernando, ana | 10 | 606 | 82 |
| 10 | li, changgui | 9 | 623 | 6 |

Table 1 Basic information of the top 10 authors
