# Supplementary material for: The link between hyperuricemia and diabetes: insights from a quantitative analysis of scientific literature
Source: Front Endocrinol (Lausanne). 2025 Feb 7;15:1441503. doi: 10.3389/fendo.2024.1441503 (PMC11842261; doi:10.3389/fendo.2024.1441503)
Supplement: Supplementary file 2 [file Table2.doc]

Table 2 Basic information of the top 10 countries in terms of publication volume

| Number | Country | Documents | Citations | Total link strength |
| --- | --- | --- | --- | --- |
| 1 | peoples r china | 433 | 5973 | 83 |
| 2 | usa | 259 | 21438 | 207 |
| 3 | japan | 202 | 5389 | 39 |
| 4 | taiwan | 103 | 3092 | 14 |
| 5 | italy | 100 | 4369 | 62 |
| 6 | south korea | 49 | 1717 | 8 |
| 7 | england | 46 | 3121 | 82 |
| 8 | mexico | 40 | 1061 | 30 |
| 9 | turkey | 40 | 1005 | 22 |
| 10 | germany | 39 | 1681 | 56 |
