# Supplementary material for: The link between hyperuricemia and diabetes: insights from a quantitative analysis of scientific literature
Source: Front Endocrinol (Lausanne). 2025 Feb 7;15:1441503. doi: 10.3389/fendo.2024.1441503 (PMC11842261; doi:10.3389/fendo.2024.1441503)
Supplement: Supplementary file 3 [file Table3.doc]

Table 3 Basic information of the top 10 institutions with the number of publications

| Number | organization | documents | citations | total link strength |
| --- | --- | --- | --- | --- |
| 1 | china med univ | 36 | 469 | 69 |
| 2 | shanghai jiao tong univ | 33 | 501 | 13 |
| 3 | capital med univ | 31 | 216 | 30 |
| 4 | univ colorado | 30 | 2225 | 46 |
| 5 | qingdao univ | 22 | 827 | 19 |
| 6 | fudan univ | 21 | 144 | 7 |
| 7 | peking univ | 21 | 550 | 30 |
| 8 | toranomon gen hosp | 21 | 780 | 75 |
| 9 | nagoya univ | 18 | 1467 | 37 |
| 10 | sichuan univ | 18 | 141 | 9 |
