# Supplementary figures and images for: The link between hyperuricemia and diabetes: insights from a quantitative analysis of scientific literature
Source: Front Endocrinol (Lausanne). 2025 Feb 7;15:1441503. doi: 10.3389/fendo.2024.1441503 (PMC11842261; doi:10.3389/fendo.2024.1441503)

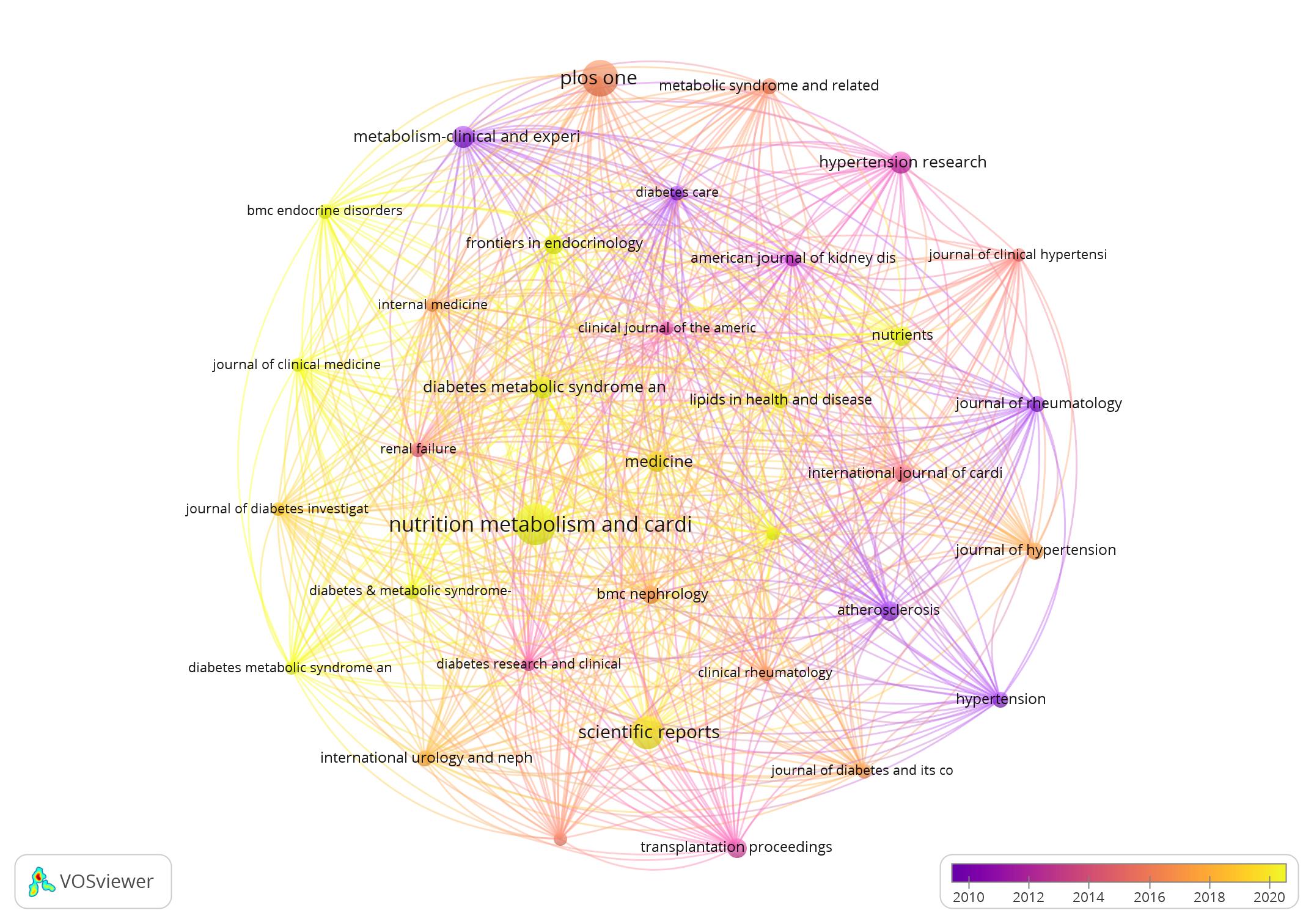

Supplement: Supplementary file 4 [file Image1.jpeg]

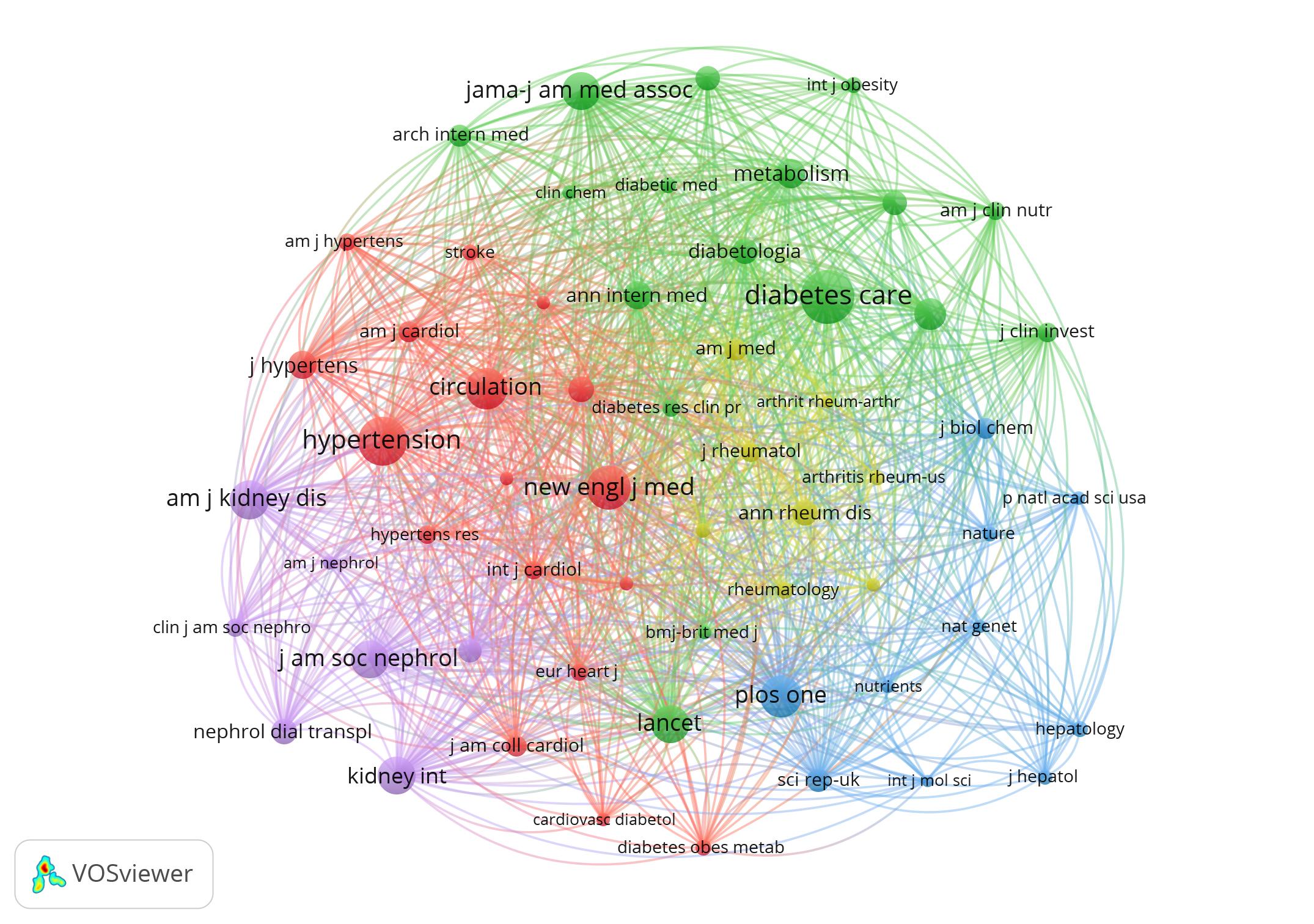

Supplement: Supplementary file 5 [file Image2.jpeg]

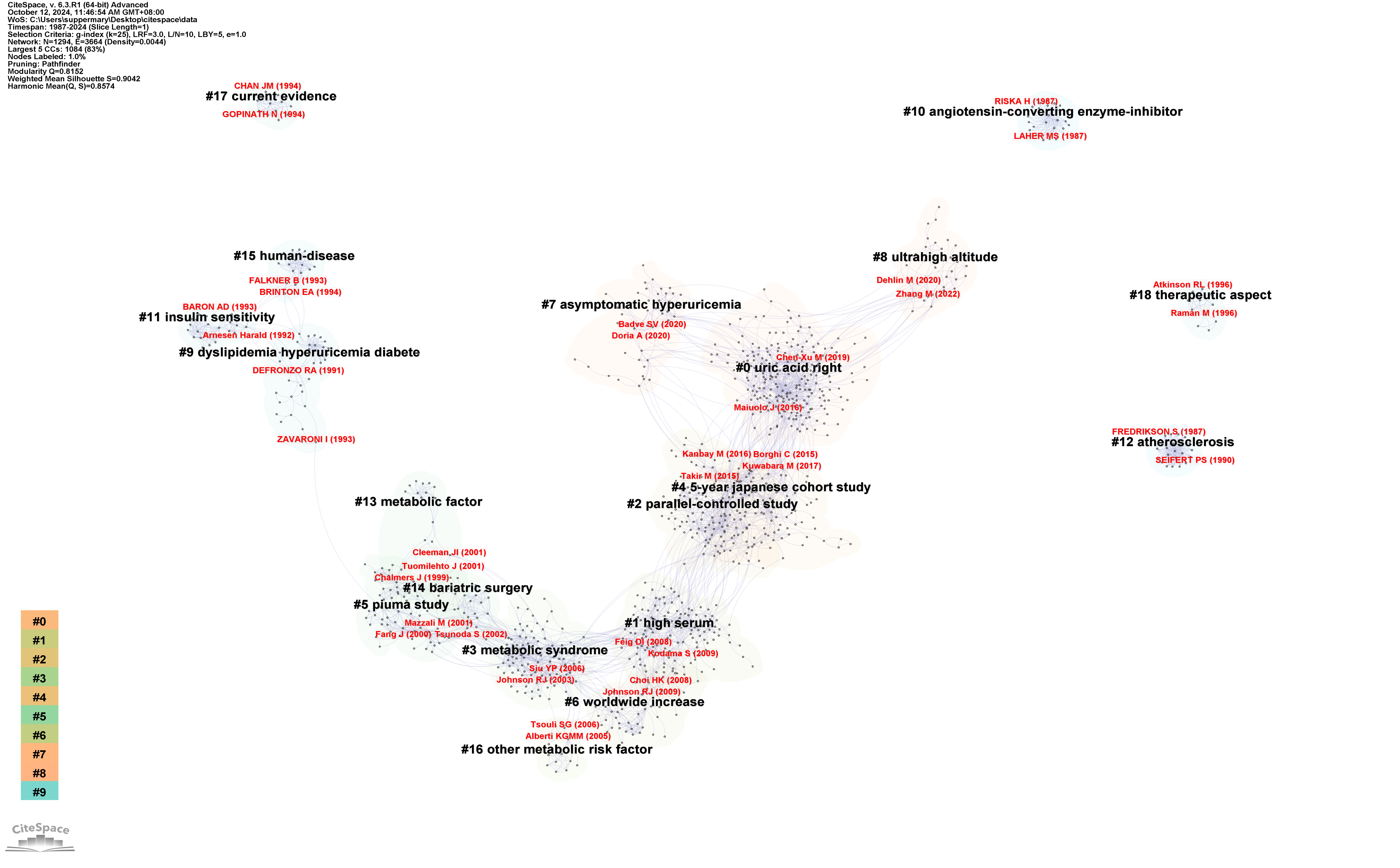

Supplement: Supplementary file 6 [file Supplementaryfile1.zip › Supplementary material Annex 1/1987.png]

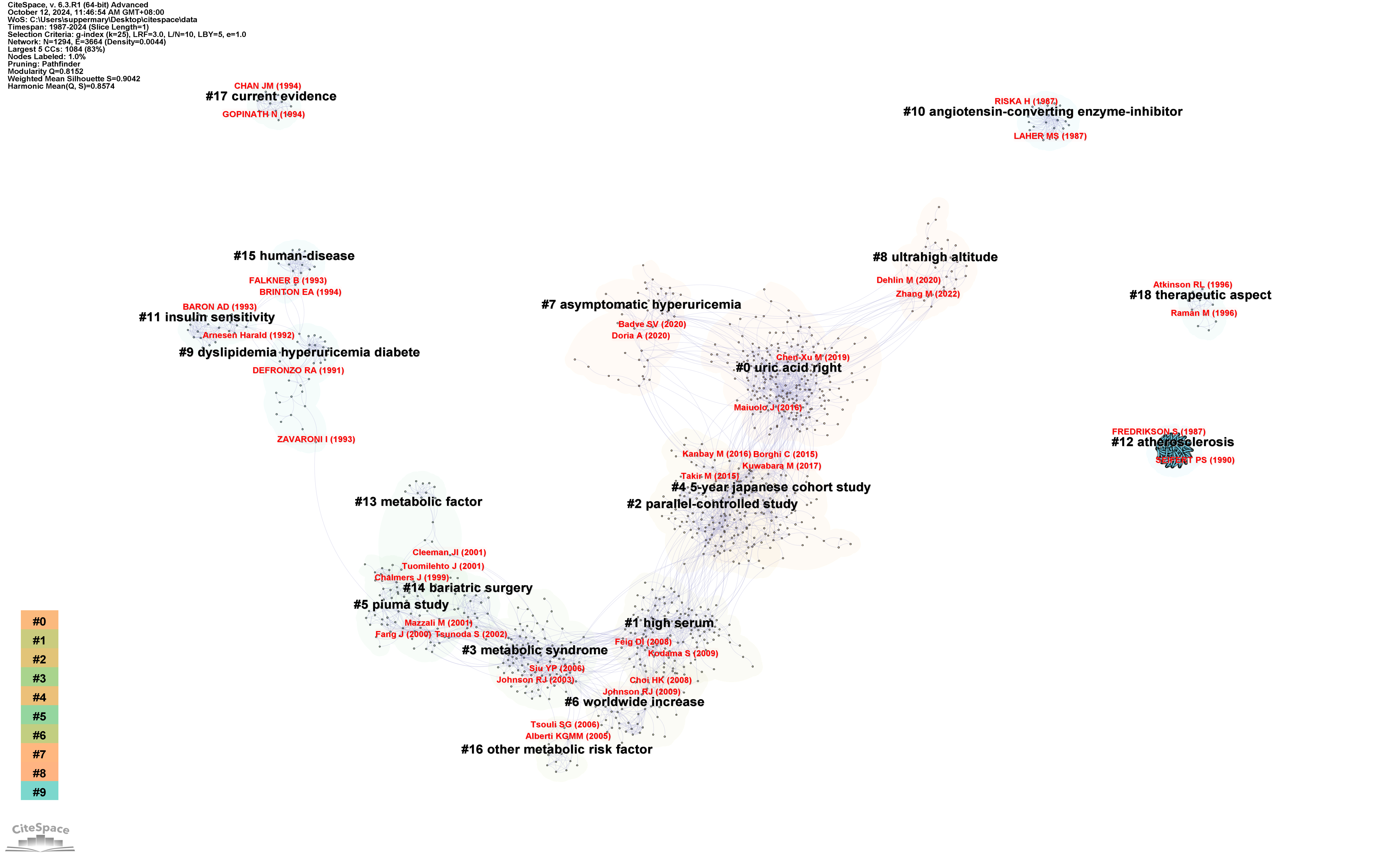

Supplement: Supplementary file 6 [file Supplementaryfile1.zip › Supplementary material Annex 1/1991.png]

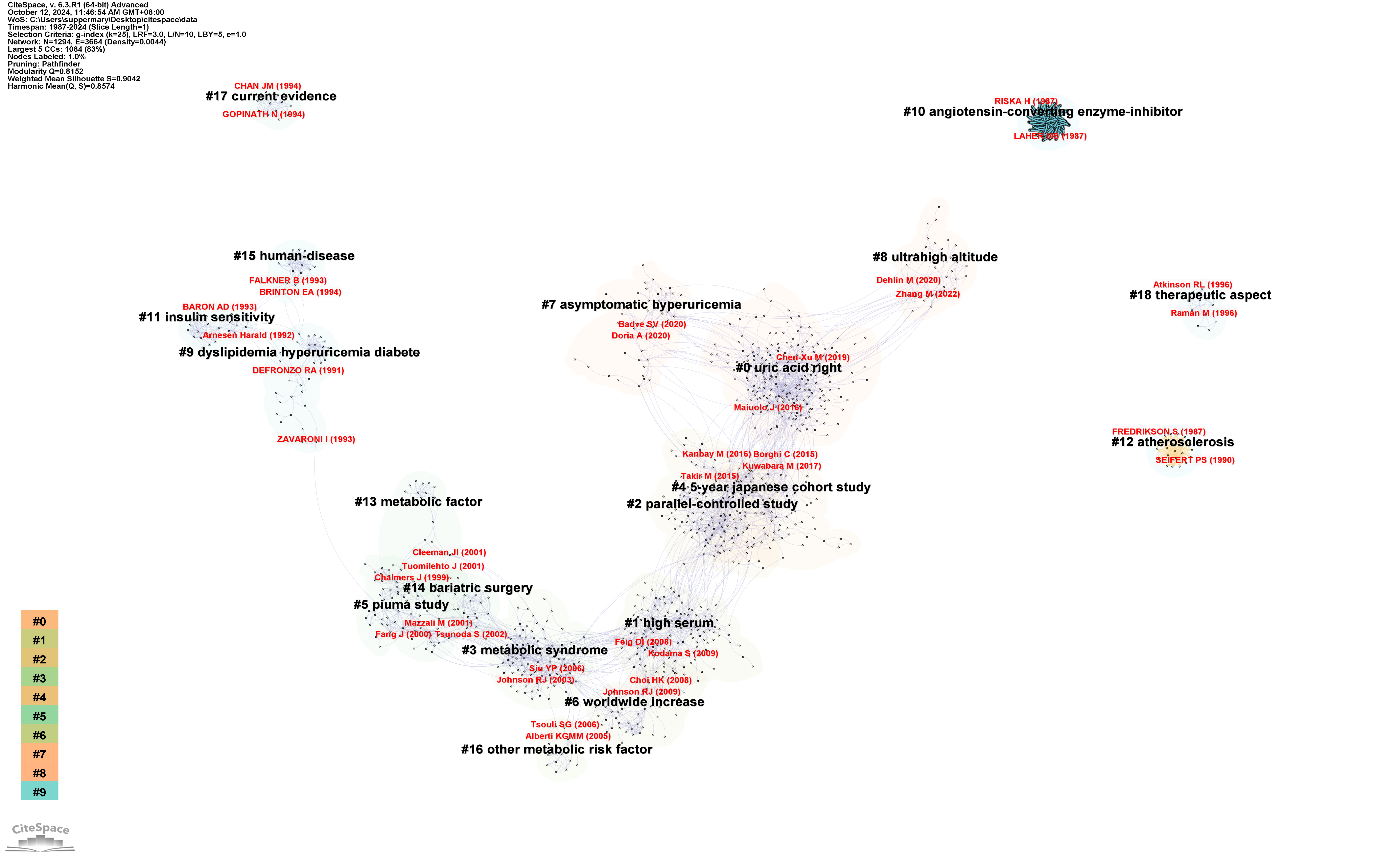

Supplement: Supplementary file 6 [file Supplementaryfile1.zip › Supplementary material Annex 1/1992.png]

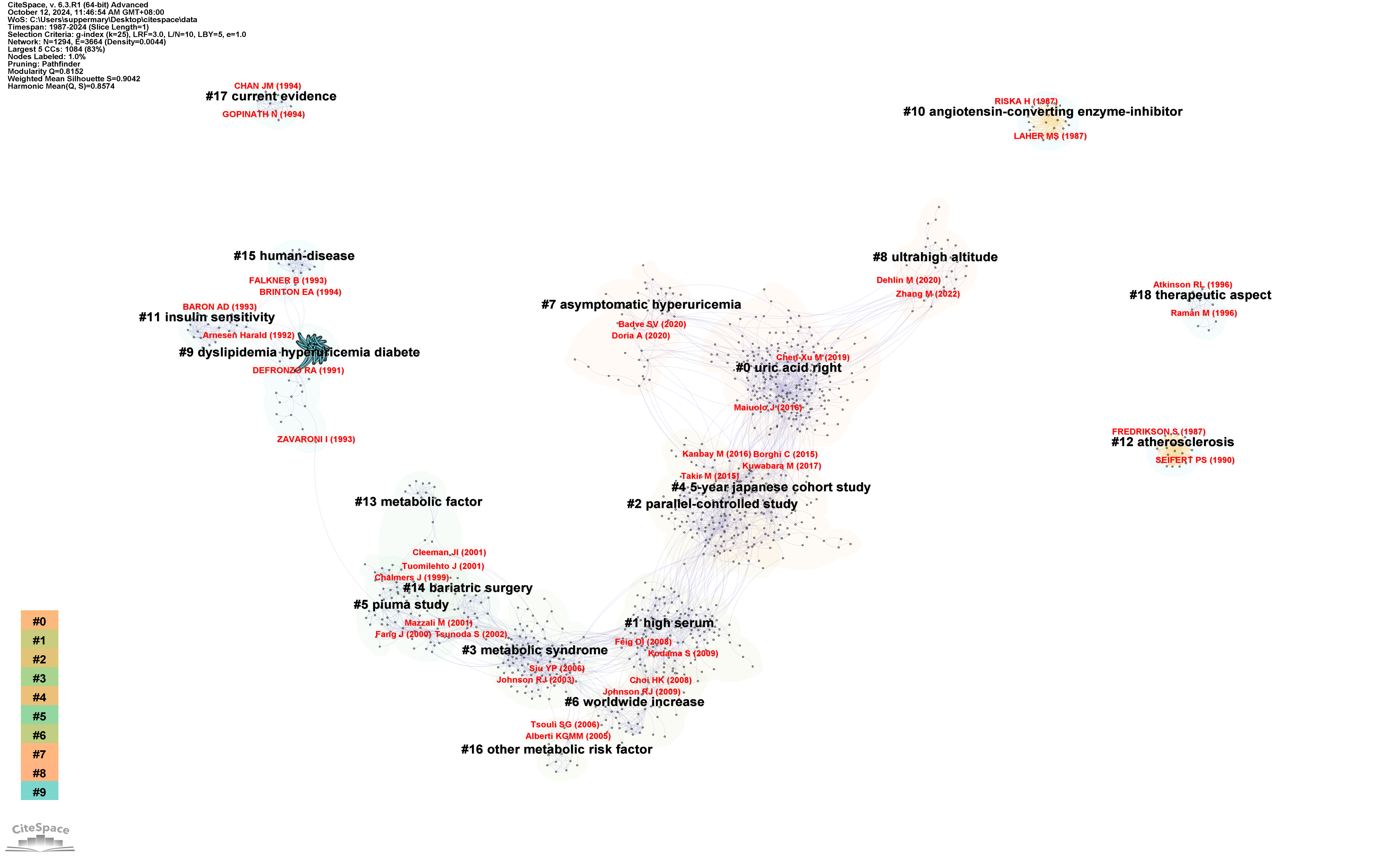

Supplement: Supplementary file 6 [file Supplementaryfile1.zip › Supplementary material Annex 1/1993.png]

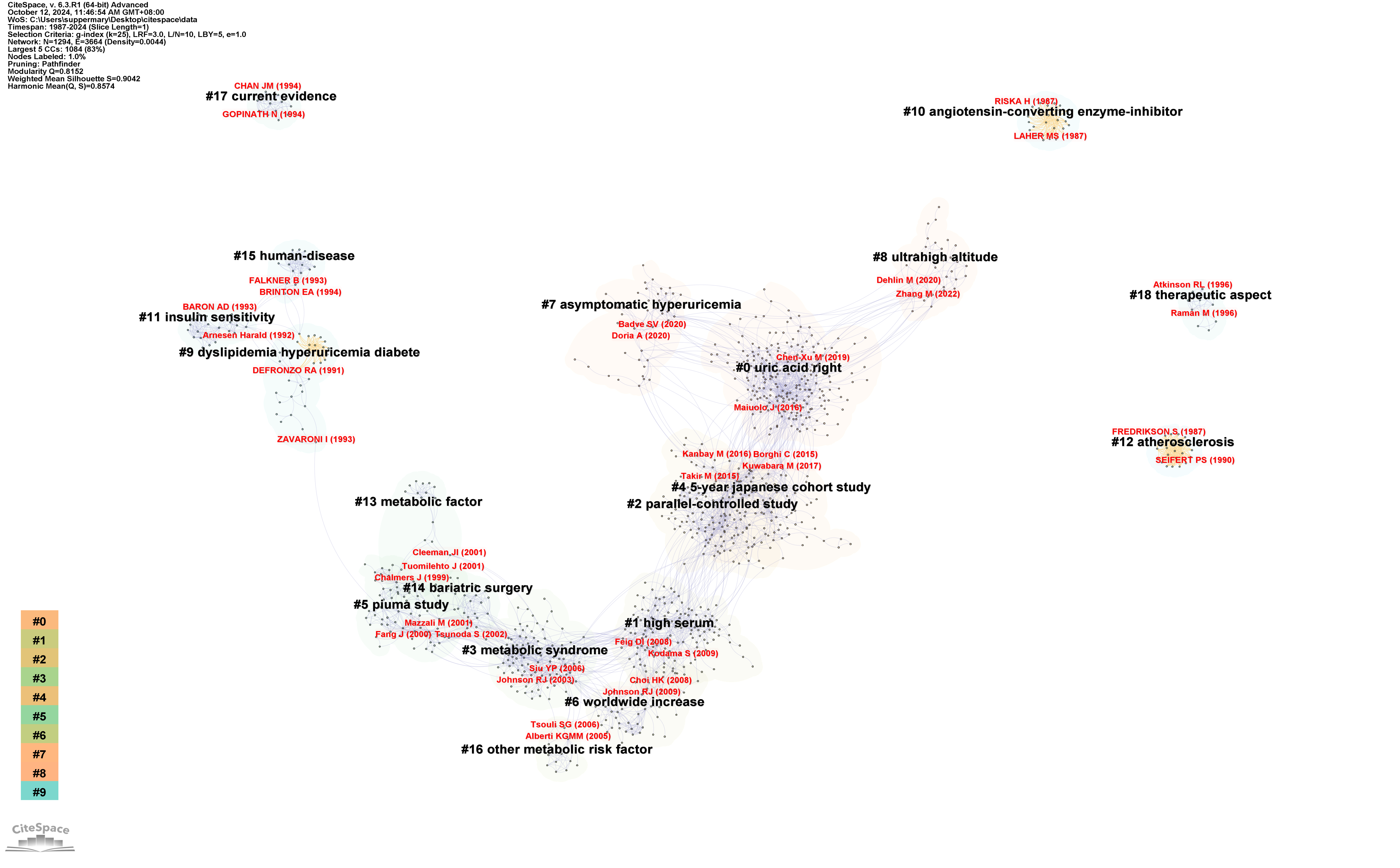

Supplement: Supplementary file 6 [file Supplementaryfile1.zip › Supplementary material Annex 1/1994.png]

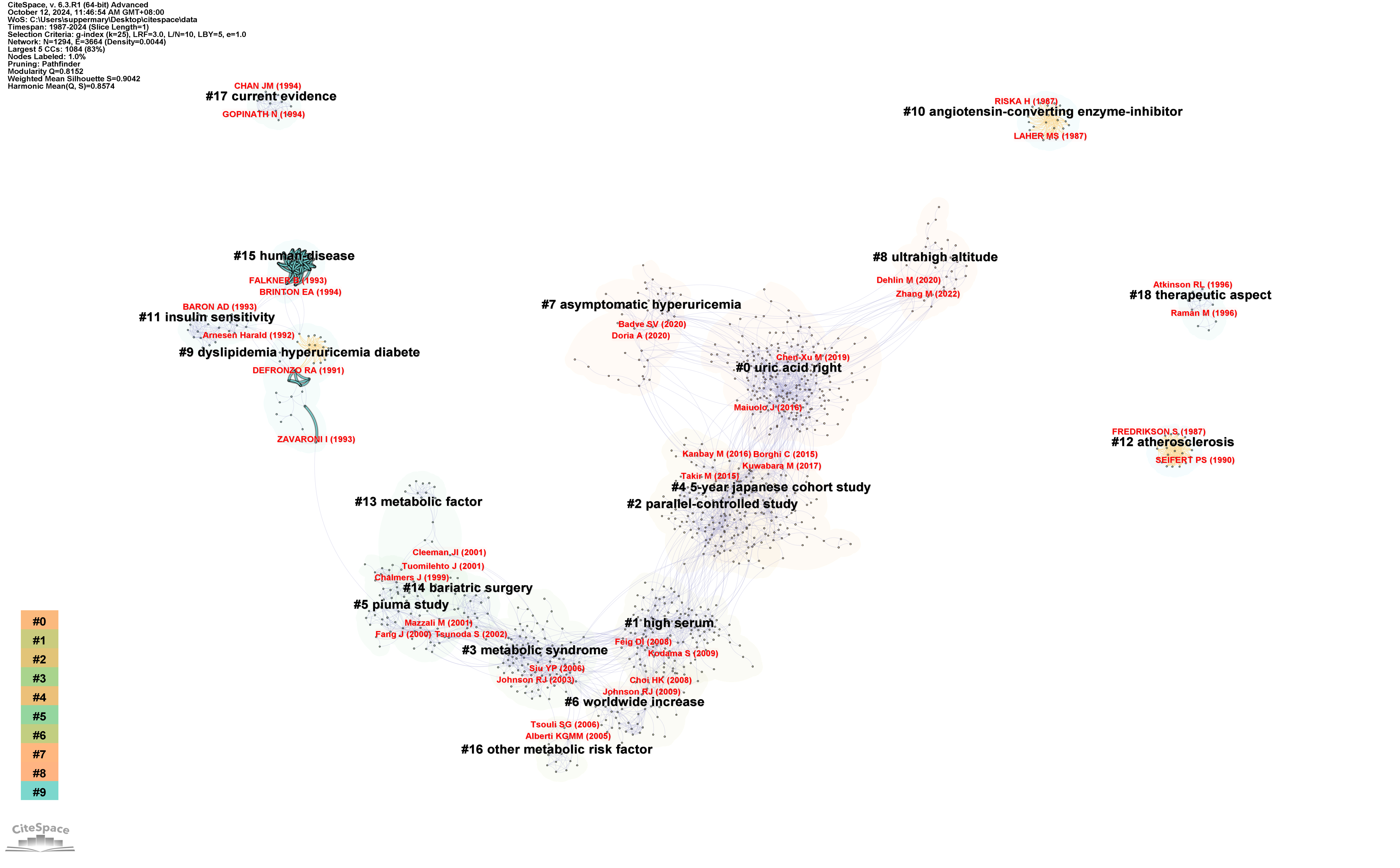

Supplement: Supplementary file 6 [file Supplementaryfile1.zip › Supplementary material Annex 1/1995.png]

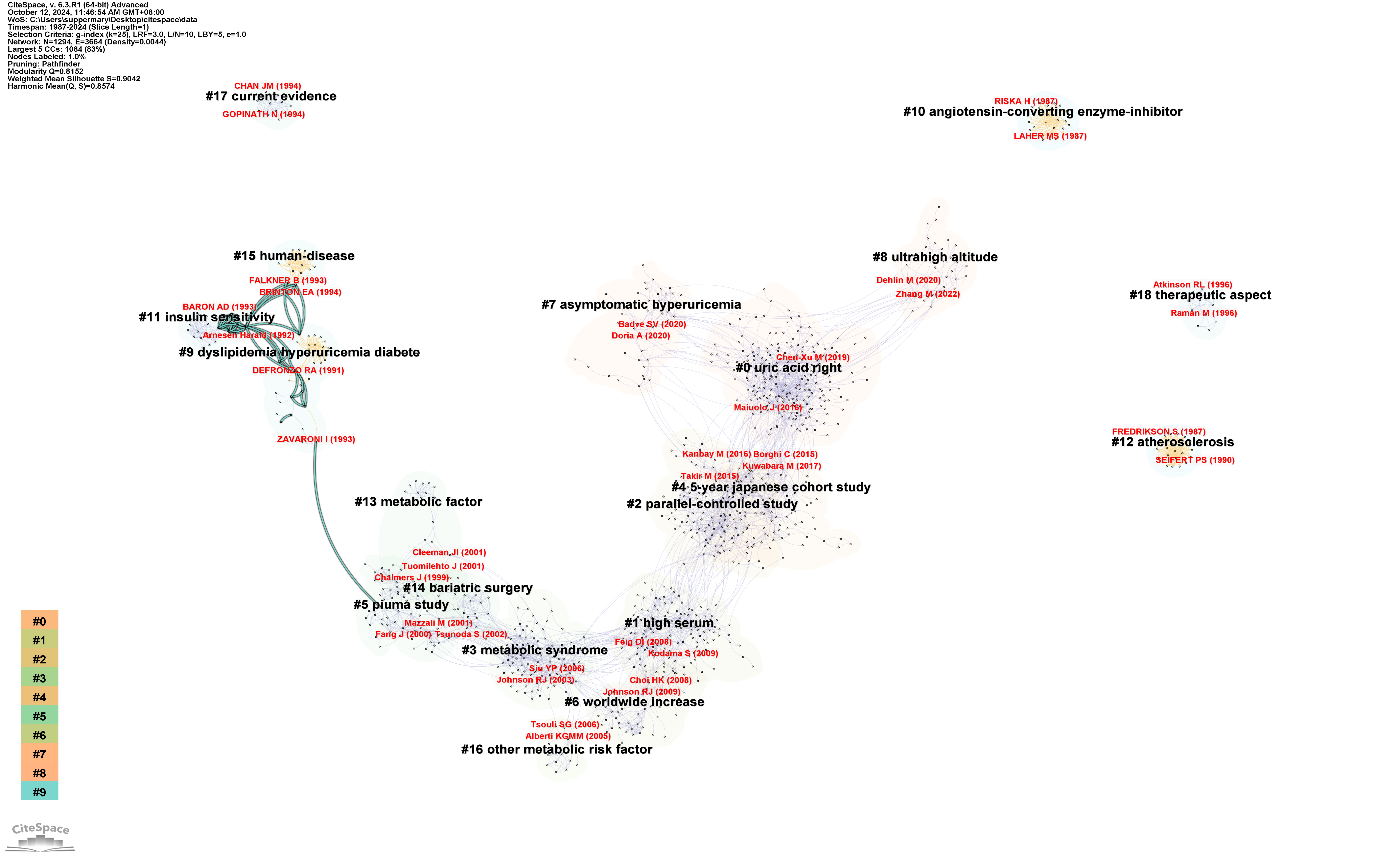

Supplement: Supplementary file 6 [file Supplementaryfile1.zip › Supplementary material Annex 1/1996.png]

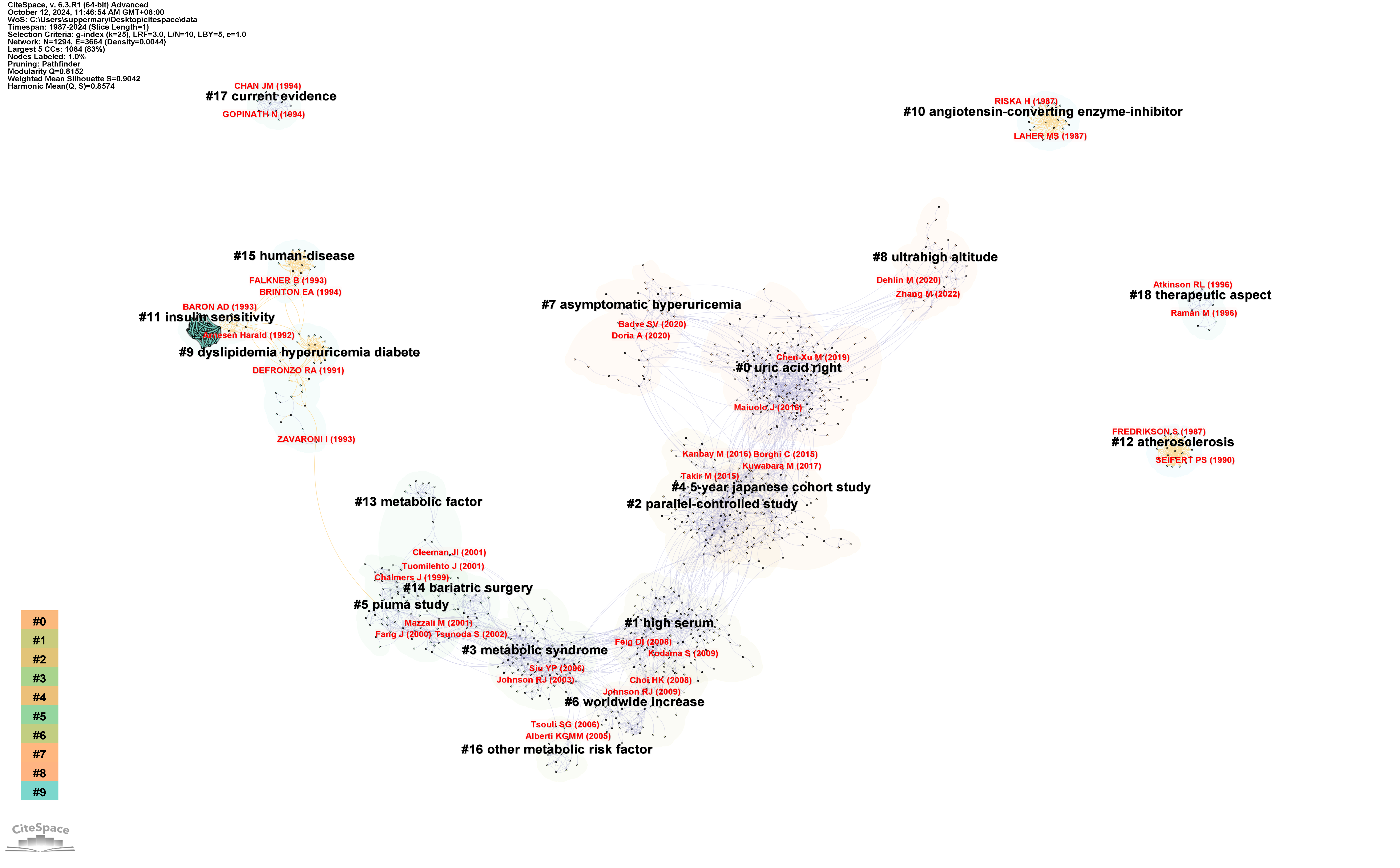

Supplement: Supplementary file 6 [file Supplementaryfile1.zip › Supplementary material Annex 1/1997.png]

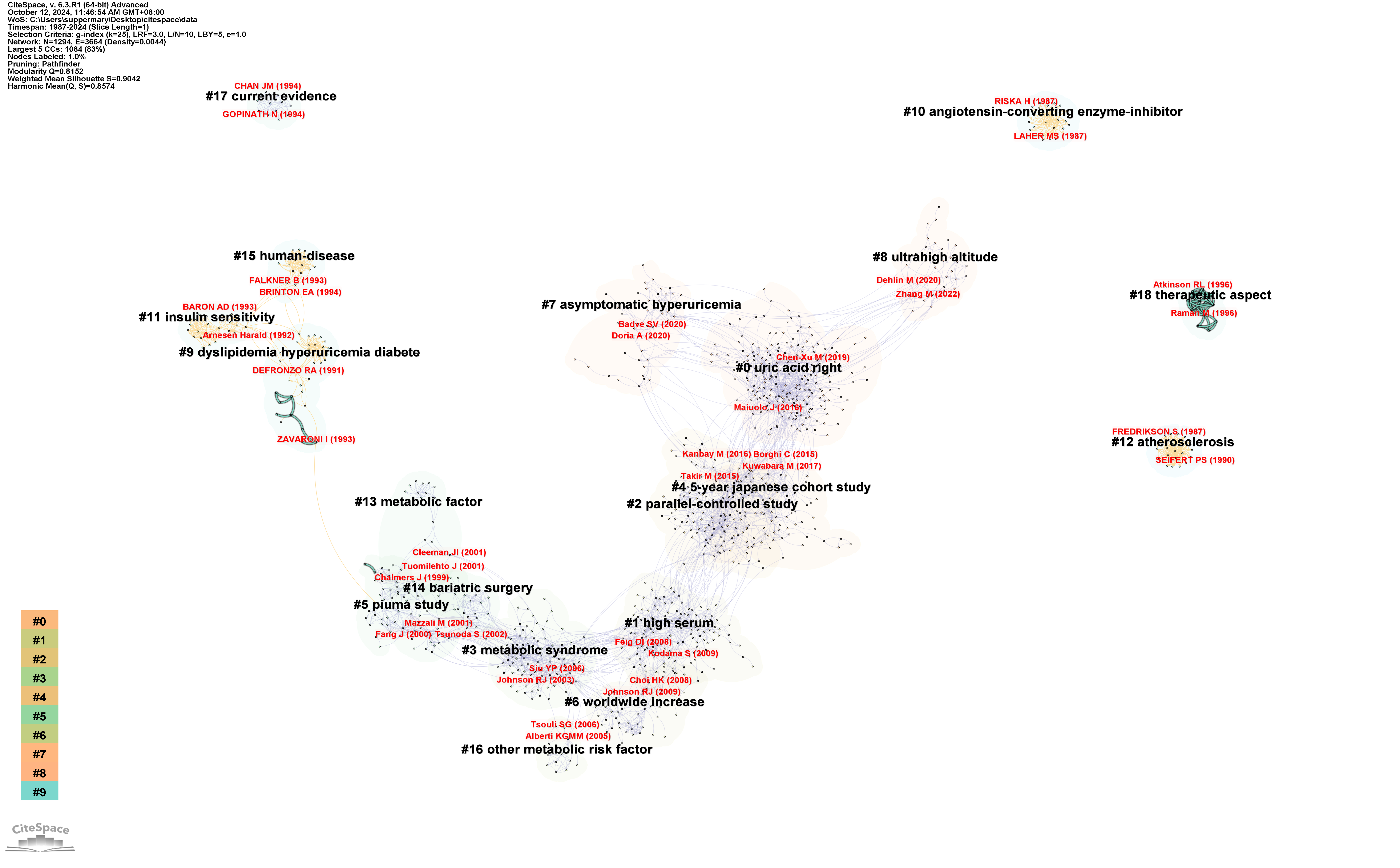

Supplement: Supplementary file 6 [file Supplementaryfile1.zip › Supplementary material Annex 1/1998.png]

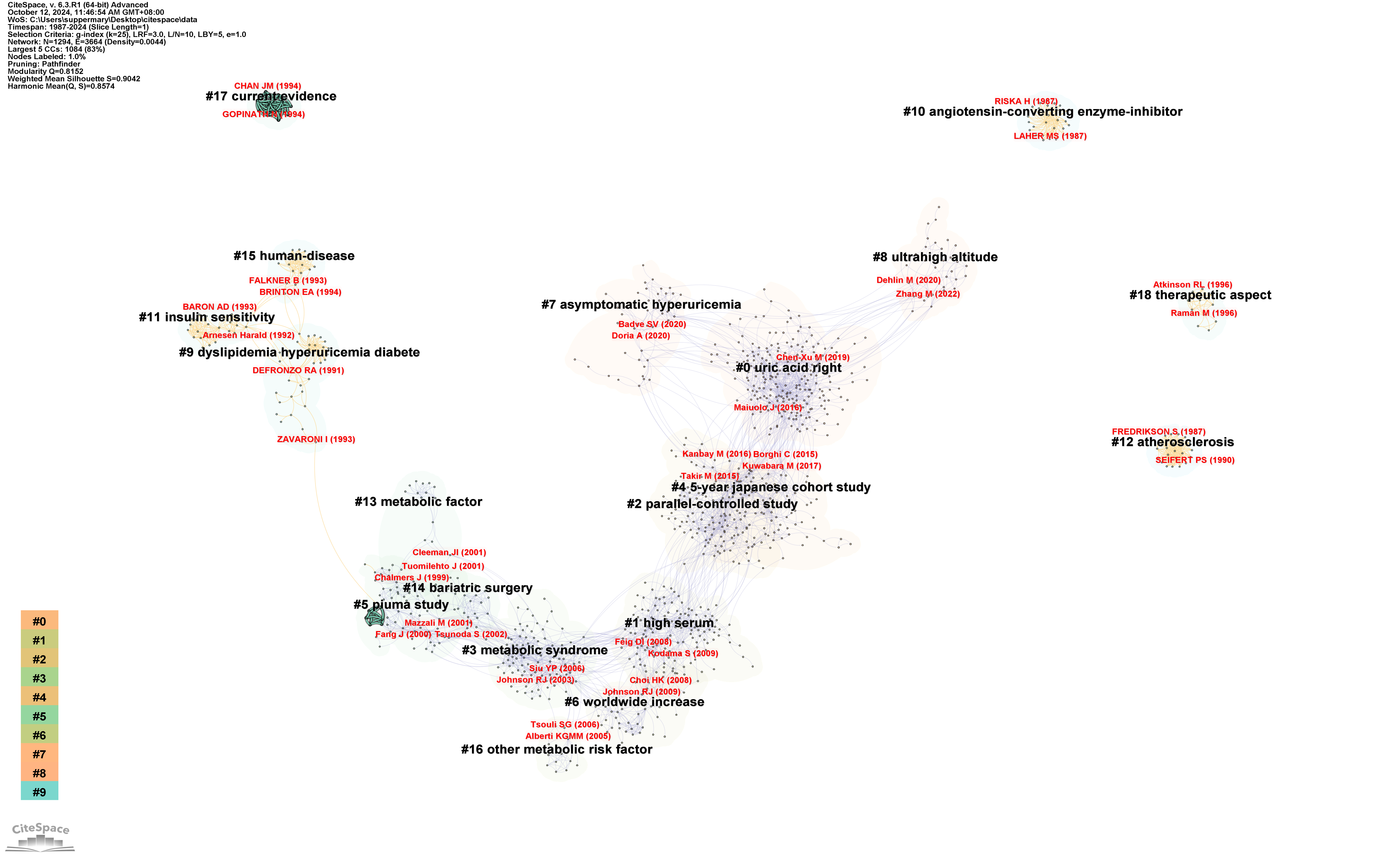

Supplement: Supplementary file 6 [file Supplementaryfile1.zip › Supplementary material Annex 1/1999.png]

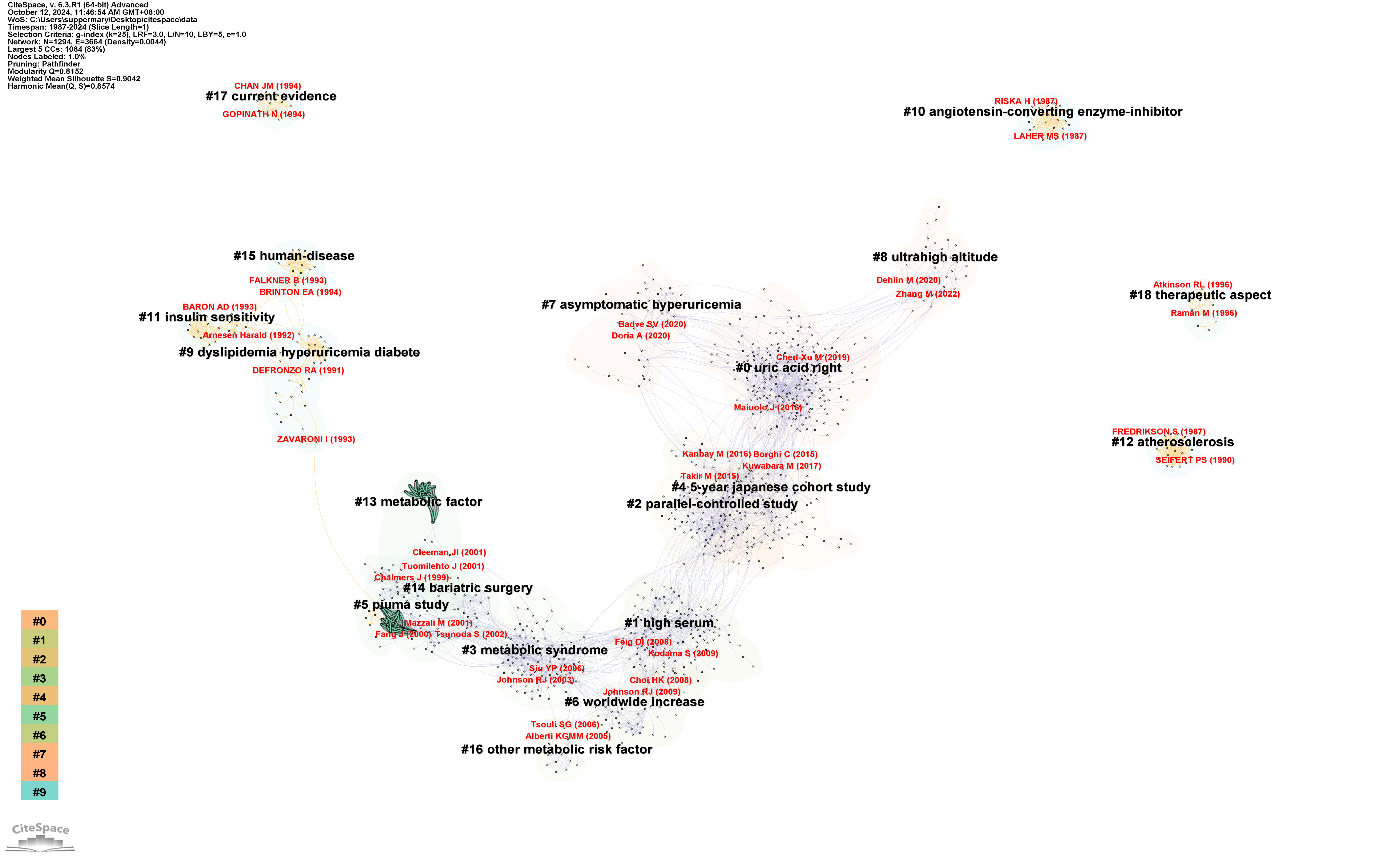

Supplement: Supplementary file 6 [file Supplementaryfile1.zip › Supplementary material Annex 1/2000.png]

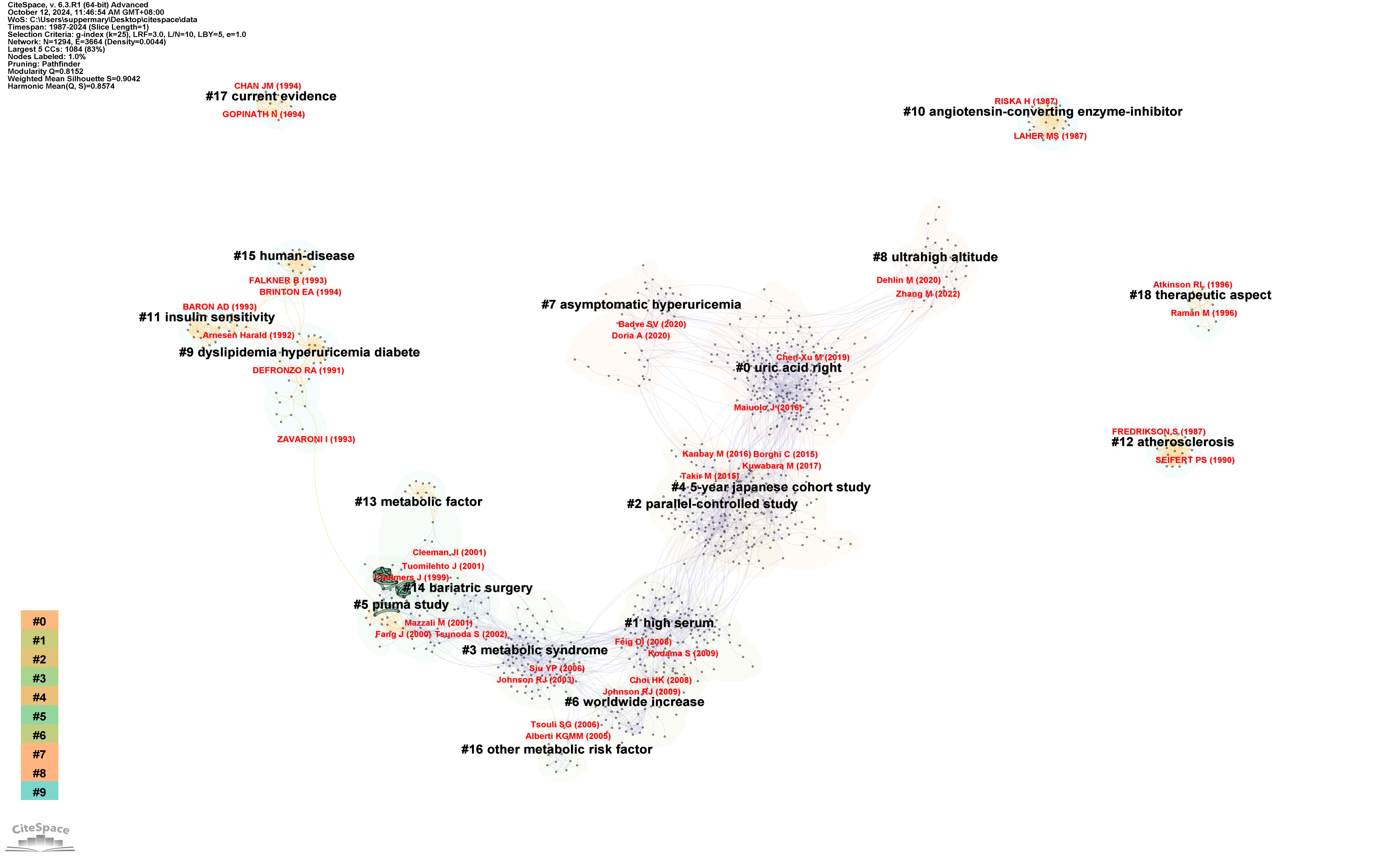

Supplement: Supplementary file 6 [file Supplementaryfile1.zip › Supplementary material Annex 1/2001.png]

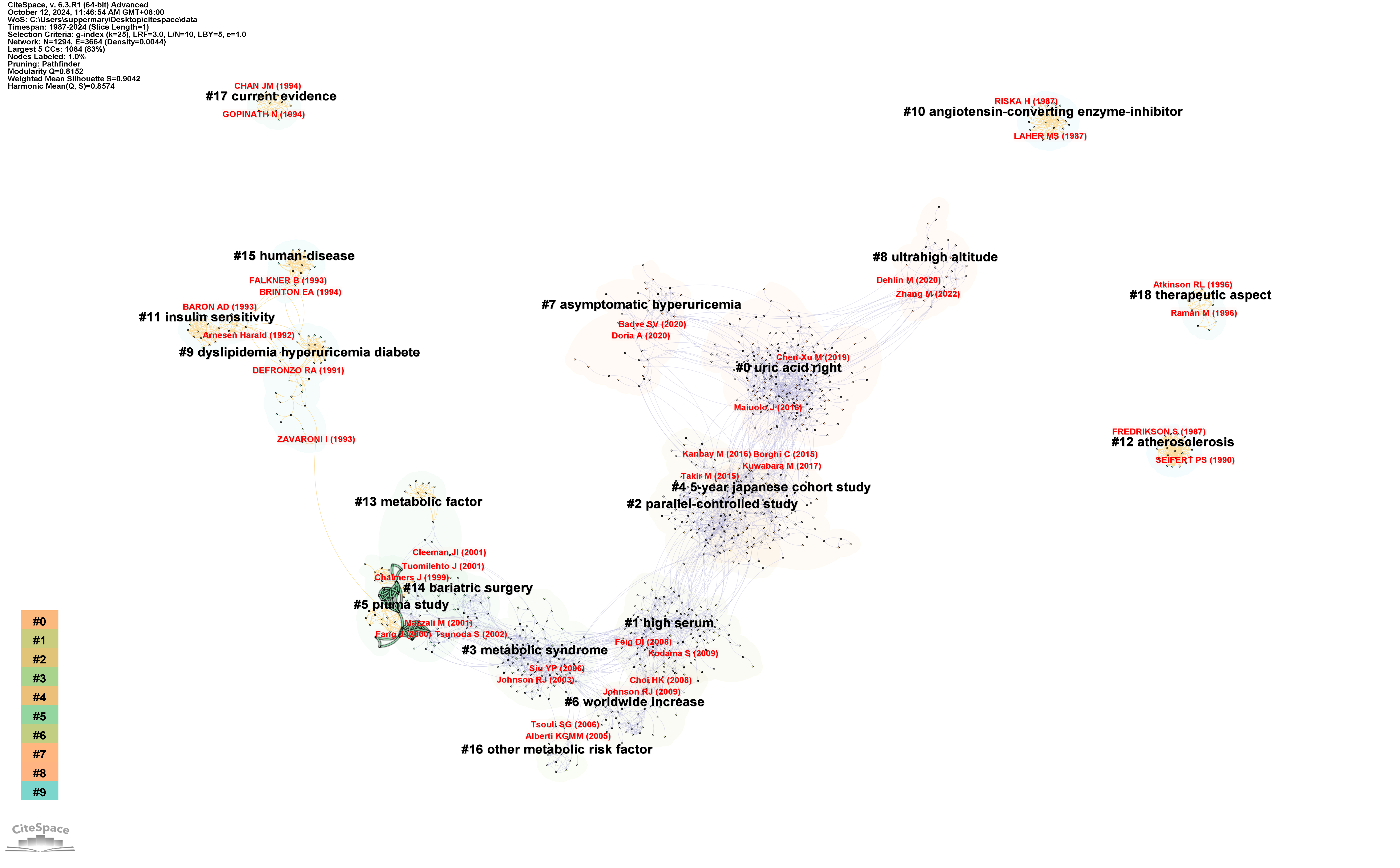

Supplement: Supplementary file 6 [file Supplementaryfile1.zip › Supplementary material Annex 1/2002.png]

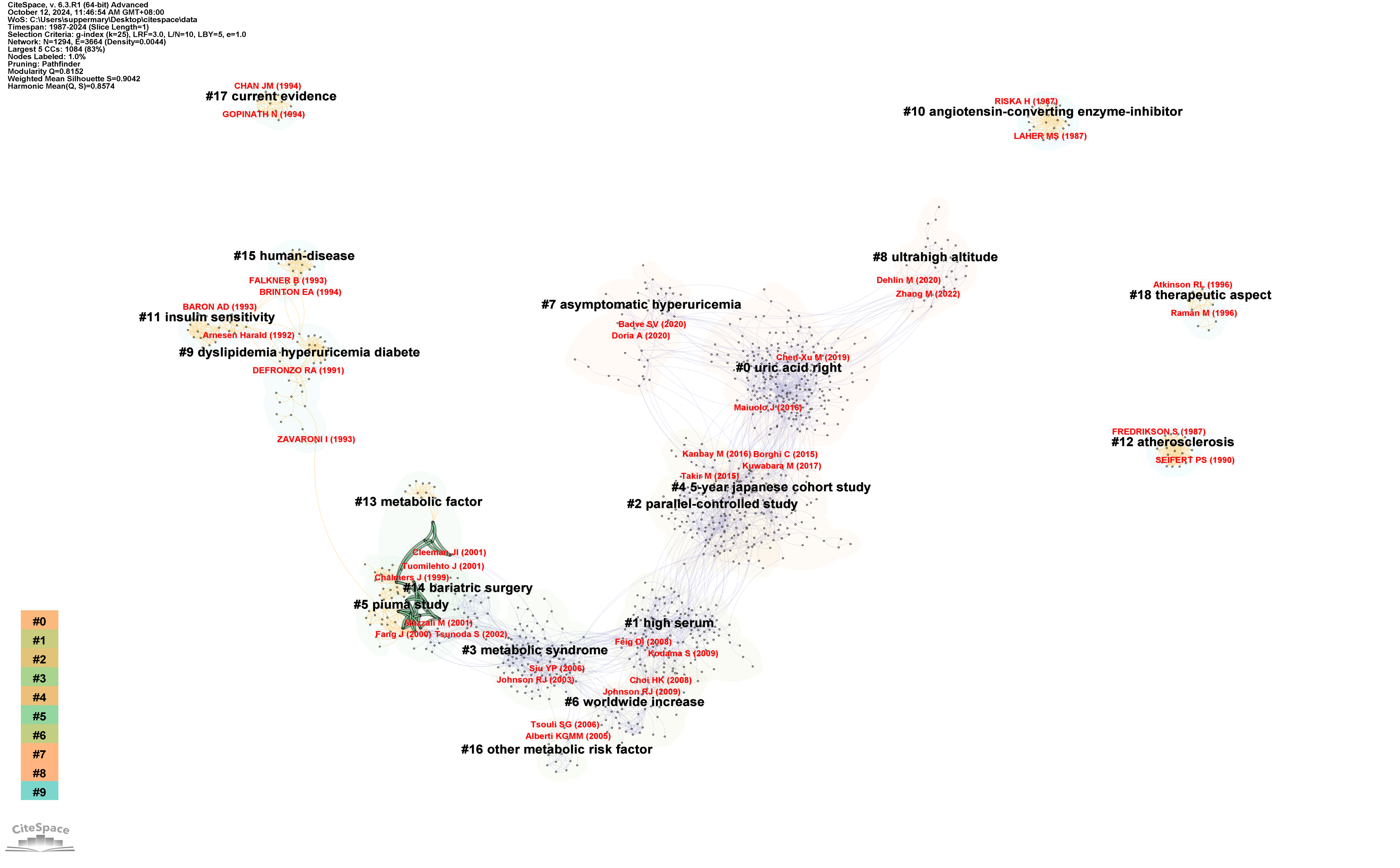

Supplement: Supplementary file 6 [file Supplementaryfile1.zip › Supplementary material Annex 1/2003.png]

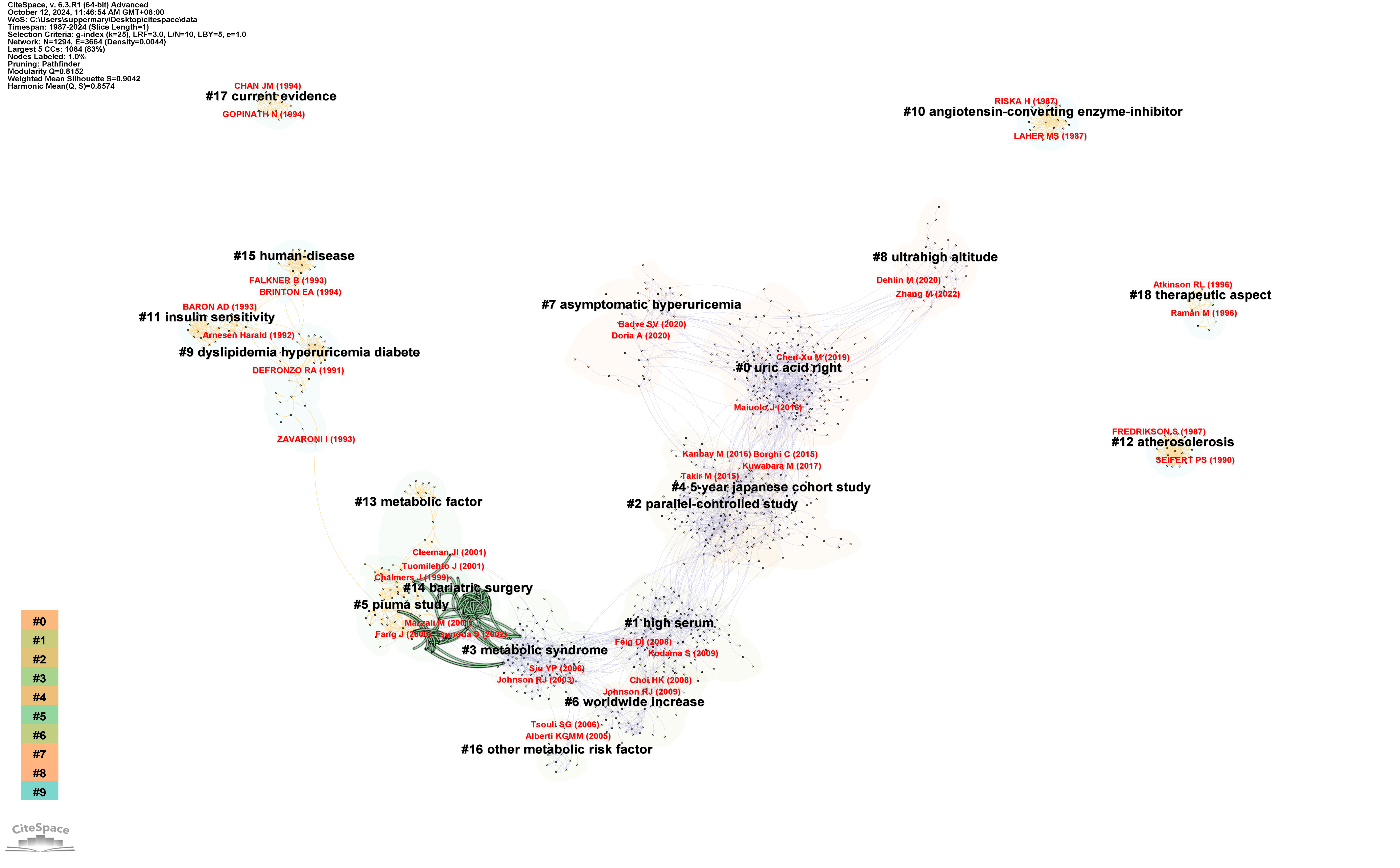

Supplement: Supplementary file 6 [file Supplementaryfile1.zip › Supplementary material Annex 1/2004.png]

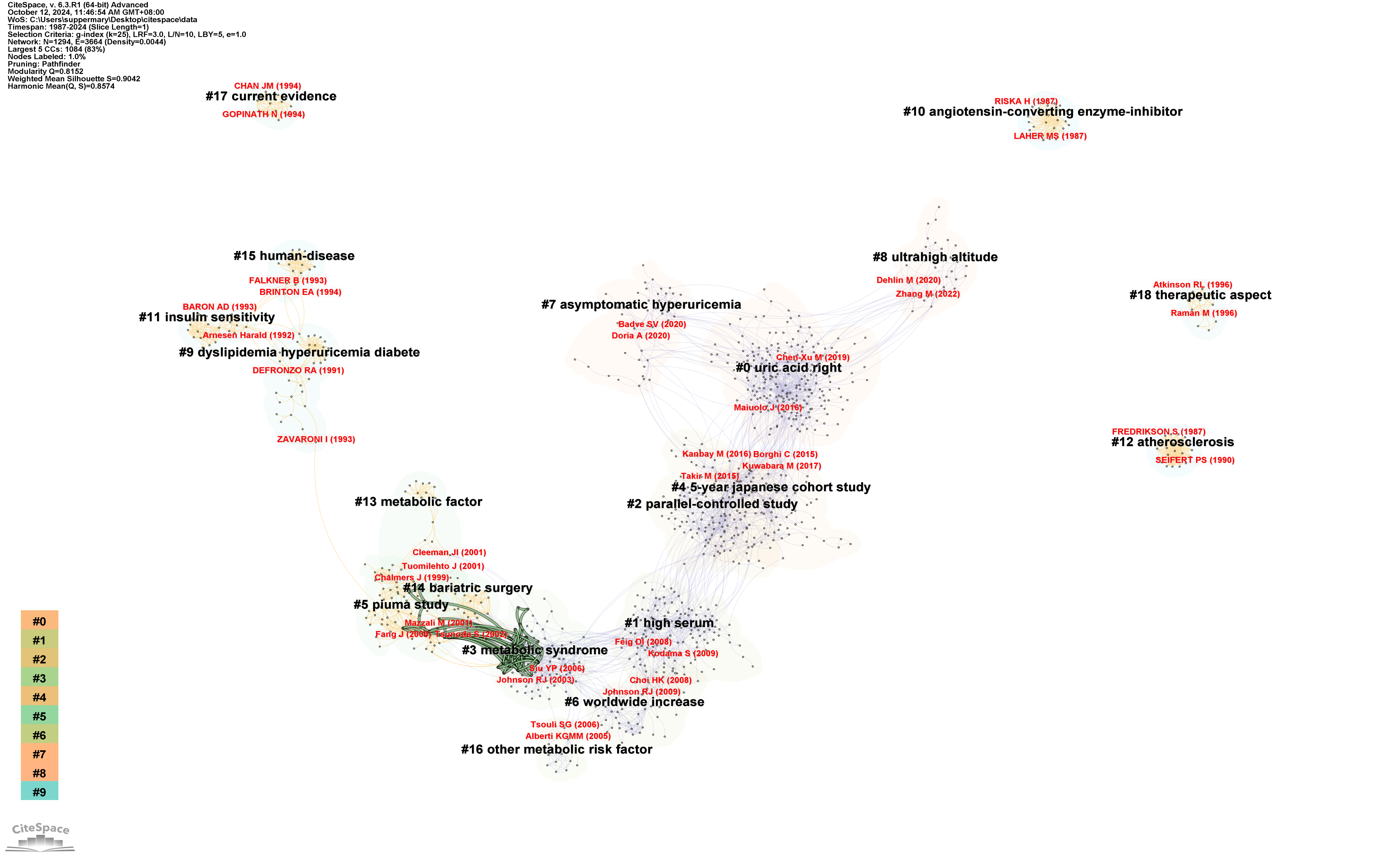

Supplement: Supplementary file 6 [file Supplementaryfile1.zip › Supplementary material Annex 1/2005.png]

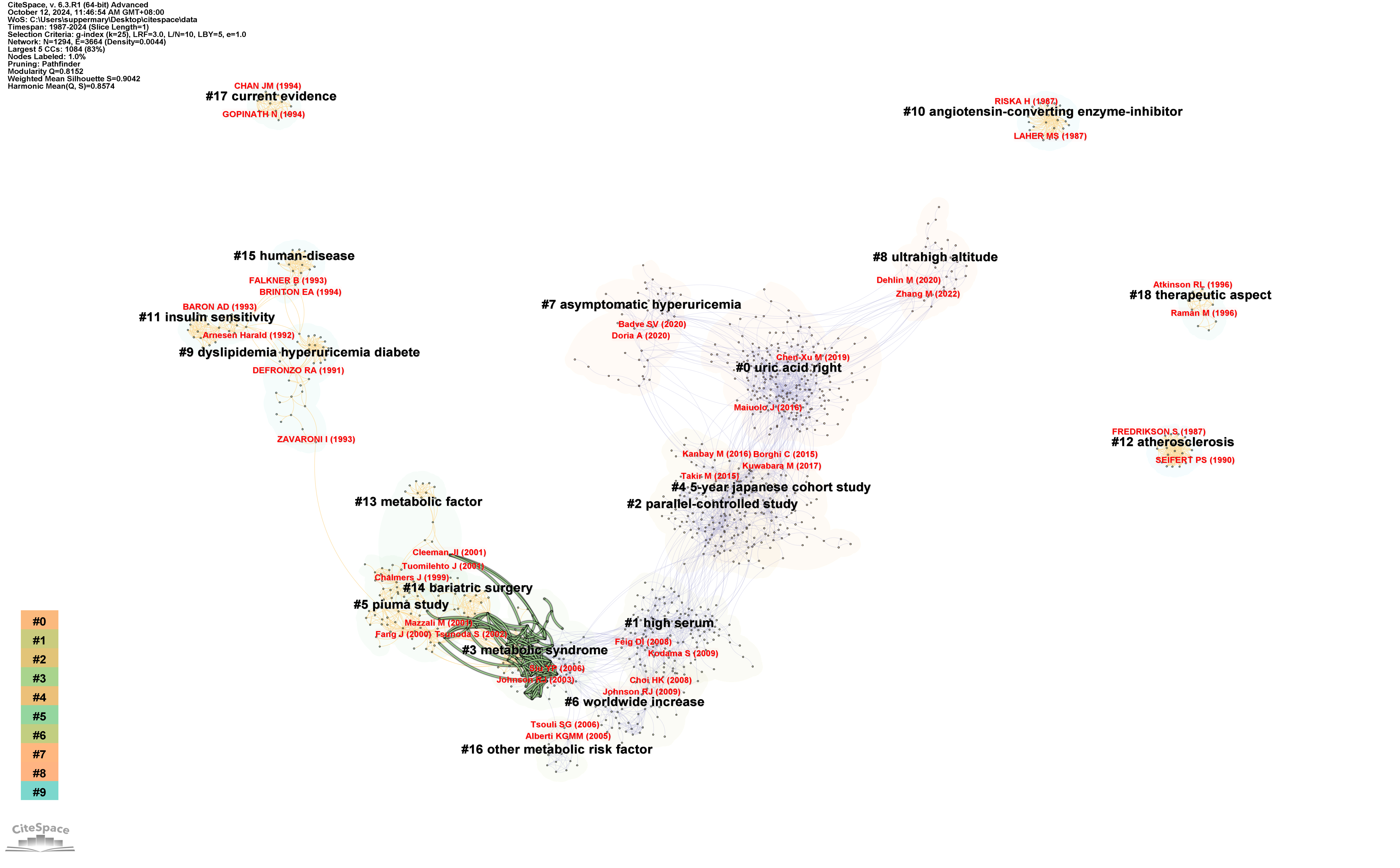

Supplement: Supplementary file 6 [file Supplementaryfile1.zip › Supplementary material Annex 1/2006.png]

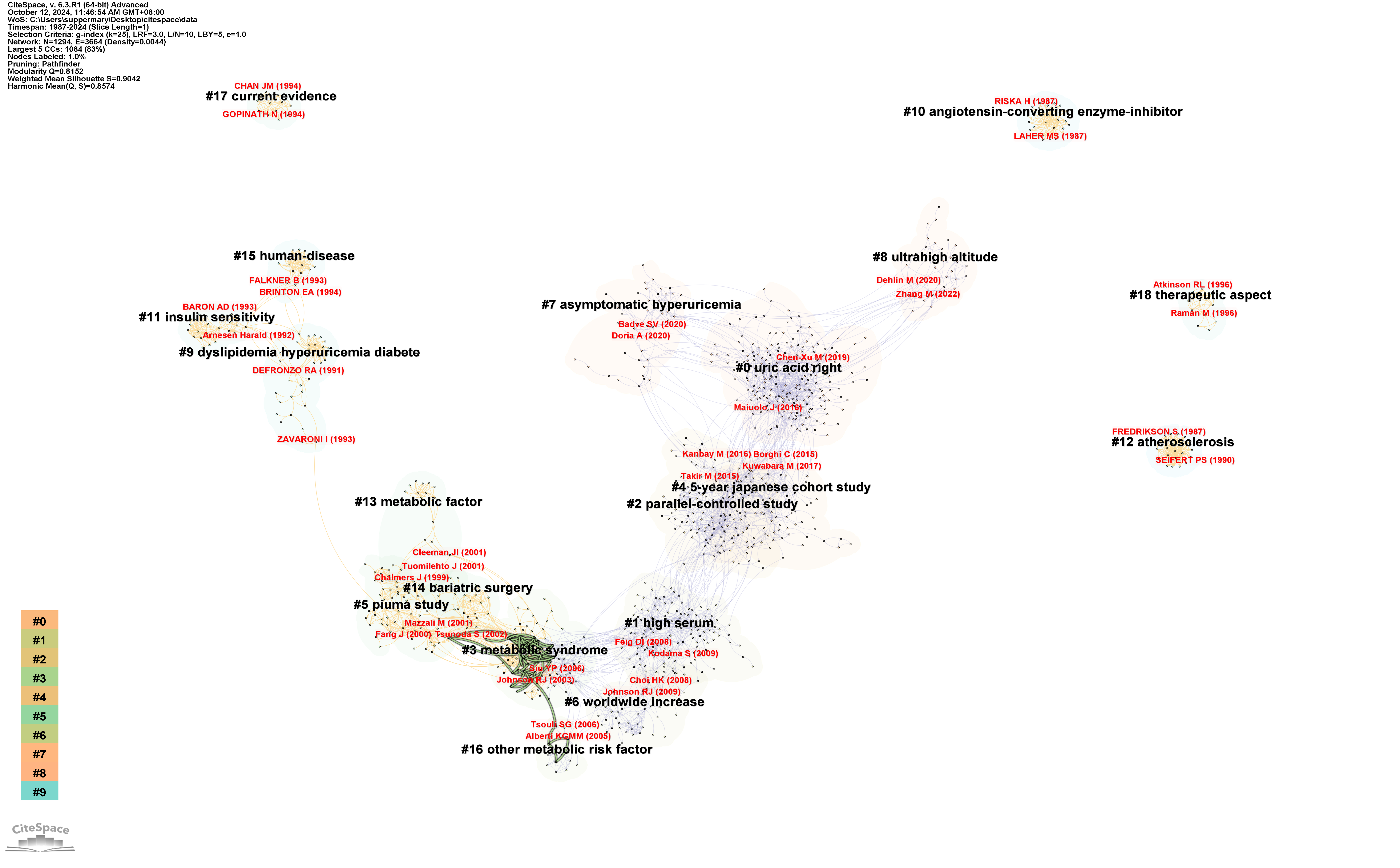

Supplement: Supplementary file 6 [file Supplementaryfile1.zip › Supplementary material Annex 1/2007.png]

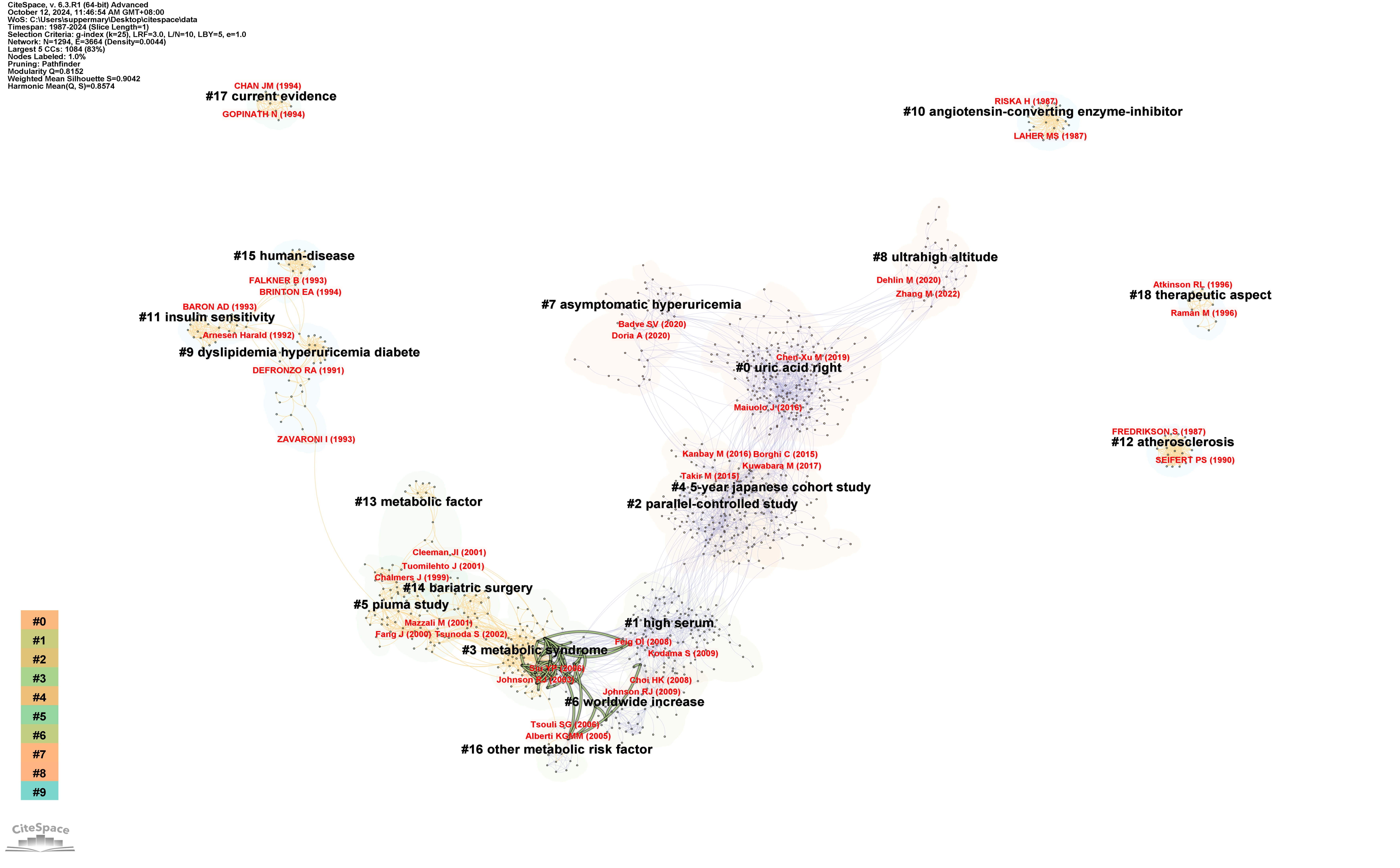

Supplement: Supplementary file 6 [file Supplementaryfile1.zip › Supplementary material Annex 1/2008.png]

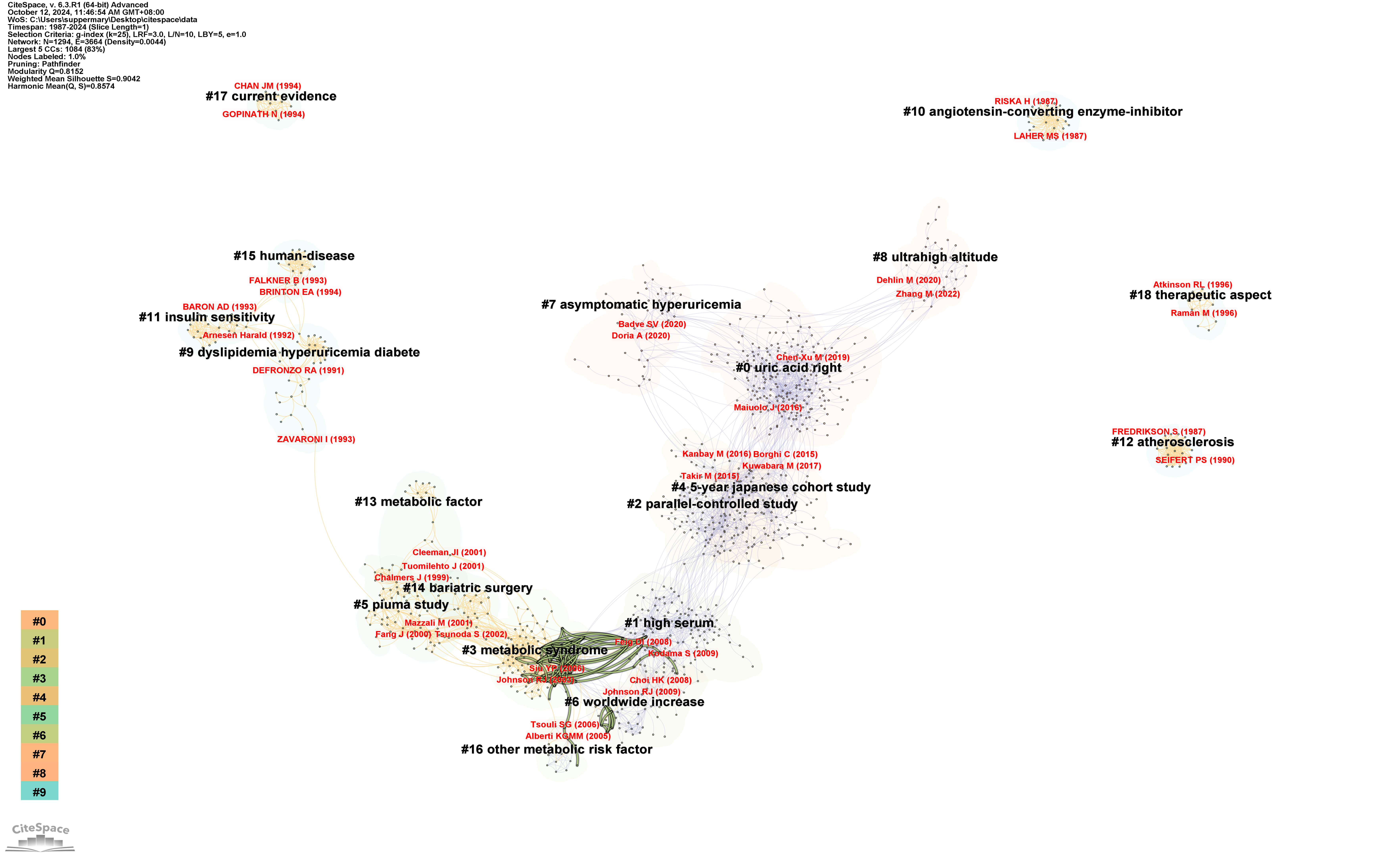

Supplement: Supplementary file 6 [file Supplementaryfile1.zip › Supplementary material Annex 1/2009.png]

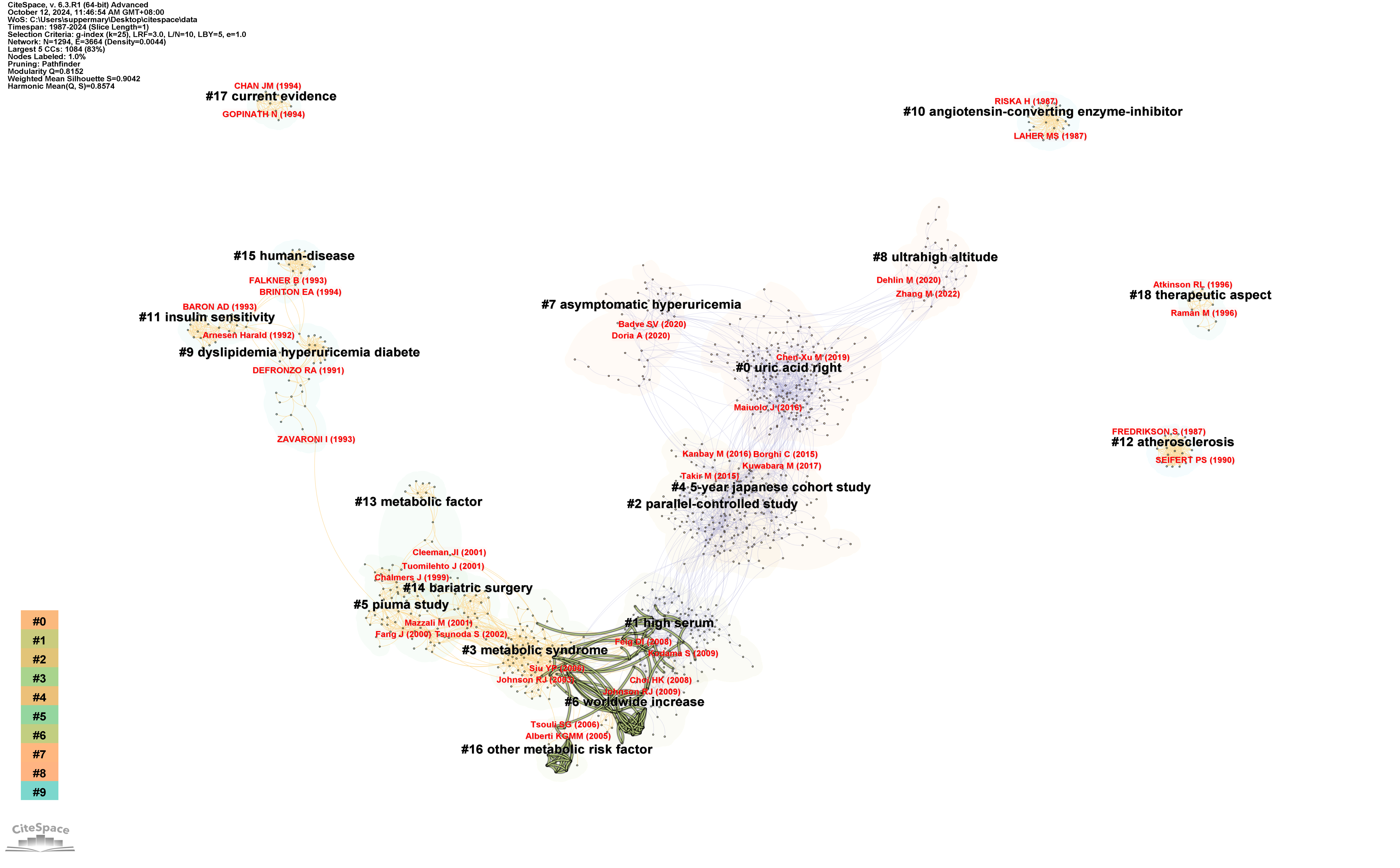

Supplement: Supplementary file 6 [file Supplementaryfile1.zip › Supplementary material Annex 1/2010.png]

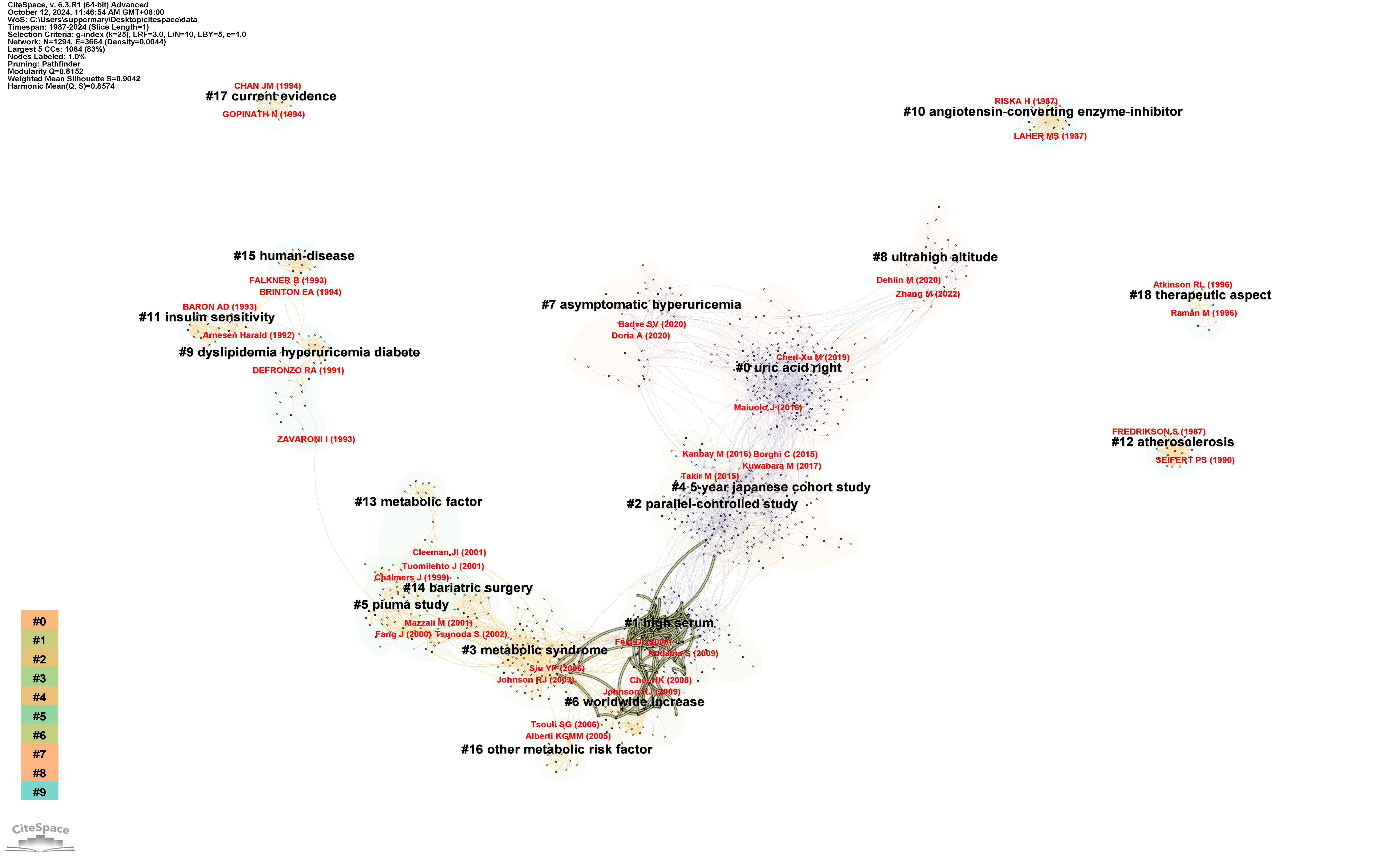

Supplement: Supplementary file 6 [file Supplementaryfile1.zip › Supplementary material Annex 1/2011.png]

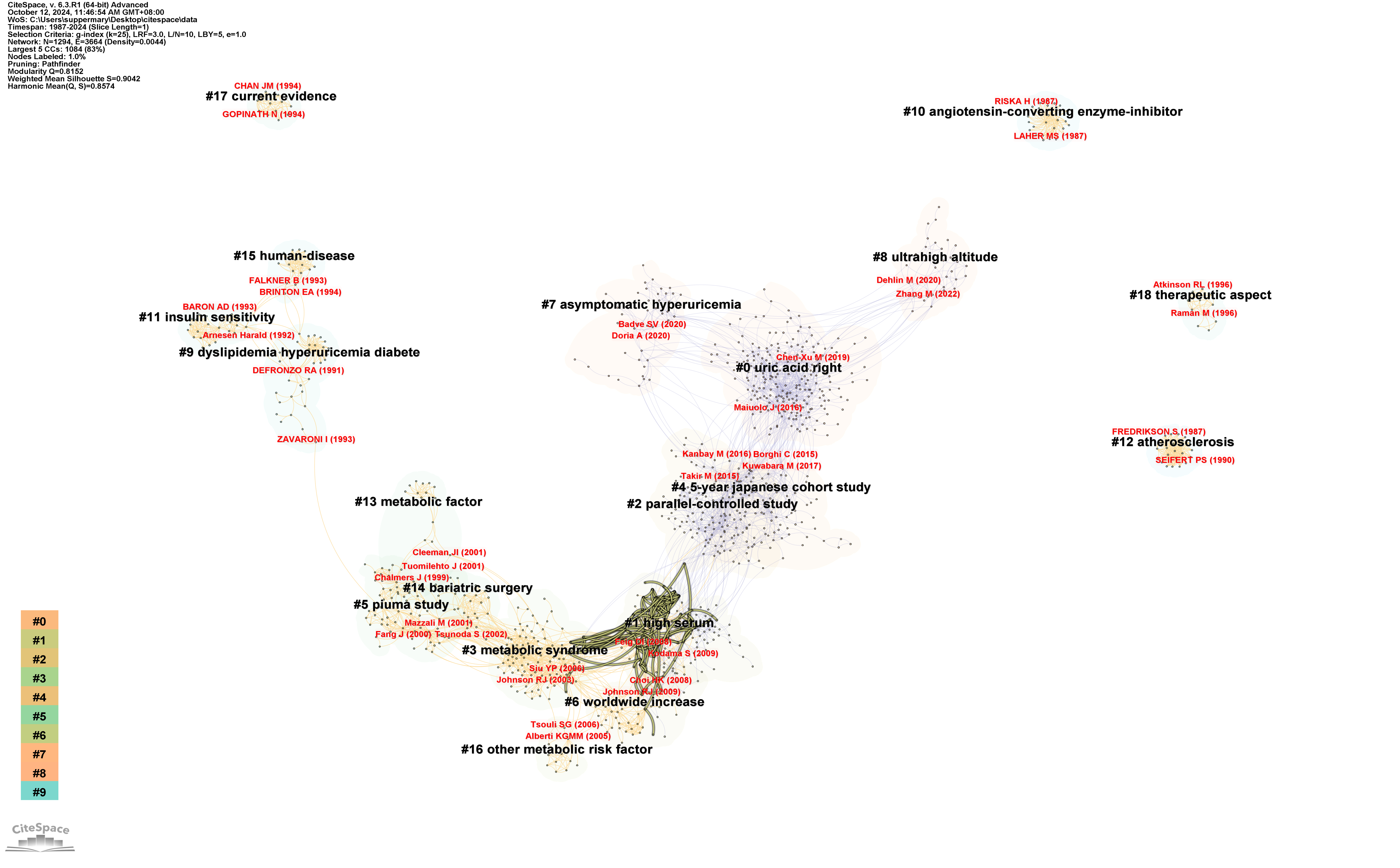

Supplement: Supplementary file 6 [file Supplementaryfile1.zip › Supplementary material Annex 1/2012.png]

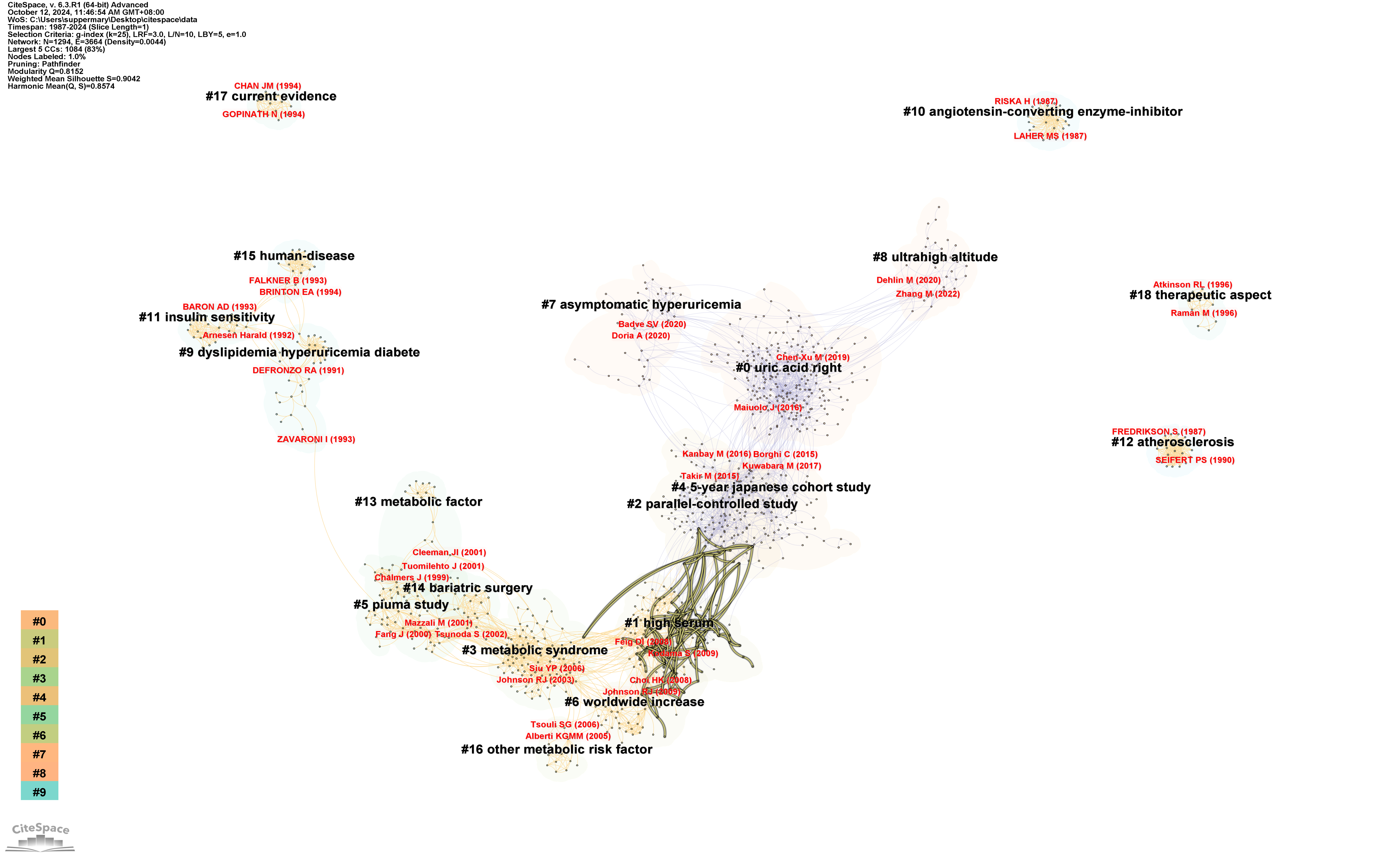

Supplement: Supplementary file 6 [file Supplementaryfile1.zip › Supplementary material Annex 1/2013.png]

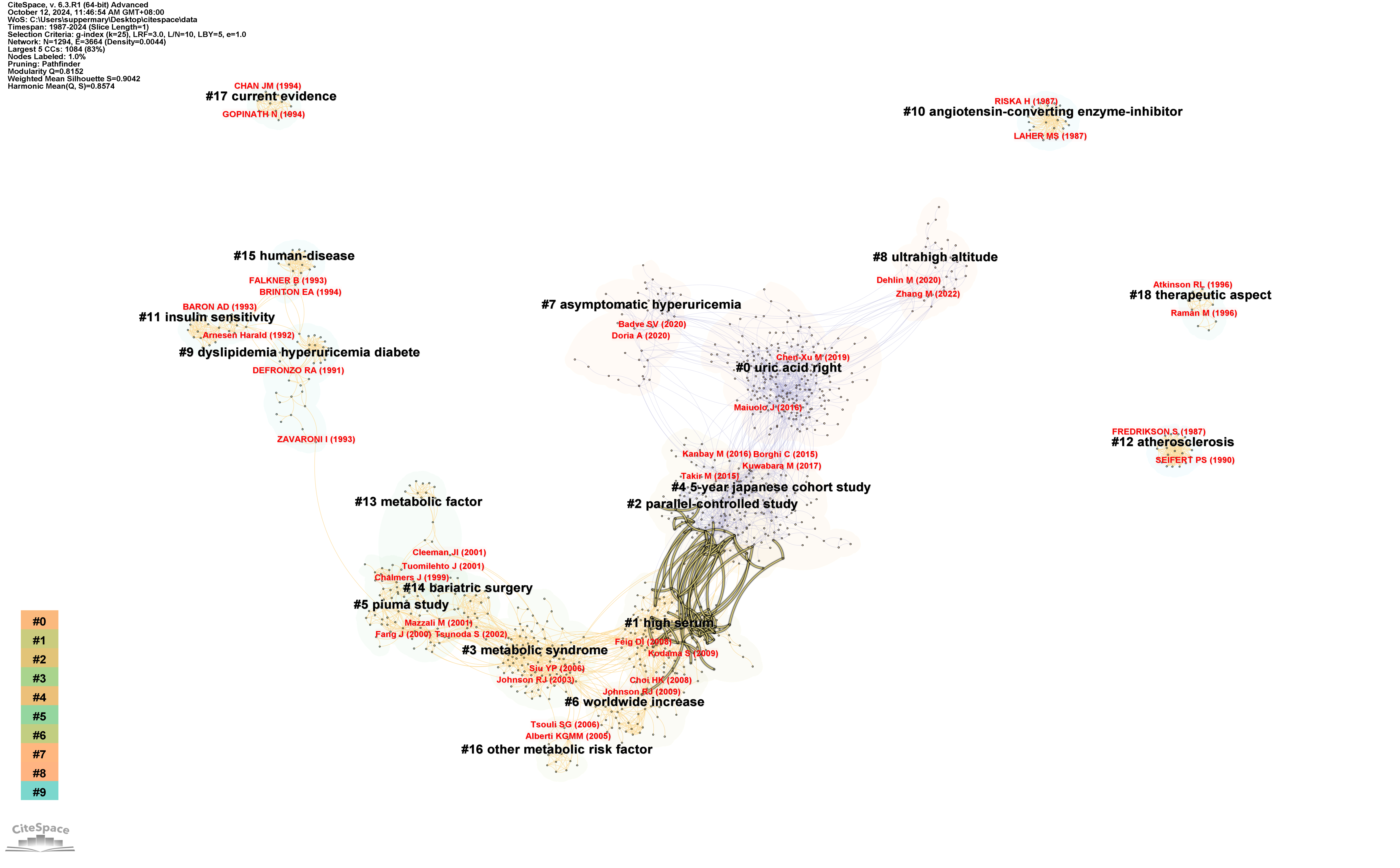

Supplement: Supplementary file 6 [file Supplementaryfile1.zip › Supplementary material Annex 1/2014.png]

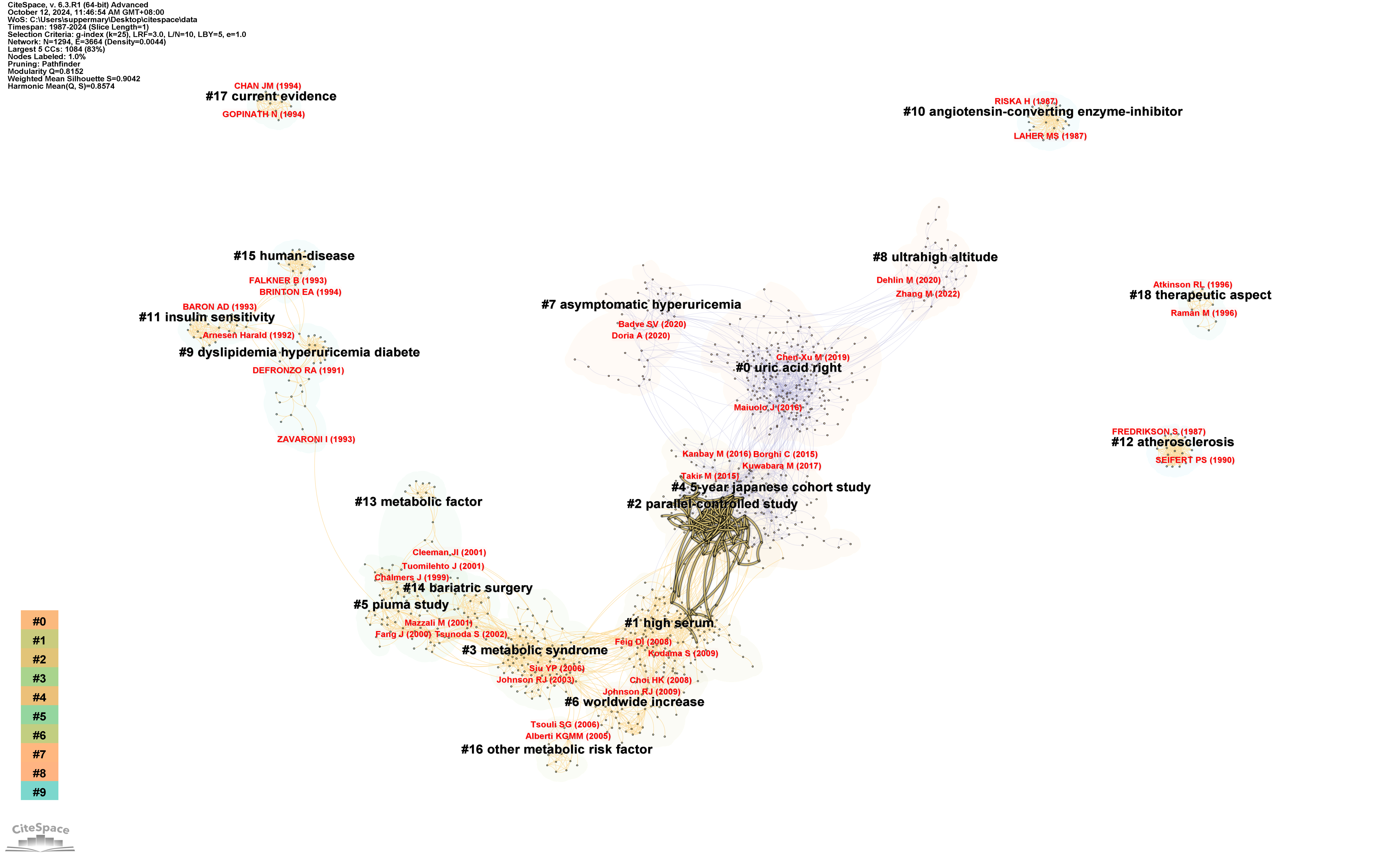

Supplement: Supplementary file 6 [file Supplementaryfile1.zip › Supplementary material Annex 1/2015.png]

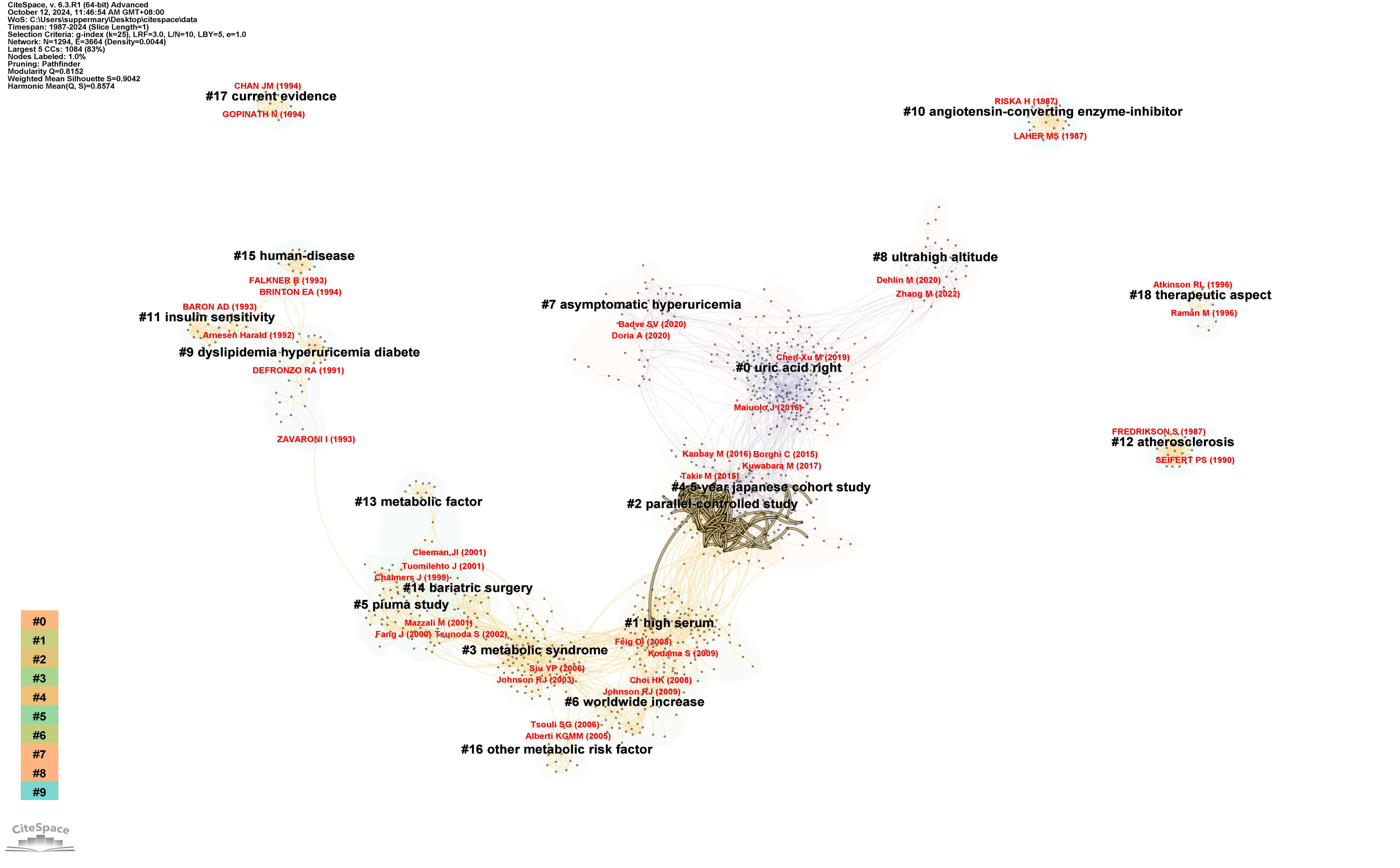

Supplement: Supplementary file 6 [file Supplementaryfile1.zip › Supplementary material Annex 1/2016.png]

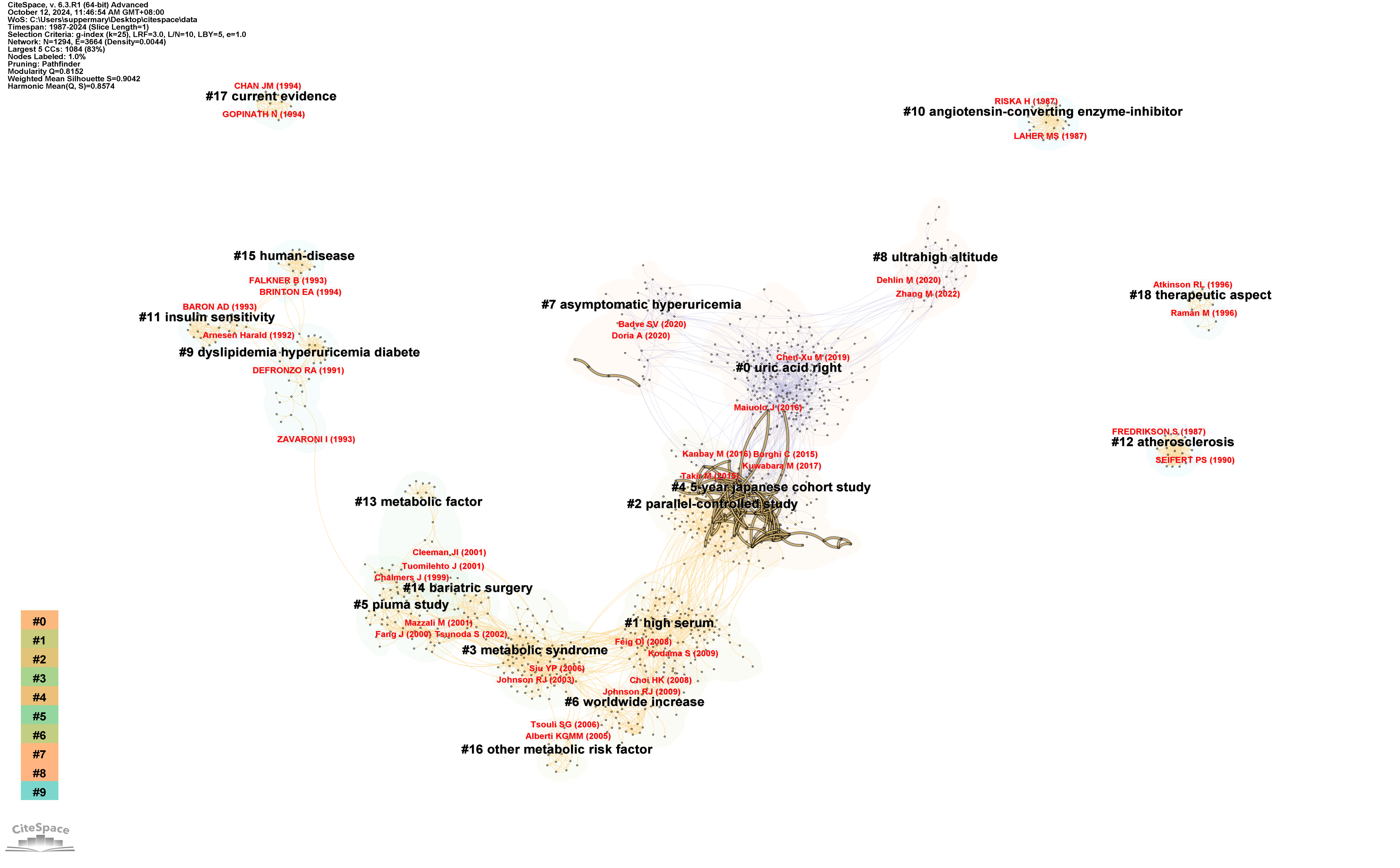

Supplement: Supplementary file 6 [file Supplementaryfile1.zip › Supplementary material Annex 1/2017.png]

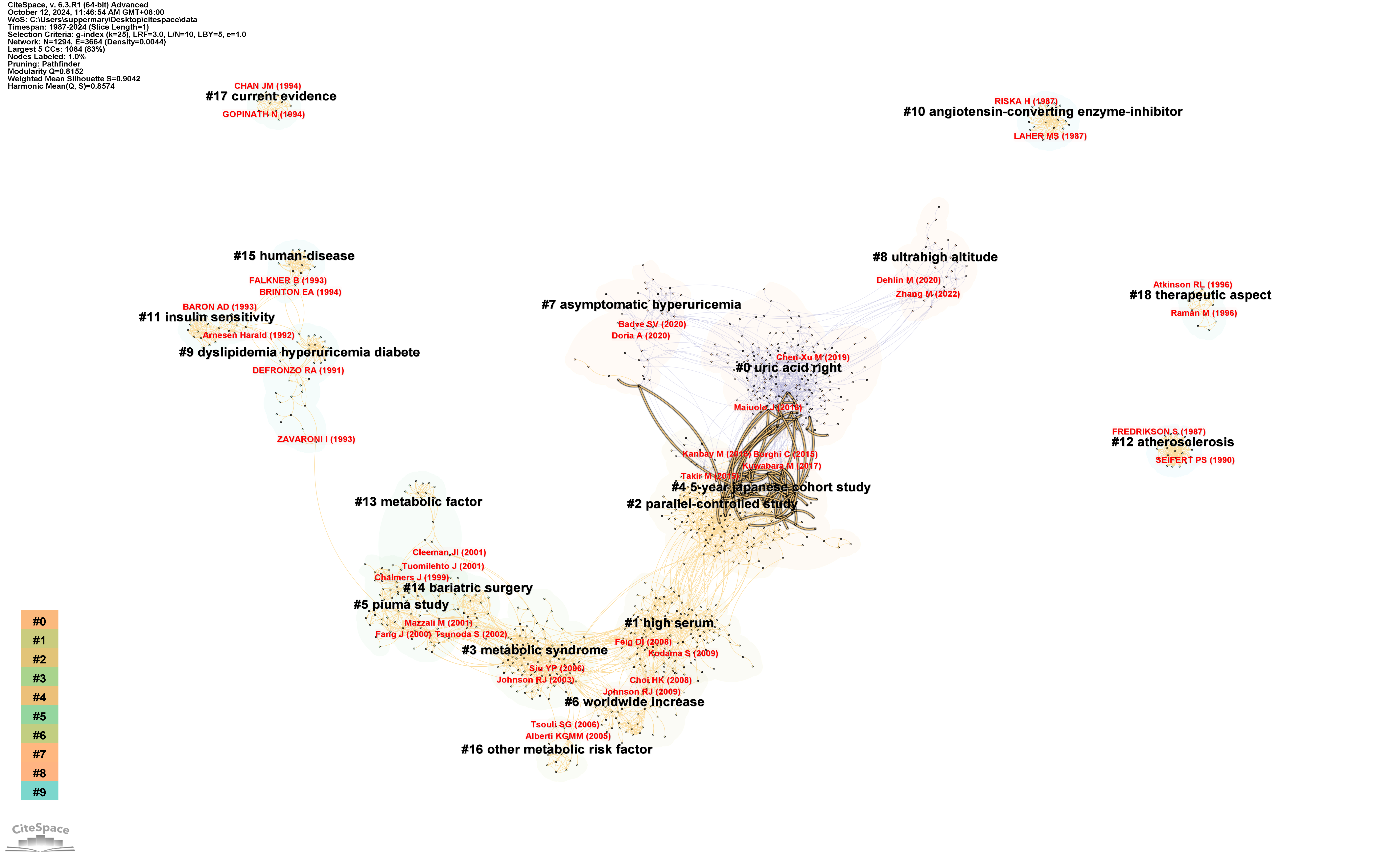

Supplement: Supplementary file 6 [file Supplementaryfile1.zip › Supplementary material Annex 1/2018.png]

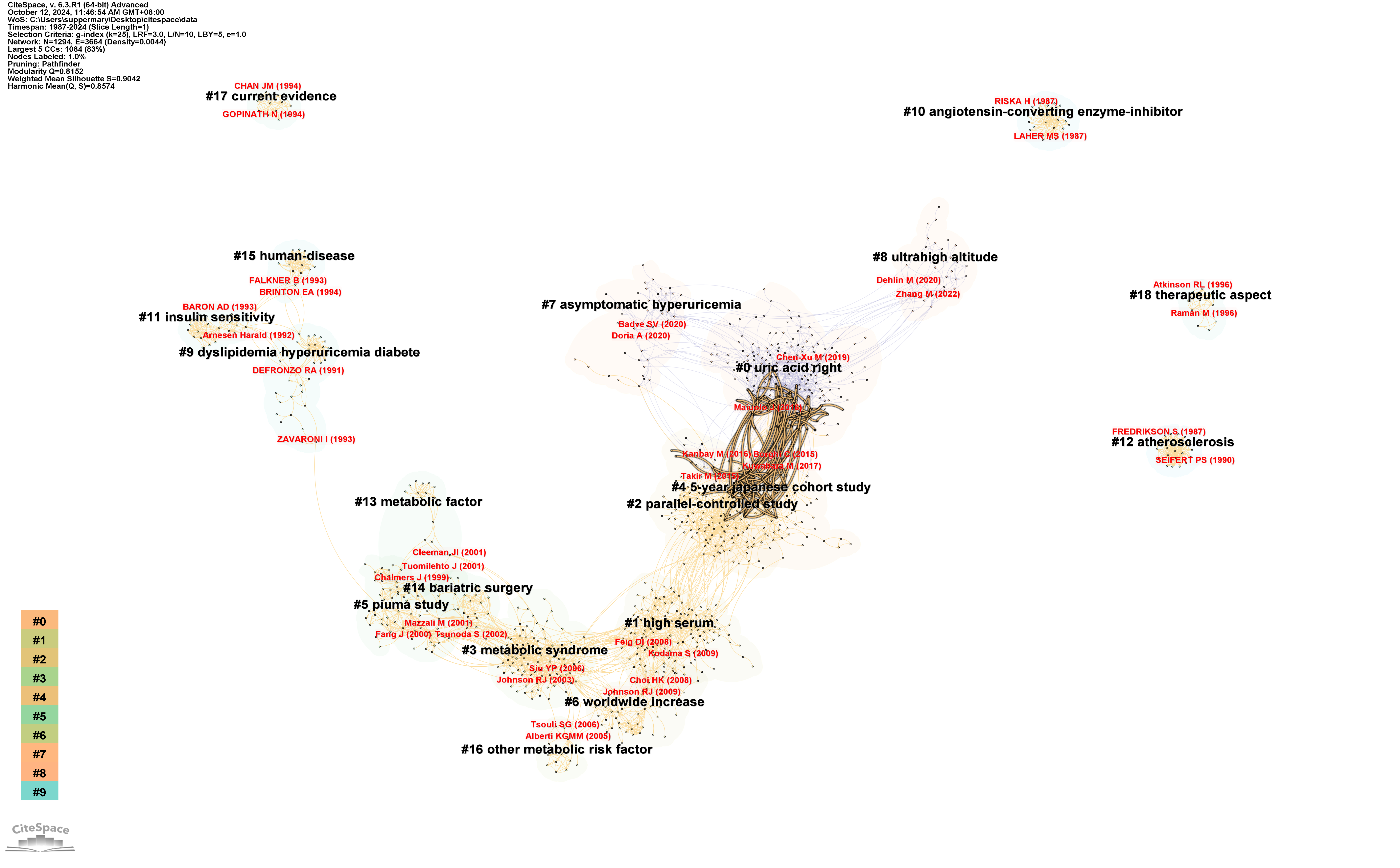

Supplement: Supplementary file 6 [file Supplementaryfile1.zip › Supplementary material Annex 1/2019.png]

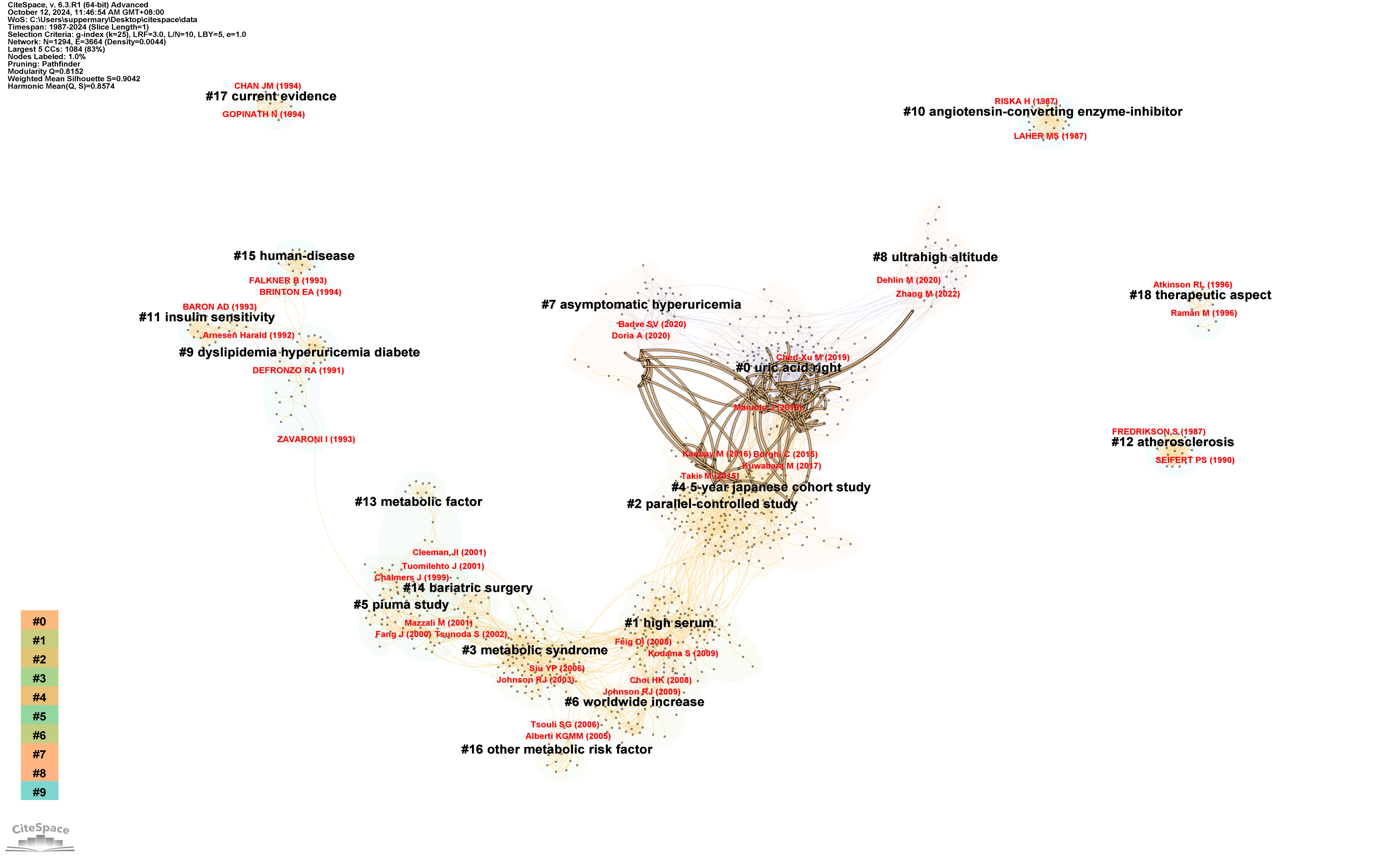

Supplement: Supplementary file 6 [file Supplementaryfile1.zip › Supplementary material Annex 1/2020.png]

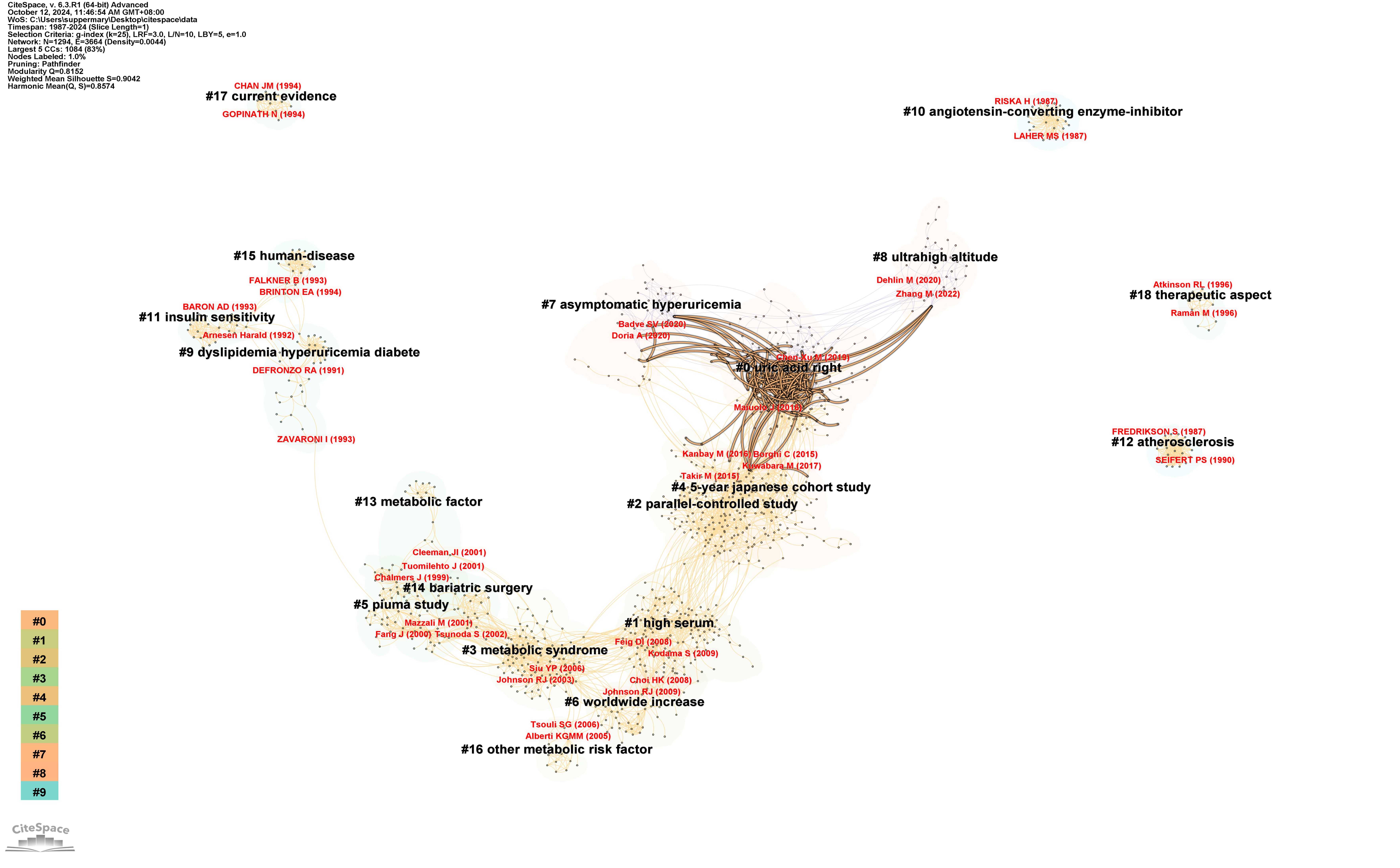

Supplement: Supplementary file 6 [file Supplementaryfile1.zip › Supplementary material Annex 1/2021.png]

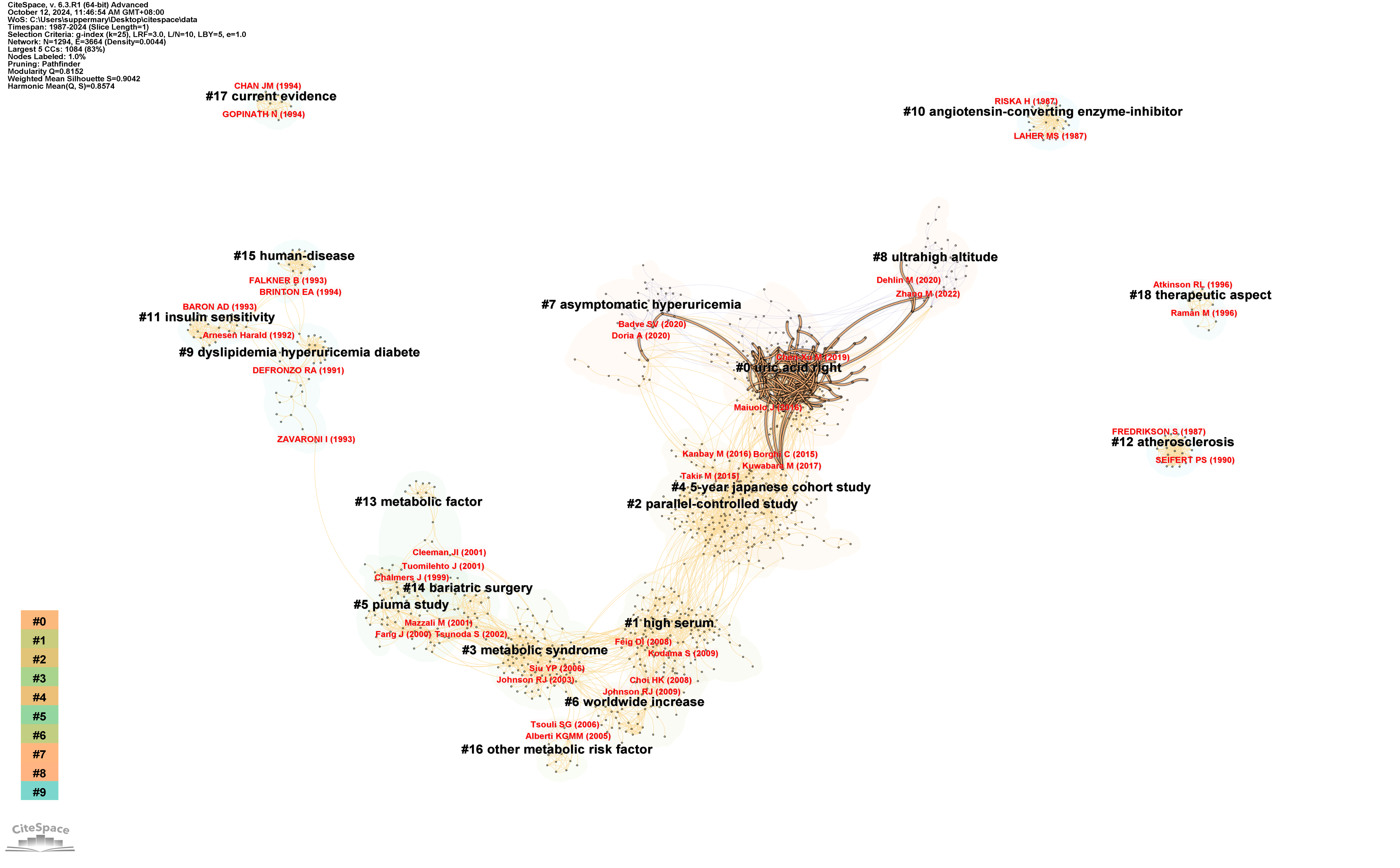

Supplement: Supplementary file 6 [file Supplementaryfile1.zip › Supplementary material Annex 1/2022.png]

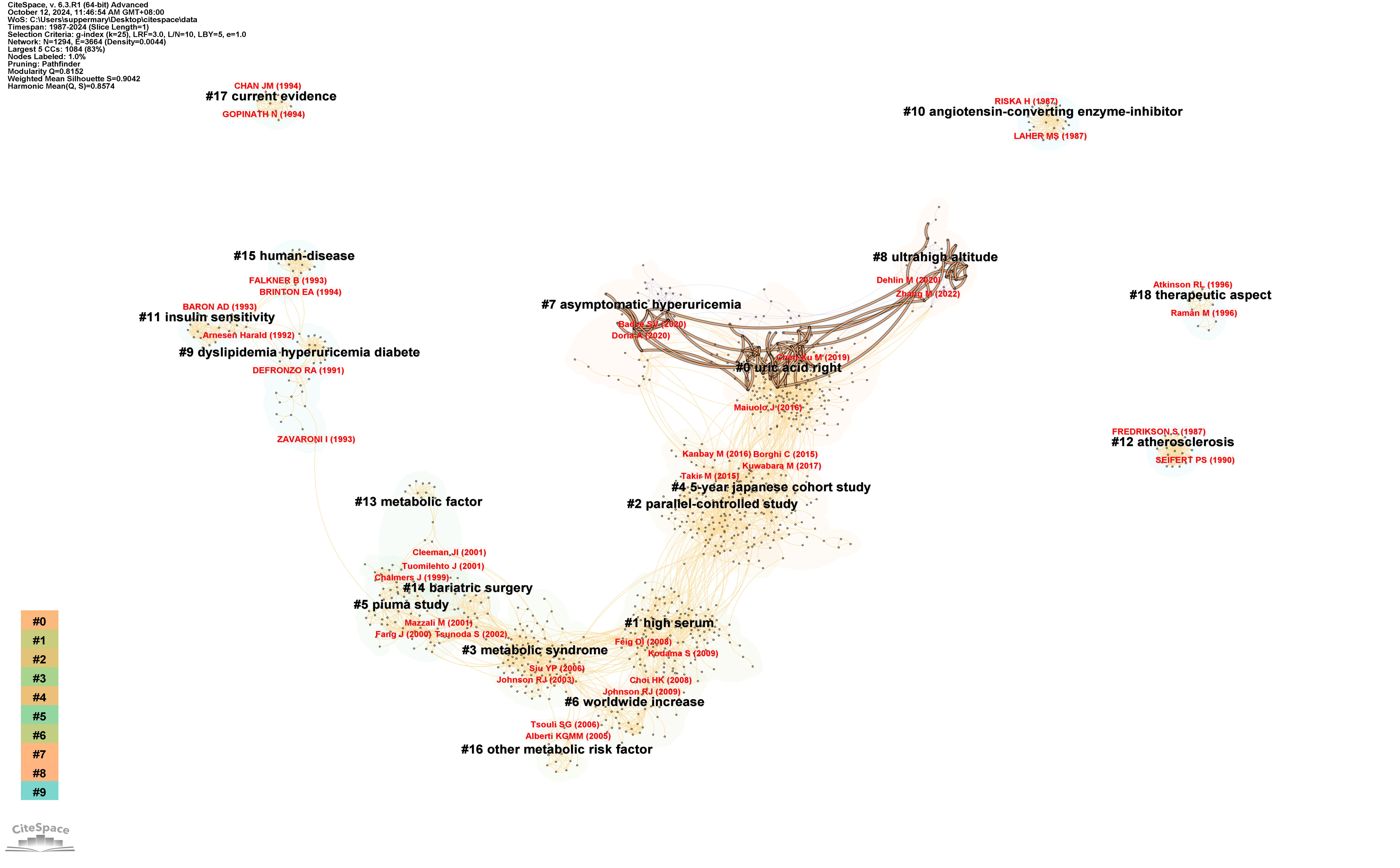

Supplement: Supplementary file 6 [file Supplementaryfile1.zip › Supplementary material Annex 1/2023.png]

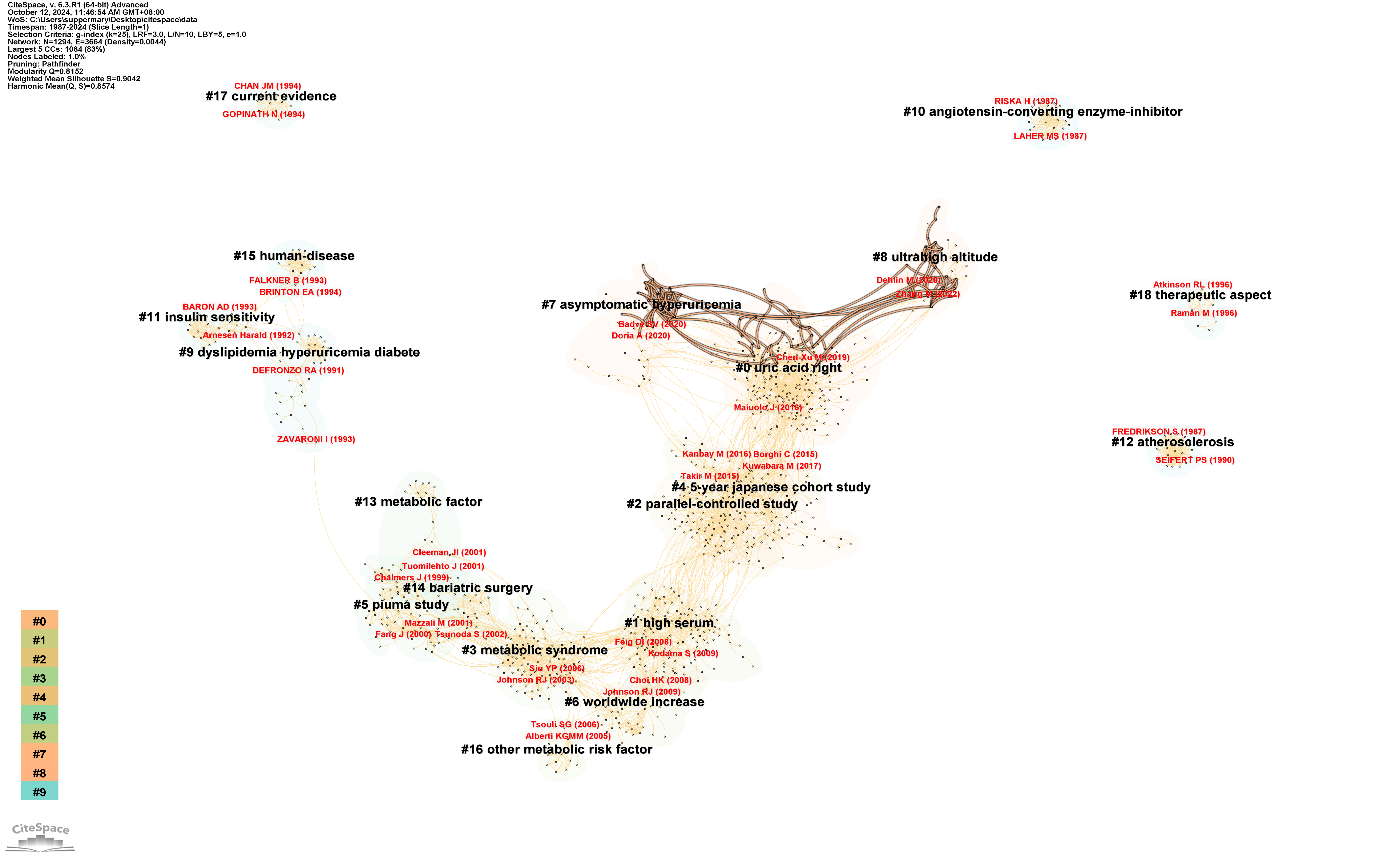

Supplement: Supplementary file 6 [file Supplementaryfile1.zip › Supplementary material Annex 1/2024.png]
